# Evaluation of the Implementation of a Home-Based Exercise Training Program for People With COPD: A Mixed-Methods Study

**DOI:** 10.3389/fresc.2021.743588

**Published:** 2021-10-26

**Authors:** Tamara Cerini, Ramona Kunz, Kaba Dalla Lana, Thomas Radtke, Ashley Polhemus, Milo A. Puhan, Anja Frei

**Affiliations:** Epidemiology, Biostatistics and Prevention Institute, University of Zurich, Zurich, Switzerland

**Keywords:** COPD—chronic obstructive pulmonary disease, exercise training, home-based, minimal equipment, evaluation, implementation, mixed-methods, adherence

## Abstract

**Introduction:** Recently, we developed a home-based, minimal-equipment exercise training program HOMEX for people with chronic obstructive pulmonary disease (COPD) and tested its effectiveness over 1 year in a randomized controlled trial. The aims of the current study were to evaluate the implementation of HOMEX from the perspectives of all involved persons and to optimize the program to ensure its long-term sustainability.

**Methods:** In this mixed-methods study, we used qualitative and quantitative approaches to evaluate the implementation of the intervention on the level of patients with COPD and coaches who provided the intervention and relevant stakeholders. To assess the implementation outcomes dose, reach, fidelity, and adherence, we summarized information recorded in the notes of the coaches and the diaries of patients, complemented with results from qualitative assessments. To assess acceptability and appropriateness, we conducted surveys with patients and coaches, and semistructured interviews with selected patients, coaches, and stakeholders.

**Results:** The coaches delivered the three home visits with one exception according to the protocol (fidelity). Of the 53 intervention group participants, 37 (70%) conducted HOMEX training until the end of the study and 43 (79%) trained for at least 10 months. The exercise behaviors of the participants could be separated into the phases “Starting the training and stabilizing into regular training routine” and “Managing training disruptions” (adherence). Overall, patients, coaches, and stakeholders conveyed a very high “acceptability” of HOMEX, noting the home-based aspect as a particular strength and interaction with other patients as future need. All involved groups perceived the strength-training exercises as appropriate, efficient for people with COPD, and relevant to maintain improvements after pulmonary rehabilitation. The most important facilitators of the patients for long-term motivation were self-perceived improvement in strength, supervision by a coach, and integration of the training in daily routine. Based on these insights, we redesigned and reworded the exercise cards, introduced three new exercises, and refined the training book.

**Discussion:** The results of this study provided insights of the involved persons in the frame of the HOMEX intervention implementation with a particular focus on the long-term training behavior of the participants and their perception and experience with the exercise program. These findings enabled us to optimize the training material and adapt the structure of the program for sustainable further use in clinical and other settings.

## Introduction

Exercise training is a central component of pulmonary rehabilitation (PR) (1, 2). It is well-established that exercising with or without other elements of PR increases physical fitness, improves quality of life, and reduces troublesome symptoms in individuals with chronic obstructive pulmonary disease (COPD) (3). However, despite strong evidence on the positive effect of PR on clinical outcomes, relatively few persons with COPD participate in PR programs. For those who complete such programs, it remains challenging to integrate the recommended exercises into their daily lives and to obtain long-term benefits (4). To overcome the known barriers of the patients, such as travel to centers or disruption of routine (5), and as alternatives for patients, home-based programs are becoming increasingly popular. Limited, though promising, evidence shows similar outcomes of home-based programs compared with traditional center-based PR in people with COPD (6). Most programs last between 6 and 10 weeks (7–9), though they vary in content and delivery modus, and the studies assessed midterm outcomes after 6 months. One study with 12-month follow-up assessments showed that gains could not be maintained long-term (7), similar to center-based programs (1, 10).

Against this background, we developed the HOMEX (HOMe-EXercise) strength training program for patients with COPD (11). We deliberately focused on the exercise component of PR and did not consider other self-management or education elements. Our emphasis was on the long-term maintenance of training, and we tailored the program to become a habit in the usual environment of people. The program is home-based, structured, and requires only a chair and resistance bands. It is supervised by a personal coach (healthcare professional) who visits the participant at home and regularly follows up through phone calls. The effectiveness of the HOMEX program over 1 year has recently been assessed by two randomized controlled trials (RCT), HOMEX-1 and HOMEX-2. The HOMEX-1 RCT included 123 patients with COPD who were recruited after the completion of PR in Swiss PR clinics. The results showed that the program effectively improved functional exercise capacity assessed by the 1-min sit-to-stand test after 1 year and that the program was safe. However, no statistically significant effects were shown for the primary outcome dyspnea or for other outcomes. The vast majority of the multimorbid and severely ill participants subjectively perceived positive effects that they attributed to the training (submitted, NCT03461887). The HOMEX-2 RCT included COPD patients who did not undergo PR within the previous year. HOMEX-2 is also completed and data analysis is ongoing (NCT03654092).

It is a great challenge in healthcare research that innovations and interventions often do not find their way into clinical practice or the daily lives of patients after they were developed and initially tested in trials. With this study, we addressed exactly this gap. We aimed to thoroughly evaluate the implementation of the HOMEX exercise program in the frame of the HOMEX-1 RCT over the course of the study year and subsequent months, considering the perspectives and experiences of the involved persons, and potential future providers. Specifically, we aimed to capture and describe how the intervention was delivered, accepted, and perceived. A particular focus was to gain insights into the motivation of participants to maintain long-term training and into the relevance of different intervention elements. The results provided guidance to optimize the program materials and to ensure their further use and long-term sustainability.

## Materials and Methods

### Theoretical Framework and Study Design

In this mixed-methods study, we used qualitative and quantitative approaches to evaluate the implementation of the HOMEX intervention from the perspectives of intervention group participants, the coaches who provided the intervention, and other relevant stakeholders, such as healthcare professionals from the involved rehabilitation clinics and potential future providers. The design was guided by the Medical Research Council (MRC) guidance on the process evaluation of complex interventions (12) as overarching framework and by the work by Proctor et al. (13) regarding the implementation outcomes. We aimed to understand how the intervention was delivered (implementation outcomes reach, dose, fidelity, and adherence) and how it was accepted and perceived by the involved persons (implementation outcomes acceptability and appropriateness), with a particular focus on the insights of the participants, and considering the context factors. The study was approved by the local ethics committees (BASEC-Nr. 2017-02092). All patients gave written informed consent.

### HOMEX Exercise Training Program and Application Within the HOMEX-1 RCT

**Figure 1:**
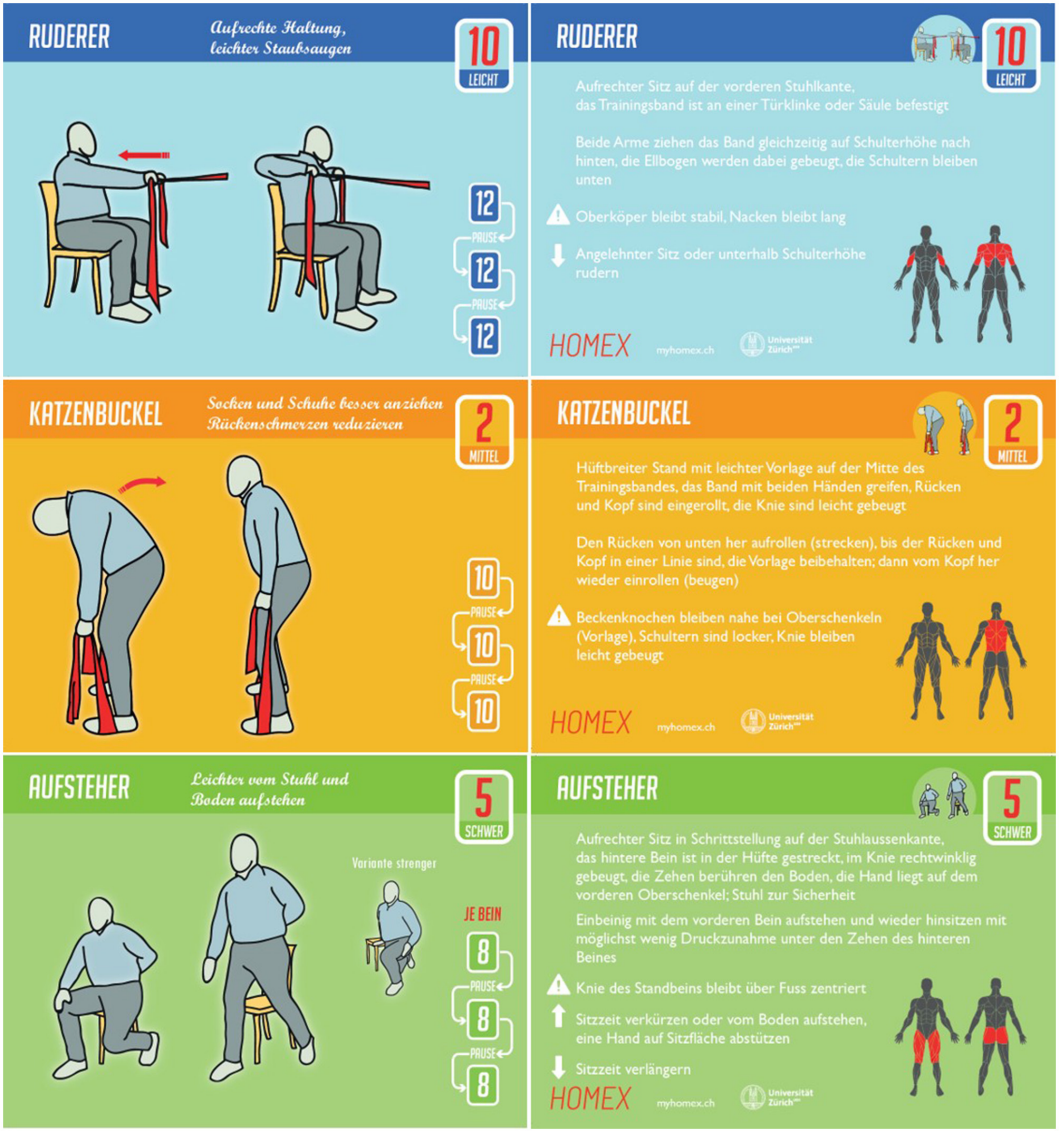
Examples of three exercise cards.

The HOMEX exercise training program was developed by our interdisciplinary team (i.e., physiotherapist, exercise scientist, medical doctor, epidemiologist, psychologist, and visual artist) tailored to persons with COPD. It includes whole body exercises, i.e., trunk, upper limb, and lower limb exercises, modified in three different intensity levels as well as general warm-up and specific stretching exercises. The program is home-based, requires only minimal equipment (a chair and elastic bands), and can be conducted in either seated or upright positions. It was designed to be easily integrated into daily routines over the long term by developing habits to trigger daily completion. It is performed 6 days a week for about 20 min daily (four exercises of ~5 min each, excluding warm-up and stretching). Thirty-eight thoughtfully designed, high-quality illustrated training cards (examples see Figure 1; ~24 × 18 cm, printed on heavy cardstock) depict a model with a body shape realistic for a person with COPD. The cards provide detailed performance instructions, suggested training volume and intensity, and a concrete benefit when the exercise is conducted regularly (e.g., “To reduce back pain and lifting loads more easily”). The colorful training cards, provided with a stand to allow sight during the training, are complemented with a training book in which the participants record their daily training. The training book aims to guide and motivate the participants and contains motivational elements such as establishing short- and long-term individualized goals and rewards. The delivery of the HOMEX training was intentionally designed in this traditional, tactile way against a landscape of digital or DVD-type instruction, which we initially considered but decided against due to the feedback of the patients. This allows the participant to take physical objects into their hands, work with them as they complete the exercises, and be free of potential distractions that arise when opening a smartphone app (e.g., notifications from other apps, text messages, and so on).

The self-directed program is supervised by a personal coach trained in the HOMEX training elements and in motivational interviewing techniques. The coach visits each participant at home three times during the first 3 months to establish the training setting and to instruct and support the participants in conducting the program. The in-person visits are supplemented by 17 scheduled telephone calls over 1 year. For each participant contact, the coach is provided with guidance documents including algorithms describing how to proceed in specific situations. In addition, a relative, friend, or a close person is involved as a “sparring” partner to support the participant, and the general practitioner is informed about the HOMEX training of his/her patient. The intervention material, administration, and home visits were carefully piloted with older persons with COPD.

The HOMEX-1 trial to assess the effectiveness of the HOMEX intervention took place between January 2018 and March 2020. Patients with COPD from three inpatient and one outpatient Swiss PR clinics were included in the study and randomized to the intervention (HOMEX program) or control group (usual care). Assessments took place in the clinics before randomization and after 1 year. The HOMEX coaches who delivered the program were healthcare professionals from the clinics specifically trained by the master coach (KDL) during a basic training and an update session a few months later (half a day each). The master coach accompanied all coaches once at a home visit to ensure the intervention was conducted according to the protocol. During the time of the intervention, the HOMEX coaches were provided with “HOMEX-magic,” an email exchange system moderated by the master coach, to connect and exchange experience and to get support. More detailed information is provided elsewhere (11).

### Study Population

The current study addressed three groups of persons who were involved in the program. On the part of the patients, we addressed persons who participated in the intervention group of the HOMEX-1 trial. Out of those, we additionally selected participants who did not prematurely stop the intervention for in-depth interviews. Second, we addressed the HOMEX coaches who delivered the intervention. Finally, we addressed relevant stakeholders. These stakeholders represent persons who would be responsible for implementing HOMEX in rehabilitation clinics and other settings.

### Implementation Outcomes and Measurements

To review how the intervention was delivered in practice, we assessed “dose” (the quantity of intervention implemented) through the reports of the coaches regarding the number and duration of home visits and telephone calls and the materials delivered to each participant. “Reach” (the extent the target group came into contact with the intervention and how) was summarized by results from the qualitative assessments with patients. We assessed “fidelity” (whether the intervention was delivered as intended) by comparing the planned and implemented intervention elements (structured protocols of coaches on visits and calls). The “adherence” of patients to the intervention was assessed through daily training records, training breaks, and set and reached goals and rewards. We classified a week to be “adherent” if the participants conducted at least two exercises on 3 days of the given week. We crosschecked the entries of the patients with the protocols of the coaches and reported (serious) adverse events which prevented the participants from training.

At the 12-months follow-up visit of the HOMEX-1 trial, the intervention group participants filled-in a satisfaction questionnaire and we conducted a short interview with them (most of these results presented in the HOMEX-1 RCT manuscript on effectiveness). Based on these insights, we developed an interview guide and conducted semistructured in-depth interviews with selected participants to investigate “acceptability” (perception among involved persons that the intervention is agreeable, palatable, or satisfactory) and “appropriateness” (perceived fit, relevance, or compatibility of the intervention for a given practice setting). Many COPD patients suffer from comorbidities and usually experience limitations due to COPD and other health problems, which make training challenging. Our main aim of the in-depth interviews was to get insights on the long-term motivation and on how the participants succeeded in coping with difficult times and maintaining the training. Therefore, we included persons who did not prematurely stop the intervention before the end of the study and consecutively asked participants from the clinics whether they agreed to the interview. In addition to their long-term motivation, we also explored participants' reasons for participation, expectations, and feedback on the overall structure of HOMEX and specific program elements (exercises, cards, coach, home visits, training book, and calls). We supplemented the feedback on the program elements with results from the satisfaction questionnaire filled-in by all participants at RCT end regarding the assessment of the specific features of the program and the most helpful elements for training support (results not presented yet).

All the 12 involved coaches completed a written questionnaire regarding their experiences and satisfaction with the program, their assessment of specific elements (scales 0–10, from very negative to very positive), and their opinions on further delivery of the HOMEX-program. We then conducted semistructured interviews with the seven most involved coaches to gain deeper insights and to optimize the program elements and materials. For the interview, we selected those coaches either who coached at least five patients or who had additional coordination and supervising roles in their clinics and for the HOMEX-1trial. Finally, we conducted semistructured interviews with six stakeholders who represent current and potential future providers of the program. We selected, on the one hand, the responsible healthcare specialists from the participating rehabilitation clinics and on the other hand persons from other Swiss healthcare settings who could be the future provider of the HOMEX intervention. We aimed to learn about contextual factors and to plan concrete sustainable maintenance and further implementation of the program in different settings. Table 1 overviews the implementation outcomes and how they were assessed.

**Table 1:** Overview on implementation outcomes and assessment methods.

**Outcomes**	**Population assessed**	**Indicators / assessed constructs**	**Source of data**	**Time of assessment**
Dose	Coaches	Number of home visits and telephone calls, duration of home visits and telephone calls, materials delivered	Structured coaching protocols	During intervention
Reach	Selected participants	Reasons for participating in the study / exercise program	Semi-structured interview	7 months after RCT end
	Coaches	Perceived reasons for patients for participating in HOMEX	Semi-structured interview	10–12 months after RCT end
Fidelity	Coaches	Comparison of originally planned and actually implemented intervention elements	Comparison of structured coaching protocols with planned elements	During intervention
Adherence	Participants of Intervention group (all)	Adherence to the intervention: Actual conducted daily HOMEX trainings	Reports in training books: Daily exercises, breaks, goals, rewards	During intervention
Acceptability	Participants of Intervention group (all)	Satisfactionwith specific elements of the intervention	Satisfaction questionnaire	At 12-months follow-up study visit
Appropriateness	Selected participants	Insights on barriers and facilitators for program uptake and long-term maintenance and motivation, relevance of elements	Semi-structured interview	7 months after RCT end
	Coaches	Barriers/facilitators during coachingConcrete feedback on elementsContextual factorsThoughts on future provision of HOMEX	Questionnaire, semi-structured interview	10–12 months after RCT end
	Current and potential future providers	Perception of interventionThoughts on future provision of HOMEXInput for concrete planning of further implementation	Semi-structured interview	11–15 months after RCT end

### Analyses

Coaching protocols and questionnaire data were descriptively analyzed and responses to open-ended questions were categorized by conventional content analysis (14). Results of all analyses are presented as numbers and percentages or means and standard deviations/medians interquartile ranges.

**Figure 2:**
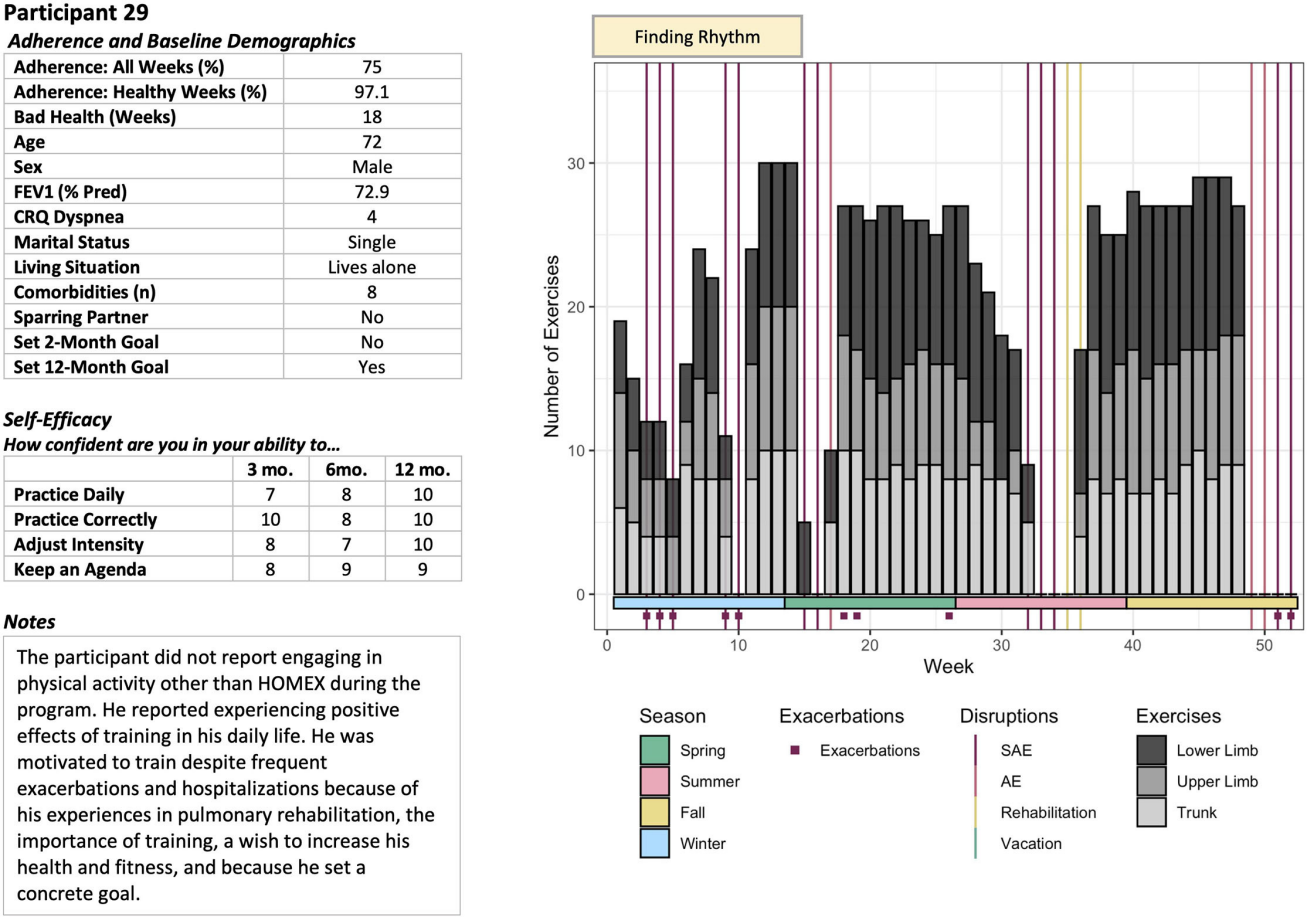
Example of patient adherence visualization (participant 29).

The adherence patterns of participants were identified through an adapted “grounded visualization” approach proposed by Knigge and Cope (15). This approach, originally developed for Geographical Information System (GIS) research, applies a “purposeful, recursive data exploration” to visualized quantitative data and contextual qualitative data. Our quantitative data included demographic characteristics of the participants and exercises reported in training books. Contextual and qualitative data included self-reported notes on adverse events, non-HOMEX activities, and environmental factors that affected the training of participants each week. The notes collected by HOMEX coaches during the calls were also analyzed. First, demographic, exercise, and adherence data were explored through scoping visualizations. Detailed visualizations described the demographic data of participants, COPD symptoms, self-efficacy, and contextualized exercise routines, and health events. An example is provided in Figure 2. We analyzed these visualizations according to grounded theory (15, 16). We conducted iterative open, axial, and selective coding of the dossiers to characterize patterns, noting week-to-week trends in the visualized data, and reviewing the raw contextual data of the participant during each round of coding. This analysis identified two distinct exercise patterns in the early months of the program. A Fisher exact test and resulting conditional maximum likelihood estimate of the odds ratio were used to quantify the relative odds of completing the study intervention based on these initial activity patterns.

The in-depth interviews with the participants were recorded, transcribed by a professional transcriber verbatim, and deidentified. We analyzed the material through qualitative content analysis using mixed deductive and inductive approaches (17). We first defined main categories according to the dimensions we wanted to explore. These included reasons for participation, expectations, long-term motivation, feedback on the structure of HOMEX, and on specific elements such as exercises, cards, coach, home visits, agenda, and calls. These categories were chosen to identify personal facilitators and barriers to participation as well as potential adaptations and considerations for future implementation. The coding was organized and systematically conducted using MAXQDA 2020. We coded the text line-by-line with the deductive coding system and remained flexible for inductive themes, and we allocated the quotes to the coding categories. For reliability purposes, the two coders (TC and AF) compared codes and categories after independently coding 25% of the interviews and discussed differences until a consensus was reached. We summarized the content by categories and subcategories and exemplified them with selected verbatim quotes. Data collection was terminated when saturation was reached, which was the case after nine interviews. The two women coders had 15 years (AF, trained psychologist, PhD) and 1 year (TC, trained physiotherapist, MSc) experience in qualitative research. Both researchers were not involved in the delivery of the intervention. The interviews lasted between 30 and 88 min.

The semistructured interviews with the coaches and stakeholders were recorded and analyzed by conventional content analysis (14) and then summarizing the relative frequencies of occurrence.

## Results

Fifty-three out of the 61 intervention group participants who were included in the HOMEX-1 trial completed the 12-month follow-up assessments. Of these, 49% were women. The average age was 66.2 (SD 8.2) years and the average Forced Expiratory Volume in 1 second (FEV_1_) in percent predicted was 41.8 (SD 15.3). All participants suffered from comorbidities. On an average, they reported 4.0 (SD 2.1) comorbidities, most frequently cardiovascular and musculoskeletal diseases. Participants were recruited from the inpatient setting after completion of PR, with one exception (outpatient setting). Overall, 37 (70%) participants performed the HOMEX training until the end of the study (12 months), and 42 (79%) trained for at least 10 months. Sixteen participants stopped the training on average after 28 weeks (SD 14.6), 11 due to health reasons (COPD related: exacerbation/dyspnea [*n* = 3], lung transplantation [*n* = 1]; non-COPD related: inflammation or pain in shoulder, elbow or hip [*n* = 3], poor general health condition[*n* = 1], heart palpitation[*n* = 1], not known [*n* = 2]). Four stopped for other reasons (preference of another training, only sporadically trained, no motivation), and one person did not start the training at all.

Nine patients with COPD participated in the in-depth interviews (six women, three men) out of the 37 persons who did not prematurely stop the intervention. Except for one interviewed person, all experienced weeks with bad health conditions when they could not train during the year.

Twelve healthcare professionals coached between 2 and 17 patients with COPD in the HOMEX-1 RCT. Their age range was between 25 and 53 years. Ten coaches were women (84%), and five coaches also held the function of the study nurse within the trial. Their professional backgrounds were physiotherapist (*n* = 9), sports scientist (*n* = 2), and study nurse (*n* = 1). All the 12 coaches filled in the written questionnaire. We conducted interviews with seven coaches; two coached at least five patients and five had additional supervising roles in their clinics and for the trial. We interviewed six stakeholders, three from the participating rehabilitation clinics (two physiotherapists and one medical doctor) and three from other settings (one physiotherapist, one public health specialist, and one program coordinator). Due to his function in the clinic, one person was interviewed as a coach and as a stakeholder.

### Delivery

#### Dose

The coaches conducted the three planned home visits for 52 out of the 53 intervention group participants, as one participant did not wish to receive home visits. On average, they performed 16.3 (SD 3.0; range 5–22) phone calls. Their average workload for the whole intervention was 833 min/participant (SD 245.6, range 262–1,675), divided as follows: Home visits: 277 min (92.3 min/visit), calls: 208 min (16.5 min/call), travel to home visits: 265.5 min (88.5 min/visit), and administration work: 82.2 mi. All the 53 participants received three elastic bands with varying resistance levels, exercise cards, and the training book. Upon request, six participants received an additional elastic band with a higher resistance level, and one participant received an electronic file to report the training in addition to the training book.

#### Reach

The qualitative analyses of the interviews with the nine patients revealed that their main reasons for participation were to improve their health, particularly their strength, and that experienced changes and improvements during PR triggered their decision. The majority of the persons had expectations regarding the specific HOMEX exercises and assumed them to be similar to the ones learned during PR. Others had no expectations. The opportunity to continue training with additional supervision by a professional was an important motivation to take part in the study. Usually, patients are discharged from inpatient or outpatient PR with an exercise plan but without supervision (Table 2 summarizes the derived subcategories and examples of quotes).

**Table 2:** Results from interviews of patients: Thoughts before starting the training.

**Main category**	**Subcategory**	**Example quotes**
Reasons for participation	Improve health trough regular training	“*I don't like doing sport, but I knew: it is now vital, for my health. And the time is now when I must see what and how I can go on”*
	Improve strength	“*I wanted to build my muscles”*
	Perceived improvements achieved during PR	“S*ince I was in this clinic and had to exercise every day, I quickly realized that it was really good for me. And that is why I immediately agreed to try the HOMEX training*”
Expectations	Similar exercises as known from PR	“*During the rehabilitation in X institution I did many of the exercises, so I imagined it that way”*
	No expectations	“*The expectation was probably more on myself. Whether I could do it. Would I even be able to bring such a discipline to do it?”*
	To be supervised	“*I didn't have too big expectations. But then I thought, it would be great to be supervised”*

#### Fidelity

The coaches delivered the three home visits with one exception as intended and according to protocol. The one patient who did not receive any home visit did not wish to receive one. Likewise, some patients also did not want to receive all 17 phone calls. All materials (resistance bands, exercise cards, and books) were provided according to protocol.

#### Adherence to the Intervention

From the side of the patients, adherence to the intervention was generally high. We excluded the person who did not receive any home visit and did not start with the training from the adherence analyses. On average, the 52 intervention group participants who completed the T4 assessment were adherent for 37.8 weeks (72.6%, SD 14.3 weeks) and experienced health-related training disruptions during 6.6 weeks (12.7%, SD 5.4 weeks). In weeks the participants trained, they completed on an average of 16 exercises in total, which sums up to ~80 min of exercise training per week, excluding the warm-up, and stretching exercises.

**Figure 3:**
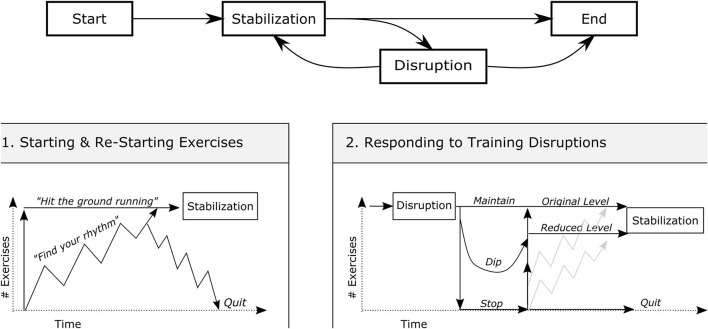
Two phases of exercise behaviors of participants: Starting training and stabilizing (1) and managing training disruptions (2).

The grounded visualization analysis suggested that the exercise behaviors of participants could be separated into phases: “Starting (and restarting)” training, “stabilizing” into a regular training routine, and “managing disruptions” to training (Figure 3). When starting the HOMEX program, participants either “hit the ground running,” starting exercise and maintaining a high level of training from the outset (*n* = 20), or take a few weeks to “find their rhythm” (*n* = 31), exhibiting increased variability in training practices during the first 2 months of the program. Most participants reached the point of “stabilization,” in which exercise behaviors leveled off and became consistent for months at a time. If training of the participants did not stabilize within the first 2 months of the program, they were more likely to quit the program prematurely (OR [95% CI]: 11.46 [1.88–91.48]). Participants who stabilized generally either maintained a training routine until the end of the program or exhibited a sudden, complete discontinuation of their training for health or personal reasons.

Participants regularly experienced potential disruptions to their training, including illnesses, COPD exacerbations, medical procedures, and vacations. Participants either “maintained” their training level despite the disruption (13 observations), “experienced a dip” in their weekly number of exercises (51 observations), or “stopped their exercises” altogether (36 observations). Restarting exercise following a disruption followed similar patterns to those exhibited at the onset of the program. Occasionally, disruptions resulted in complete, abrupt discontinuation of exercise (*n* = 6). Participants who were able to restabilize their training either did so at their “original training level” (58 observations) or at a “reduced or adapted training level” (18 observations). Reduced or adapted levels typically followed serious illnesses or medical procedures, especially those that limited the function of one part of the body and precluded certain types of exercise. Detailed visualizations of the contextualized annual training of the participants are provided in the Supplementary Material.

### Acceptability and Appropriateness

#### Patients

The results of the satisfaction questionnaire filled-in by all intervention group participants at the end of the RCT (*n* = 53) showed that they rated as most crucial feature of the program (scale 0–10; from negative to positive, mean, SD) that the exercises were presented on cards and not electronically by DVD or video (9.2, SD 1.7). This was followed by the issues that the training was home-based (9.0, SD 1.7), that the exercises were adaptable to their current health state (8.5, SD 1.7), and that only minimal equipment was required (8.2, SD 1.9). The participants rated the home visits (9.3, SD 1.2), the phone calls by the coach (9.2, SD 1.1) and the exercise cards (9.1, SD 1.5) as most helpful elements to support them to train, and as less important setting monthly (6.5, SD 2.7) and weekly (6.6, SD 2.6) goals and contacts with the sparring partner (7.0, SD 2.9).

The interviews with the nine adherent patients following the study end focused on insights into their long-term motivation, perception of the overall structure of HOMEX and specific elements, and to learn about facilitators, barriers, and suggestions, for potential implementation.

##### Long-Term Motivation to Train

Although the interviewed participants had trained until the end of the study, all had experienced difficulties or obstacles during the year. Most participants struggled sometimes to find the needed energy for starting to exercise. They felt generally weak, even after PR, making it difficult for them to find the motivation to train. Some participants had problems motivating themselves because they felt sad or depressed. For others, motivation was negatively affected by health problems such as exacerbations, surgeries, or back pain. The most frequently-experienced facilitator for long-term motivation to train was a perceived improvement in strength and health. Increasing strength, and therefore mobility and independence, was a very strong incentive. These improvements resulted in a feeling of better general health. For some participants, supervision by the coach provided them with a feeling of security and support, which facilitated adherence. Another important factor was the integration of the training in daily routine. Participants found it easier to stay motivated if they could successfully integrate the exercises into their activities of daily living. Finally, the possibility to train at home and not having to go outside or to subscribe to a gym was also a facilitator and became even more meaningful during the COVID-19 pandemic when the gyms and health offices were closed. For many patients with COPD, the protection against infection with the coronavirus was essential during this time (Table 3).

**Table 3:** Results from interviews of patients: Long-term motivation.

**Main category**	**Subcategory**	**Example quotes**
Barriers for long-term motivation	Weakness and lack of energy	“*Difficult, somehow not only for the exercises, but also in everyday life in general. It takes a tremendous amount of energy for me to just get dressed or take a shower”*
	Feelings of sadness	“*You know sometimes there is a life situation where you are a little less motivated and such. I've been a little, almost a little bit depressed”*
	Health issues	“*Unfortunately, my health has just got worse due to a severe pneumonia that I had in between, that has my health and the training severely impaired”*
Facilitators for long-term motivation	Improvements in strength and general health	“*With the exercises I noticed myself that I was getting stronger again and that I was also doing better physically”*
	Supervision by the coach	“*Because the coach is available and was always very interested, it wasn't just about doing all the exercises, but how are you, how do you feel and so, I found that very important”*
	Integration of the training in the daily routine	“*It helps a little to make the daily structure, it's like something of the day is missing if I don't do my exercises”*
	Training at home	“*Just like now with the Corona problem, of course I am here at home, but I can do my exercises independently”*

##### Conduct of the HOMEX Training: Overall Structure and Exercises

The flexibility of the training location was not only important for the long-term motivation but also one of the most appreciated features of the HOMEX training. The participants trained not only at home and inside, but also in hotel rooms, in hospitals, or outside on their balconies or in parks. Another highly appreciated feature was that the training required very little equipment. HOMEX is structured like an everyday training with a break on Sundays. For others, the short duration (15–30 min) facilitated training since it was not perceived as too time-consuming. The missing group component was a perceived barrier of HOMEX. Facilitating factors regarding the exercises were that they were easy to learn and adaptable; the majority of the interviewed patients found it very helpful that they could modify the exercises according to their current health status. On the other hand, the perception that the exercises were too easy was also a barrier. Less frequently, exercises were perceived to be as too strenuous.

As suggestions to adapt the structure and exercises of the HOMEX program for future implementation, the vast majority of the participants proposed to start the training in the clinic during PR. Some suggested having group sessions or at least to start the training together with other participants to encourage exchange. Others would have liked to receive new exercises and regular updates on HOMEX. Those participants who assessed the exercises as too easy would have found it helpful to get more difficult options. A few participants specifically asked for endurance and breathing exercises (Table 4).

**Table 4:** Results from interviews of patients: Overall structure and features of the HOMEX program.

**Main category**	**Subcategory**	**Example**
Barriers of the HOMEX structure	Training alone, not in a group	“*If there is a group, you do things together and have fun. And that is what I need, the common thing. I don't really like to exercise alone”*
Facilitators of the HOMEX structure	Training is possible everywhere	“*You are free to decide whether you want to go outside, you can also do your training in the open countryside and get some fresh air. Go to the forest or whatever, where do I use a tree as a support and that is it. It's up to everyone, you can choose freely”*
	Few equipment required	“*That you do not have to buy equipment and you can, with only the resistance band that you have, you can do all the exercises. I think that is great”*
	Daily training	“*I can exercise a little every day, all areas of the body and the same twice a week”*
	Short training	“*I dragged everything out a bit, made it longer and even so was that quickly, it took 20 to 30 minutes”*
Potential adaptations of the HOMEX structure for future implementation	Start instruction during PR	“*If you are already in the rehab, you can receive the instruction of the exercises there, at least the first exercises”*
	Provide HOMEX training in a group	“*In the group there is always a bit of motivation among each other. I would find it better, at least to start within a group”*
	Provide new exercises and HOMEX updates	“*It would be good if I had a few new exercises. That might be more interesting, again”*
Barriers of the exercises	Too easy	“*So yes, a little too easy, yeah. Yes. So now I would like to do a little more difficult exercise”*
	Too strenuous	“*The push-ups. I really did not make it till the end. Too bad, I would have liked to have made it”*
Facilitator of the exercises	Adaptable	“*You also had the choice, between the easy and between the more difficult exercises. Simply depending on how fit you were”*
	Easy to learn and execute	“*It is really very, very easy to execute. I also find for all ages. Young or old, it does not matter. It's not something so difficult that only young agile people can do it, but really for everyone”*
Potential adaptations of the exercises for future implementation	Add more strenuous exercises	“*If you have trained for a while, then you already have the feeling that you can do a little more. So yes, maybe that you might still have an option for harder exercises”*
	Add endurance and breath exercises	“*I need breathing exercises specifically for the lungs, which I missed here. There are no breathing exercises in it nor endurance”*

##### Supervision by Coach and Training Material

The participants had only positive experiences with the coach's support. For most of them, their coach was important to modify the conduct of the exercises, to instruct them how to correctly execute the exercises when they were unsure, and to answer questions. They perceived the three home visits as appropriate. For most, the phone calls were motivating and informative but the 17 pre-arranged calls were too many or not always needed. For the future, they suggested not offering pre-arranged calls but instead the possibility to contact the coach from their initiative.

Regarding the training material, they highly appreciated the training cards and judged the description and the illustration as most important which allowed them to compare themselves with the picture and to check whether they were executing the exercises correctly. They had no suggestions or ideas for changes. The training book was perceived more ambiguously. Many participants found the reports of the daily training and weekly goals and rewards bothersome over the course of the year and more useful for the study than for themselves. However, for many patients, the training book was helpful to organize and structure the training week. For future changes, most participants suggested simplifying the training book and dropping the weekly goals and rewards (Table 5).

**Table 5:** Results from interviews of patients: Supervision by coach and training material.

**Main category**	**Subcategory**	**Example**
Facilitators of the coach supervision	Correcting the conduct of exercises	“*When I wanted to start the exercise with the arms, the therapist showed me how I had to conduct “boxing” more precisely, for example how I had to rotate more my arm”*
	Instruction of exercises	“*They will show and instruct you the exercises personally”*
	Answering questions	“*Important when I had question, she could always help and clarified them”*
Barriers of the material	Goals and rewards in the training book	“*This training book thing is a bit childish. Of course you can write something down as goal or reward, it just got tedious over time.”*
	Training book in general	“*I only kept the training book for the study and not for myself”*
Facilitators of the material	Description and pictures on the cards	“*The exercises there are well described. I really don't want anything. According to the descriptions I can definitely do it 100% correctly”*
	Training book to structure and organize the week	“*I could write and prepare what I want to do more next week, it helped with the structure.”*
Potential adaptations for future implementation	Phone calls on patients' initiative	“*I think I should decide when I need to contact someone, if I have questions or feel unmotivated”*
	Simplify/reduce the training book	“*Just that the training book could be a little simpler, just less”*

#### Coaches

The questionnaire results (scales 0–10, from negative to positive, median and interquartile range) revealed that the coaches' overall impression of the program was very positive (8, IQR 7.5–9) and that they assessed HOMEX to be an efficient strength training program for patients with COPD (8, IQR 7.5–9). As most relevant elements for the effectiveness of the program, they rated the personal coach (10, IQR 9–10), the exercise cards (9.5, IQR 9–10), and the home visits (9.5, IQR 8–10), followed by the calls (8, IQR 7.5–9.5), the training book (8, IQR 6.5–10) and the monthly and annual goals (8, IQR 7–9.5). The relevance of the sparring partner was very differently perceived among the coaches (6, IQR 5–8). The setting of weekly goals was regarded as less important or even annoying or disruptive (4.5, IQR 4–6.5). Almost all coaches liked to work as a coach (8, IQR 6–9), the more patients they supported the better they liked the work overall.

The interviews with coaches showed that they perceived HOMEX to be suitable as a follow-up program to PR in order to maintain or build upon training gains. As major advantages, they described that the program is individually adaptable, that it bridges the end of the rehabilitation with daily life at home, that the exercises are simple and embedded in a coaching program, and that the program has a long-term character. Overall coaches faced a need to provide patients with home-based and individually adaptable exercises after PR completion. They specifically highlighted the importance of the first home visit and the necessity to invest enough time and effort, in order to build up the personal relationship with the patients and to learn about the concrete environment, which were both essential for the further course of the coaching. As limitations, they experienced the missing endurance and breathing parts. Summarized, the coaches perceived that HOMEX closes a gap in the Swiss health system.

We gathered further profound results regarding the coaches' general role, facilitators, and barriers of their everyday work, insight into how to cope with training disruption, their training and coach materials as well as the specific program elements and target population, not described here. However, based on all the coaches' experiences, we redesigned and reworded the exercise cards, introduced three new exercises, and refined the training book. In addition, this input informed the adaptation of the coaches' education and training manual and program.

#### Stakeholders

All three stakeholders from the rehabilitation clinics valued HOMEX very highly, particularly because it is adaptable in terms of volume and intensity, offers a unique way for training at home for patients who cannot leave the house, which was accentuated in times of the Covid-19 pandemic, and because the patients liked it. They rated the home visits as an important part of the program to support patients in implementing the concrete exercise in their usual environment. They perceived opportunities to supplement the HOMEX exercise program with PR elements such as self-management strategies to cope with symptoms, self-medication, breathing techniques, or exacerbation education. At the same time, the personnel cost and time required for the home visits, the fact that these costs are currently not refunded in a standardized way in the PR setting by health insurances, and that the inpatient clinics do not have a mandate to treat patients at home were reasons why HOMEX could not be offered as a complete program in the clinics after the RCT completion. Currently, the physiotherapists involved in the trial instruct single HOMEX exercises and offer the video-based my HOMEX program to patients for their home training after discharge (the video-based version of HOMEX, www.myhomex.ch, was developed after the onset of the Corona pandemic in spring 2020 when people were advised to stay at home whenever possible). In summary, the relevant persons from the PR clinics perceived HOMEX as an optimal supplement of their current offers, meeting the needs of a part of the patients. Suggestions to improve sustainability in the long-term were to reduce the number of home visits and calls, to introduce the web-based myhomex version during the inpatient time in the clinic, to start negotiations with health insurances for reimbursement, and to learn more about which patients adhered to and benefitted from the program. The results of the interviews with three additional health professionals who work in institution, which are potential new providers of HOMEX, were used to further develop the implementation of the program in their setting, and the detailed results are not described here.

## Discussion

This mixed-methods study showed very high acceptability of the HOMEX program by patients, coaches, and stakeholders. The home-based aspect of HOMEX was perceived as a particular strength, and some interaction with other patients emerged as future need. Furthermore, patients, coaches, and stakeholders perceived the exercises as appropriate, the strength program as efficient for people with COPD, and as relevant for rehabilitation to build a bridge of the experienced gap between the improvements after PR and the next exacerbation.

### Key Findings

A remarkably high proportion of participants managed to conduct the exercises regularly over the year, consistent with other resistance-based training programs in COPD (18). Though the issue of adherence is complex and multifactorial, it is usually reported only as an aggregated value over the entire study (19). Detailed compliance patterns are rarely studied over the course of RCTs. Unsurprisingly, the majority of this severely ill and comorbid patient group experienced regular health and personal issues that were the main causes for training disruptions. We identified two patterns of the exercise behaviors of the participants. The first was that those who succeeded in stabilizing their weekly number of exercises soon after HOMEX onset were more likely to keep on training long-term compared with those who did not stabilize their training routine within the first 2 months, which underlies the relevance of building in the early phase (20). The second pattern was that after the frequently experienced disruptions, participants either maintained their training level or, often after serious illnesses or medical problems, reduced the number of exercises or stopped training. In contrast to RCTs of other exercise interventions, we did not observe a slow or sporadic decrease in adherence over time (21). Although it appeared that coaching calls did not generally coincide with restarting after a period of non-compliance, our sample was not large enough to examine the patterns in a more detailed way, and contextual information around disruptions was missing. However, we can derive from the detected patterns that coaches should particularly focus on starting the training, and after disruptions to support the patients to quickly find a personal exercise level and to rapidly stabilize the training routine again, respectively.

A key issue for the motivation of participants to start the program in the first place and to train in the long-term was the perception of concrete improvements due to the training, which was also shown previously (22). This was crucial to their training adherence. Based on this finding, the time at the end of a PR program could be used as a window of opportunity to motivate patients for subsequent training in the long term, because patients usually are just experiencing positive changes. The other important aspects for the long-term motivation of patients to train were all core features of the HOMEX program, namely the supervision by the coach so that the training was integrated into daily routine and that it was home-based. The latter two aspects highlight again the relevance that the training becomes a habit in daily routine, which was one of our main requirements for the program when we developed it (20). On the other side, the flexibility of the program regarding training location allowed participants to train not only in their usual routine but also at times when participants were on holiday or in the hospital. In contrast to other studies, the role of social support received by family members or friends was less important for our group of participants (22, 23).

Coach support was not only crucial for the motivation of participants to train, it was also practically important for them to learn the exercises correctly and to get feedback. Participants highly appreciated the three home visits, and the coaches and stakeholders emphasized that home visits were the unique factor of the program and not offered by existing ones. However, the home visits were also responsible for the main part of the cost of the intervention. On average, one-third of the annual workload of the coaches for a HOMEX participant was spent on the home visits and travel to the visits. Since one crucial aspect for the sustainability of HOMEX is the cost of the program, we considered whether the number of home visits could be reduced or replaced by video calls. If the participants start with the HOMEX program following PR, they could already be instructed with the exercises during PR and prepared for the implementation at home. Support for establishing the concrete training place at home, which was a central aspect of the visits, could also be provided by the video call. However, as we learned from the patients and the involved healthcare professionals, at least one home visit is core for the program. In addition, the cost of HOMEX is still low compared with supervised center-based training.

### Implications for Research and Practice

Based on our results, we identified three main areas for further research. First, it is of high interest to evaluate the feasibility and subsequently the effectiveness of different adaptations of the program that make it less cost- and time-intensive, particularly the offer of only one home visit, the replacement of in-person visits by video calls, and the use of a hybrid delivery of the exercises using both cards and the website. The same applies to the enrichment of the program with additional elements such as an endurance part or COPD-specific self-management components. One crucial point when designing these adaptations will be not to lose the essential benefit of the home visits. Second, research is needed to better understand adherence patterns with a specific emphasis on program start and mechanisms after disruptions. These insights could be used to develop guidance for healthcare professionals on how to best support and motivate patients during these crucial times. Third, it would be very useful to learn more about patient characteristics of patients for whom home-based training is likely to be successful and for whom center-based programs are more appropriate.

One main aim of this study was to support the sustainability of HOMEX in practice. We are currently in contact with the PR clinics that participated in the trial to find ways for them to offer the program to patients after discharge from PR. The main discussion points are instruction during PR, the provision of a cheaper, self-printed version of the exercise cards, the integration of myhomex, and the feasibility of home visits. However, for the long-term sustainability of the program in the PR setting it is crucial that the reimbursement is ensured. In addition to the PR setting, we are currently implementing the HOMEX program together with the cantonal lung association (“Lunge Zürich”) in the community setting, where it will be provided to persons with COPD by a half-day-exercise training course and subsequent home visits by a trained nurse. Finally, the web-based myhomex version is promoted in collaboration with Senior Citizen's University of Zurich and the Prevention and Health Promotion Canton of Zurich to their members and senior citizens in general, respectively. The Covid-19 pandemic increased the demand for home-based programs.

### Strengths and Limitations

The strengths of this study are that we took the perspective of all involved persons into account, which provided an overall picture of the program from different angles. Of particular value is the assessment and visualization of the daily training behavior of participants during 1 year, considering also several context factors that allowed a unique insight into long-term training patterns and disruptions. Furthermore, the study allowed insights into the long-term motivation of the participants. Finally, the study team comprised persons with different professional and scientific backgrounds who integrated several methodological approaches.

One limitation of our study is the generalizability of the results. Our patient population included, with one exception, persons who were recruited during inpatient PR. It remains unclear whether persons who completed outpatient PR who are usually less severely ill would have behaved and perceived specific elements differently. Moreover, we do not know whether and how the intervention works in people who did not follow PR at all. The results of the HOMEX-2 RCT will presumably answer some of these uncertainties. In addition, we focused on the in-depth interviews of persons who did not prematurely stop the intervention, since our focus was to get insights on their long-term motivation. We consecutively asked participants from the clinics whether they agreed to the interview. This might have introduced selection bias of including more healthy people because some participants already died in the meantime, and of persons who were motivated to conduct the interview. Moreover, targeting participants who did not succeed in training in the long-term would have allowed going more deeply into the underlying mechanisms of training disruptions.

## Conclusion

With this study, we went beyond the effectiveness analyses of the HOMEX exercise program. The results provided unique insights of the involved persons, with a particular focus on the long-term training behavior of the participants and their perception and experience with the exercise program. These findings enabled us to optimize the training material, rethink the structure of program, and adapt the program for sustainable further use in clinical and other settings.

## Data Availability Statement

The raw data supporting the conclusions of this article will be made available by the authors, without undue reservation.

## Ethics Statement

The studies involving human participants were reviewed and approved by Kantonale Ethikkommission Zürich Stampfenbachstrasse 121 8090 Zürich, Switzerland. The patients/participants provided their written informed consent to participate in this study.

## Author Contributions

AF, RK, and TC developed the concept and overall design of the study. TC developed and conducted the in-depth interviews with the patients and the qualitative analyses, supported by AF as second coder. AF designed and analyzed the survey and interviews with the participants at RCT end. RK designed, conducted and analyzed the survey, and interviews with the coaches in exchange with AF and analyzed the RCT documentation. AP developed the analysis plan for the annual training data, conducted the analyses, and developed the visualization of the data. KD was the master coach and piloted together with TR the feasibility of the intervention. AF, MP, TR, and KD designed the conception of the RCT. AF, TC, and RK drafted the manuscript. All authors read, revised, provided input, and approved the final version of the manuscript.

## Funding

This study was supported by Spark of the Swiss National Science Foundation (CRSK-3_190329) and by the Velux Stiftung (Velux Foundation, 1393). The HOMEX-1 RCT was supported by the Research Fund of the Swiss Lung Association, Berne (2017-19), and by the Research Fund of Klinik Barmelweid, Switzerland. The funding bodies had no role in the design of the study, data collection, analysis and interpretation of data, and in writing the manuscript.

## Conflict of Interest

The authors declare that the research was conducted in the absence of any commercial or financial relationships that could be construed as a potential conflict of interest.

## Publisher's Note

All claims expressed in this article are solely those of the authors and do not necessarily represent those of their affiliated organizations, or those of the publisher, the editors and the reviewers. Any product that may be evaluated in this article, or claim that may be made by its manufacturer, is not guaranteed or endorsed by the publisher.
