# Supplementary material for: Evaluation of the Implementation of a Home-Based Exercise Training Program for People With COPD: A Mixed-Methods Study
Source: Front Rehabil Sci. 2021 Oct 26;2:743588. doi: 10.3389/fresc.2021.743588 (PMC9397666; doi:10.3389/fresc.2021.743588)

Supplementary material

Participant adherence visualizations

In this analysis, quantitative data included demographic characteristics of the participants and exercises reported in study diaries. Contextual and qualitative data included self-reported notes on adverse events, non-HOMEX activities, and environmental factors that affected participants' training each week. The notes collected by HOMEX coaches during check-in calls were also analyzed.

Participant dossiers detailed participants' demographic information, COPD symptoms, and self-efficacy and included a visualization which described and contextualized exercise data. Health events, pulmonary rehabilitation, and vacations were also noted as potential disruptions to training:

- **Serious adverse events (SAEs):** Health events which required hospitalization, both related and unrelated to COPD
- **Adverse events:** Patient-reported or coach-confirmed health events, such as illnesses, injuries, and accidents

Participant 1

Adherence and Baseline Demographics

**Adherence: All Weeks (%)**	42.3
**Adherence: Healthy Weeks (%)**	51.5
**Bad Health (Weeks)**	19
**Age**	61
**Sex**	Female
**FEV1 (% Pred)**	29.8
**CRQ Dyspnea**	5.4
**Marital Status**	Widowed
**Living Situation**	Lives alone
**Comorbidities (n)**	1
**Sparring Partner**	Yes
**Set 2-Month Goal**	Yes
**Set 12-Month Goal**	Yes

Self-Efficacy

How confident are you in your ability to…

	3 mo.	6mo.	12 mo.
**Practice Daily**	9	5	6
**Practice Correctly**	10	6	8
**Adjust Intensity**	10	6	5
**Keep an Agenda**	10	4	5

Notes

During the program, the participant did not regularly engage in other physical activities. She reported that she stopped HOMEX training to engage in her own exercise program.

In her interview, she reported that she noticed positive effects of training in her daily life. This positive change motivated her to continue training.

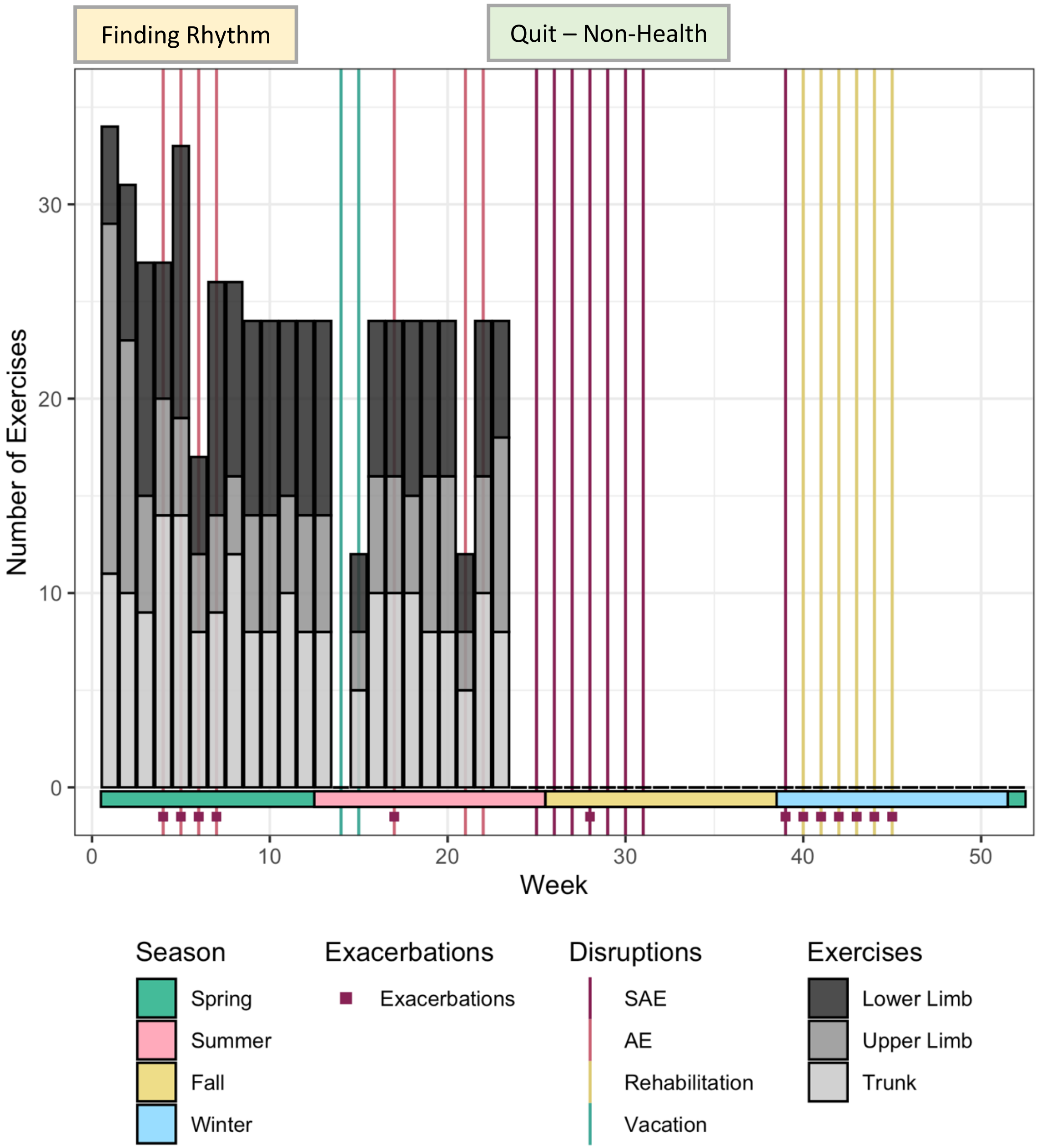

Participant 2

Adherence and Baseline Demographics

**Adherence: All Weeks (%)**	92.3
**Adherence: Healthy Weeks (%)**	97.9
**Bad Health (Weeks)**	4
**Age**	70
**Sex**	Male
**FEV1 (% Pred)**	21.8
**CRQ Dyspnea**	3
**Marital Status**	Married or partnership
**Living Situation**	Lives with partner or children
**Comorbidities (n)**	3
**Sparring Partner**	Yes
**Set 2-Month Goal**	No
**Set 12-Month Goal**	No

Self-Efficacy

How confident are you in your ability to…

	3 mo.	6mo.	12 mo.
**Practice Daily**	10	8	10
**Practice Correctly**	9	10	10
**Adjust Intensity**	9	9	10
**Keep an Agenda**	10	10	10

Notes

Participant reported experiencing breathlessness regularly throughout the program. He did not regularly engage in physical activity outside of HOMEX training.

He did not participate in the follow-up interviews.

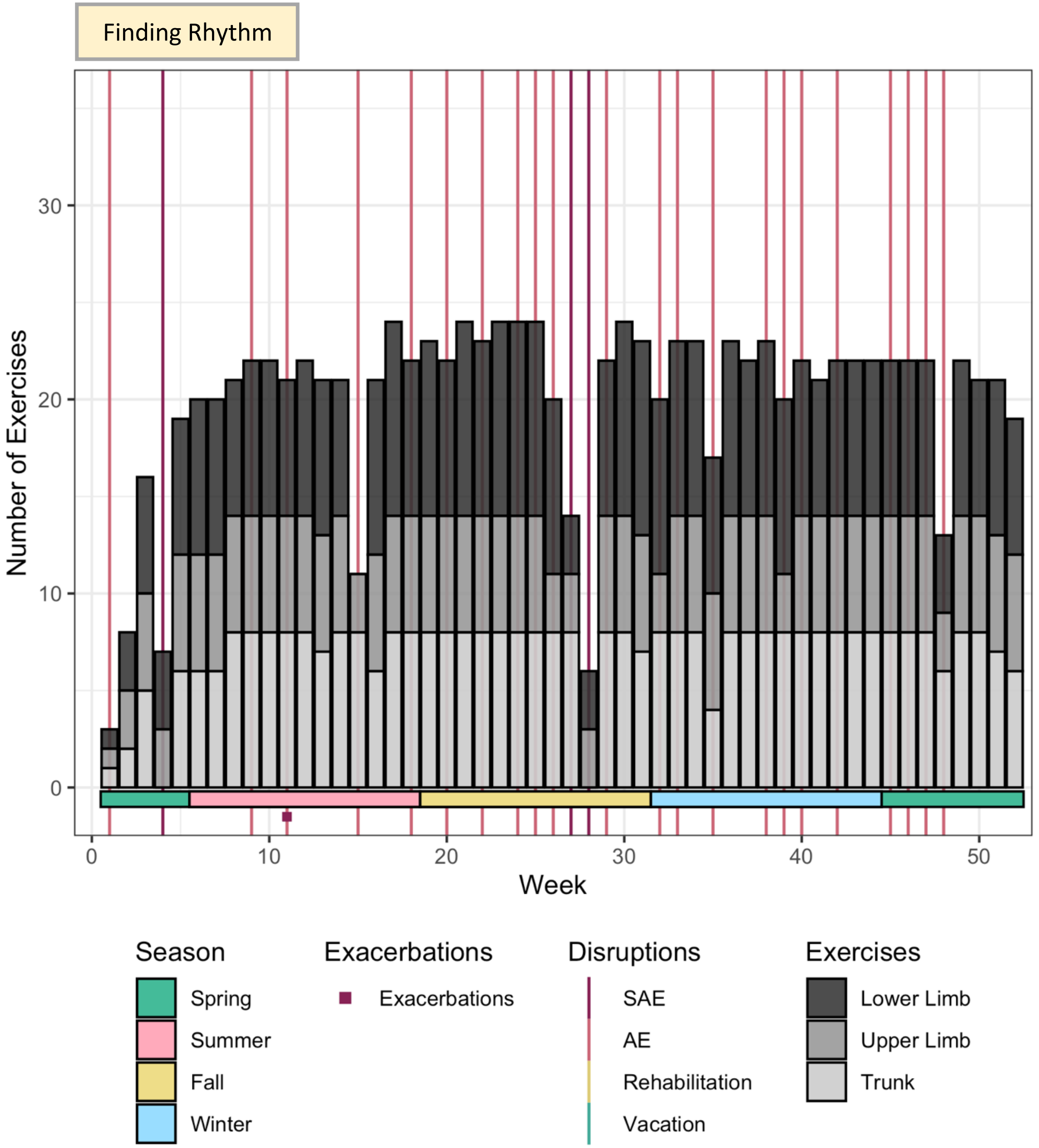

Participant 3

Adherence and Baseline Demographics

**Adherence: All Weeks (%)**	19.2
**Adherence: Healthy Weeks (%)**	20.4
**Bad Health (Weeks)**	3
**Age**	52
**Sex**	Male
**FEV1 (% Pred)**	63.7
**CRQ Dyspnea**	6.6
**Marital Status**	Married or partnership
**Living Situation**	Lives with partner or children
**Comorbidities (n)**	4
**Sparring Partner**	NR
**Set 2-Month Goal**	Yes
**Set 12-Month Goal**	Yes

Self-Efficacy

How confident are you in your ability to…

	3 mo.	6mo.	12 mo.
**Practice Daily**	10	-	1
**Practice Correctly**	10	-	10
**Adjust Intensity**	9	-	10
**Keep an Agenda**	9	-	5

Notes

The participant reported stopping HOMEX training due to low motivation. He regularly engaged in physical activity other than HOMEX throughout the year and believed that his COPD was no longer a problem.
He reported that he noticed positive effects of training in daily life and that using a coach motivated him to train.

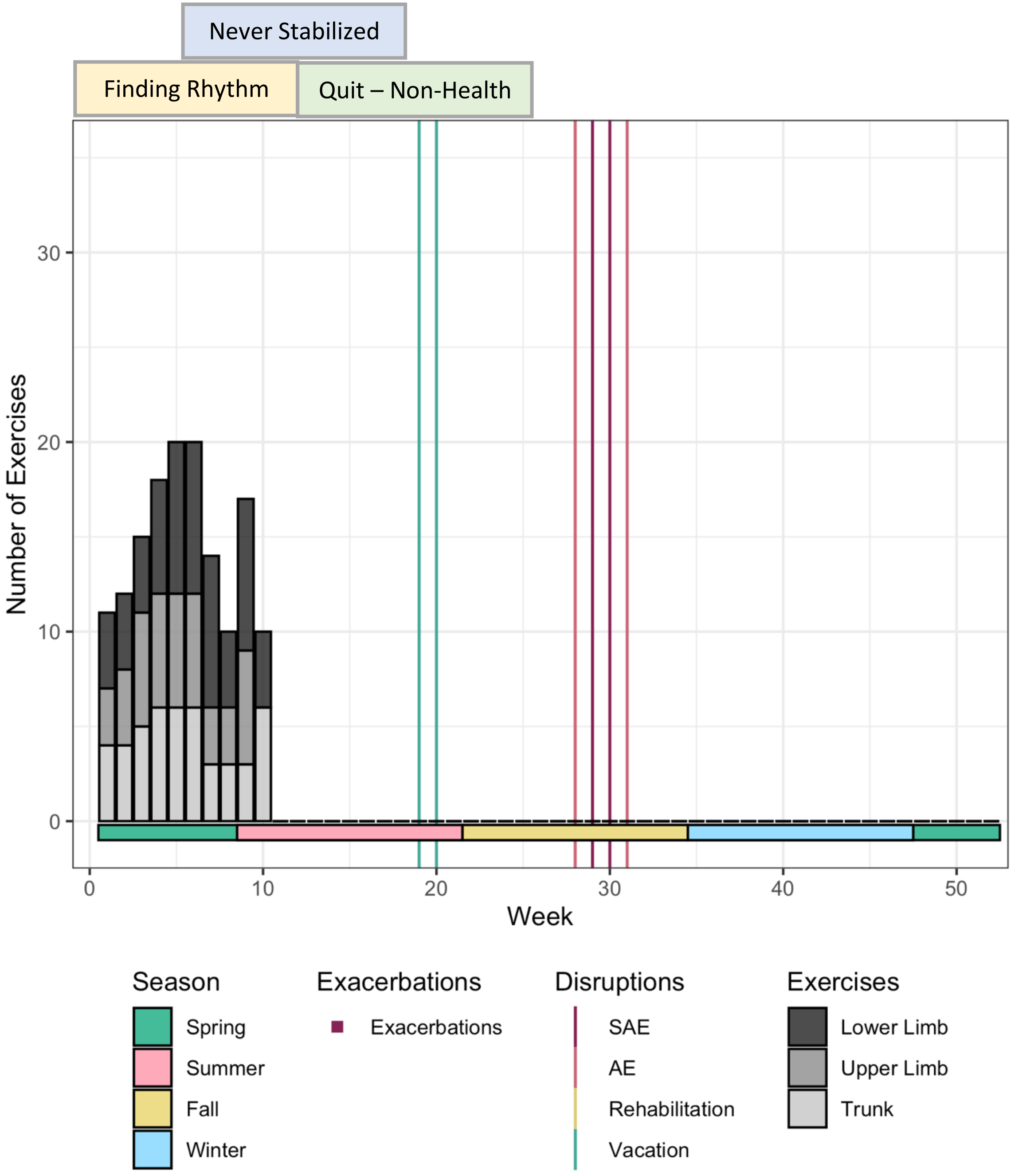

Participant 4

Adherence and Baseline Demographics

**Adherence: All Weeks (%)**	84.6
**Adherence: Healthy Weeks (%)**	95.7
**Bad Health (Weeks)**	6
**Age**	71
**Sex**	Female
**FEV1 (% Pred)**	64.7
**CRQ Dyspnea**	6.8
**Marital Status**	Married or partnership
**Living Situation**	Lives with partner or children
**Comorbidities (n)**	3
**Sparring Partner**	Yes
**Set 2-Month Goal**	Yes
**Set 12-Month Goal**	Yes

Self-Efficacy

How confident are you in your ability to…

	3 mo.	6mo.	12 mo.
**Practice Daily**	10	9	10
**Practice Correctly**	10	10	10
**Adjust Intensity**	10	10	10
**Keep an Agenda**	10	10	10

Notes

The participant often reported engaging in other physical activity on non-adherent weeks, even despite health-related disruptions. She reported having an exacerbation and infection at the end of the program.
She reported that she noticed positive effects of training in daily life and that using a coach motivated her to train.

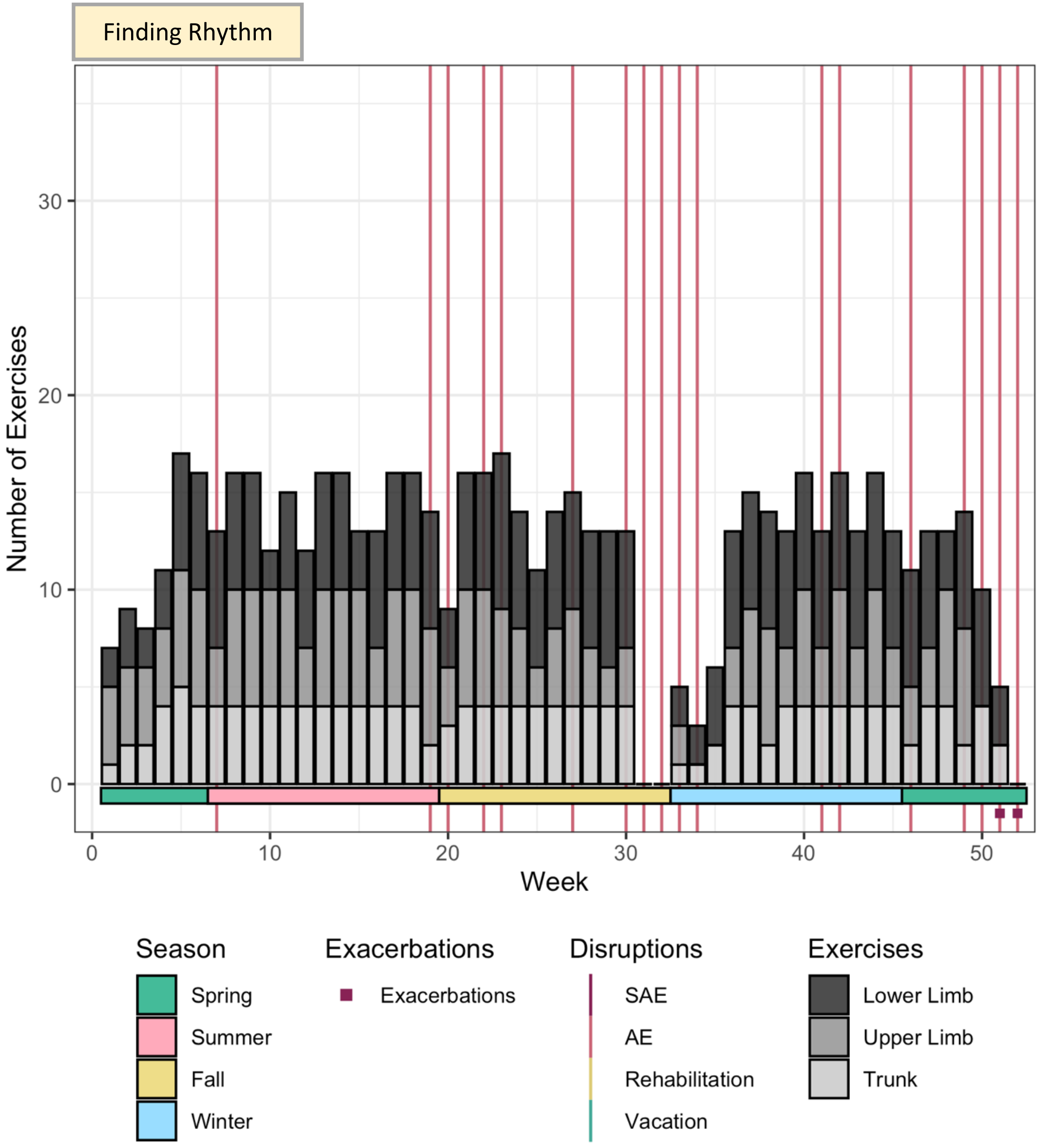

Participant 5

Adherence and Baseline Demographics

**Adherence: All Weeks (%)**	92.3
**Adherence: Healthy Weeks (%)**	94.1
**Bad Health (Weeks)**	1
**Age**	70
**Sex**	Female
**FEV1 (% Pred)**	29
**CRQ Dyspnea**	5.8
**Marital Status**	Married or partnership
**Living Situation**	Lives with partner or children
**Comorbidities (n)**	4
**Sparring Partner**	NR
**Set 2-Month Goal**	Yes
**Set 12-Month Goal**	Yes

Self-Efficacy

How confident are you in your ability to…

	3 mo.	6mo.	12 mo.
**Practice Daily**	8	8	10
**Practice Correctly**	8	8	10
**Adjust Intensity**	8	8	10
**Keep an Agenda**	10	8	8

Notes

The participant often reported engaging in other physical activity throughout the program.
She reported that she noticed positive effects of training in daily life. Experiencing these effects and her desire for health and fitness motivated her to train.

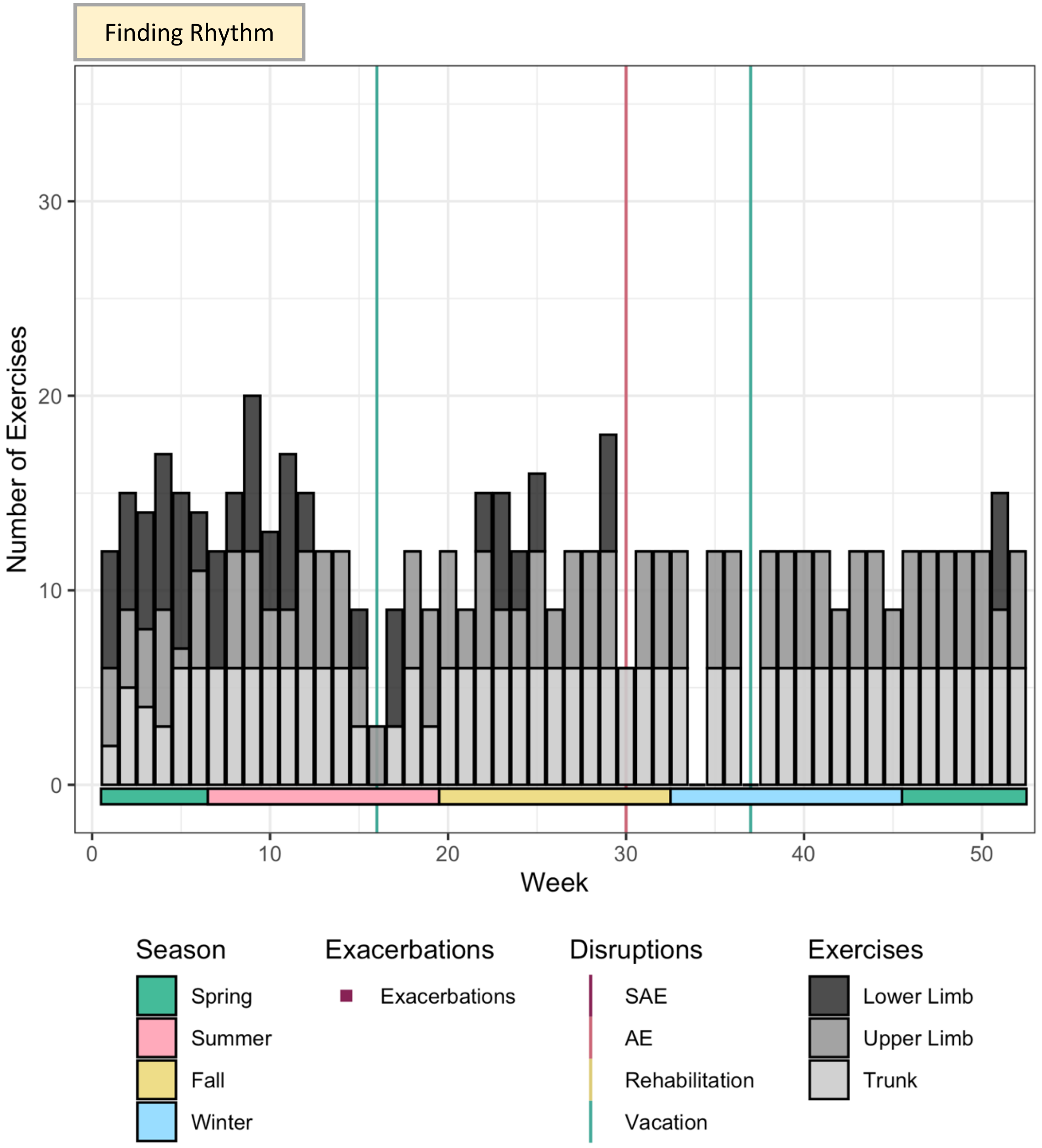

Participant 6

Adherence and Baseline Demographics

**Adherence: All Weeks (%)**	28.8
**Adherence: Healthy Weeks (%)**	28
**Bad Health (Weeks)**	2
**Age**	69
**Sex**	Female
**FEV1 (% Pred)**	43.5
**CRQ Dyspnea**	3.3
**Marital Status**	Divorced or separated
**Living Situation**	Lives alone
**Comorbidities (n)**	4
**Sparring Partner**	Yes
**Set 2-Month Goal**	Yes
**Set 12-Month Goal**	Yes

Self-Efficacy

How confident are you in your ability to…

	3 mo.	6mo.	12 mo.
**Practice Daily**	5	8	3
**Practice Correctly**	8	8	3
**Adjust Intensity**	9	8	1
**Keep an Agenda**	5	2	1

Notes

The participant did not report engaging in physical activity other than HOMEX during the program. She reported that she did not experience effects of training in daily life. She was motivated to train following rehabilitation and by her sparring partner.

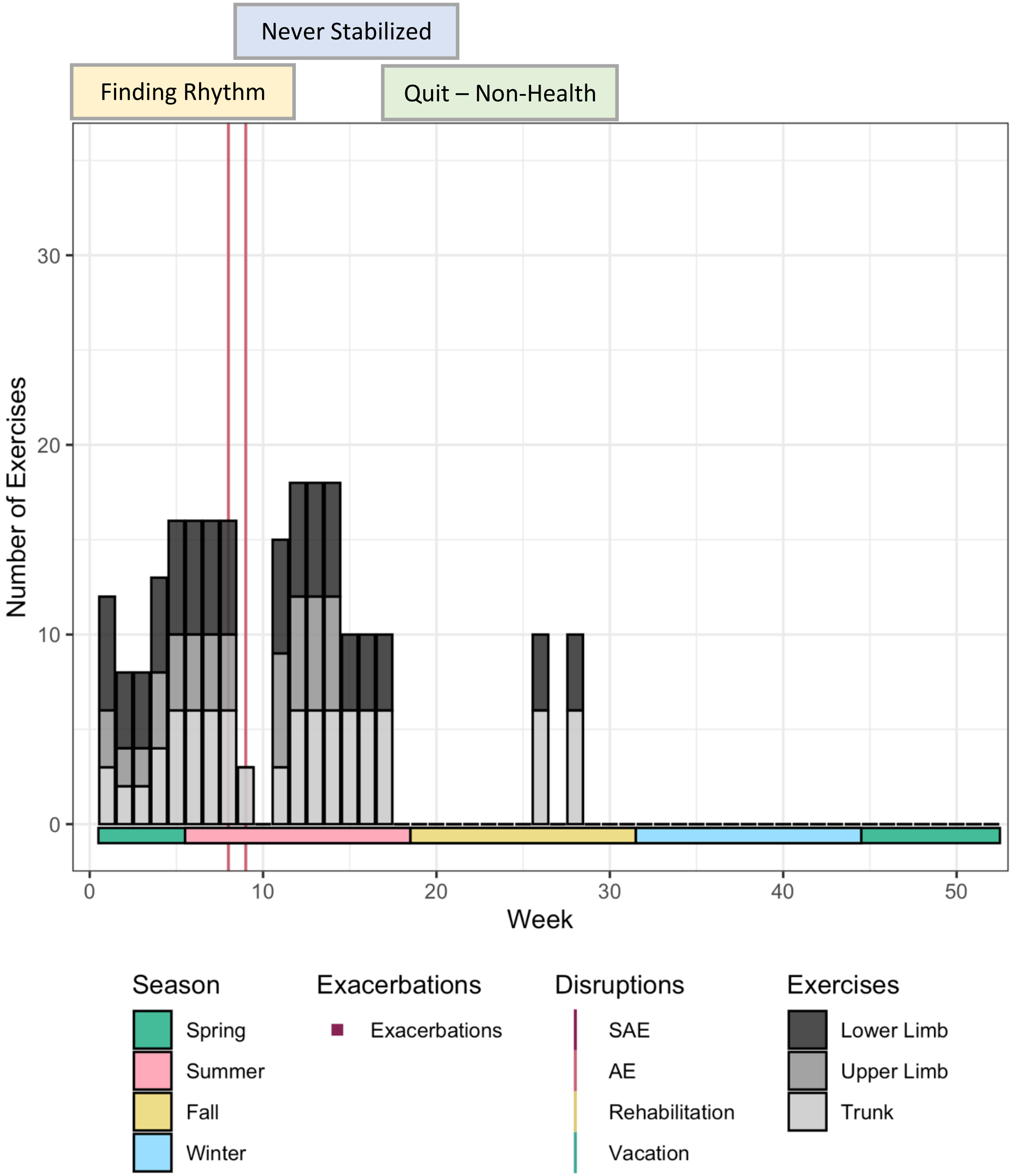

Participant 7

Adherence and Baseline Demographics

**Adherence: All Weeks (%)**	84.6
**Adherence: Healthy Weeks (%)**	87.5
**Bad Health (Weeks)**	4
**Age**	85
**Sex**	Male
**FEV1 (% Pred)**	52.4
**CRQ Dyspnea**	3.4
**Marital Status**	Married or partnership
**Living Situation**	Lives with partner or children
**Comorbidities (n)**	4
**Sparring Partner**	Yes
**Set 2-Month Goal**	No
**Set 12-Month Goal**	Yes

Self-Efficacy

How confident are you in your ability to…

	3 mo.	6mo.	12 mo.
**Practice Daily**	5	3	3
**Practice Correctly**	5	3	4
**Adjust Intensity**	5	5	4
**Keep an Agenda**	5	5	4

Notes

The participant did not report engaging in physical activity other than HOMEX during the program. He reported that he experienced positive effects of training in daily life. He was motivated to train following by his coach and partner.

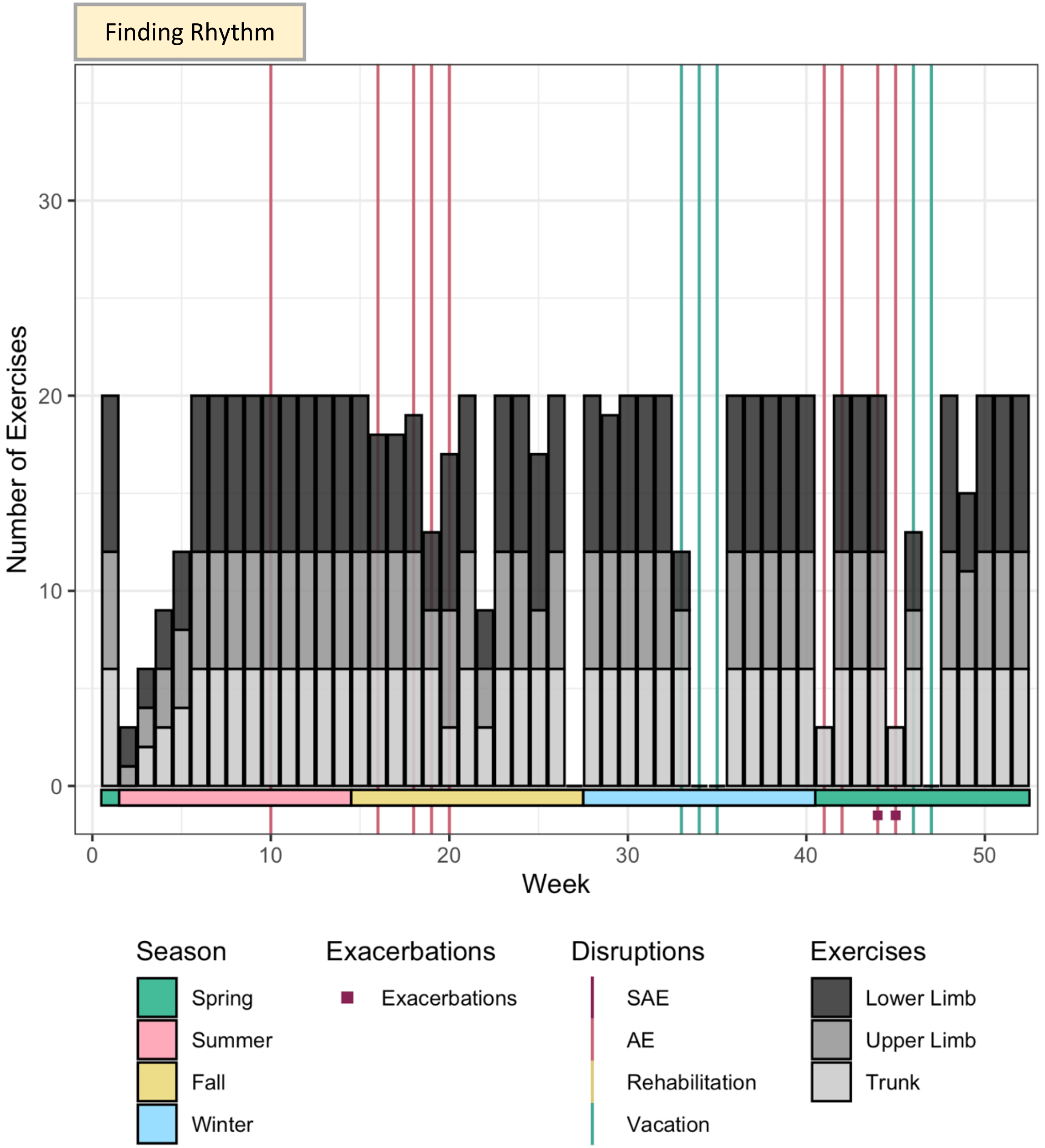

Participant 8

Adherence and Baseline Demographics

**Adherence: All Weeks (%)**	38.5
**Adherence: Healthy Weeks (%)**	43.5
**Bad Health (Weeks)**	6
**Age**	61
**Sex**	Male
**FEV1 (% Pred)**	46.4
**CRQ Dyspnea**	4.3
**Marital Status**	Divorced or separated
**Living Situation**	Lives alone
**Comorbidities (n)**	5
**Sparring Partner**	Yes
**Set 2-Month Goal**	No
**Set 12-Month Goal**	No

Self-Efficacy

How confident are you in your ability to…

	3 mo.	6mo.	12 mo.
**Practice Daily**	4	-	3
**Practice Correctly**	5	-	3
**Adjust Intensity**	5	-	10
**Keep an Agenda**	8	-	10

Notes

The participant reported stopping HOMEX training when he returned to work. He reported not enjoying structured exercise programs. However, he reported that he experienced positive effects of training.

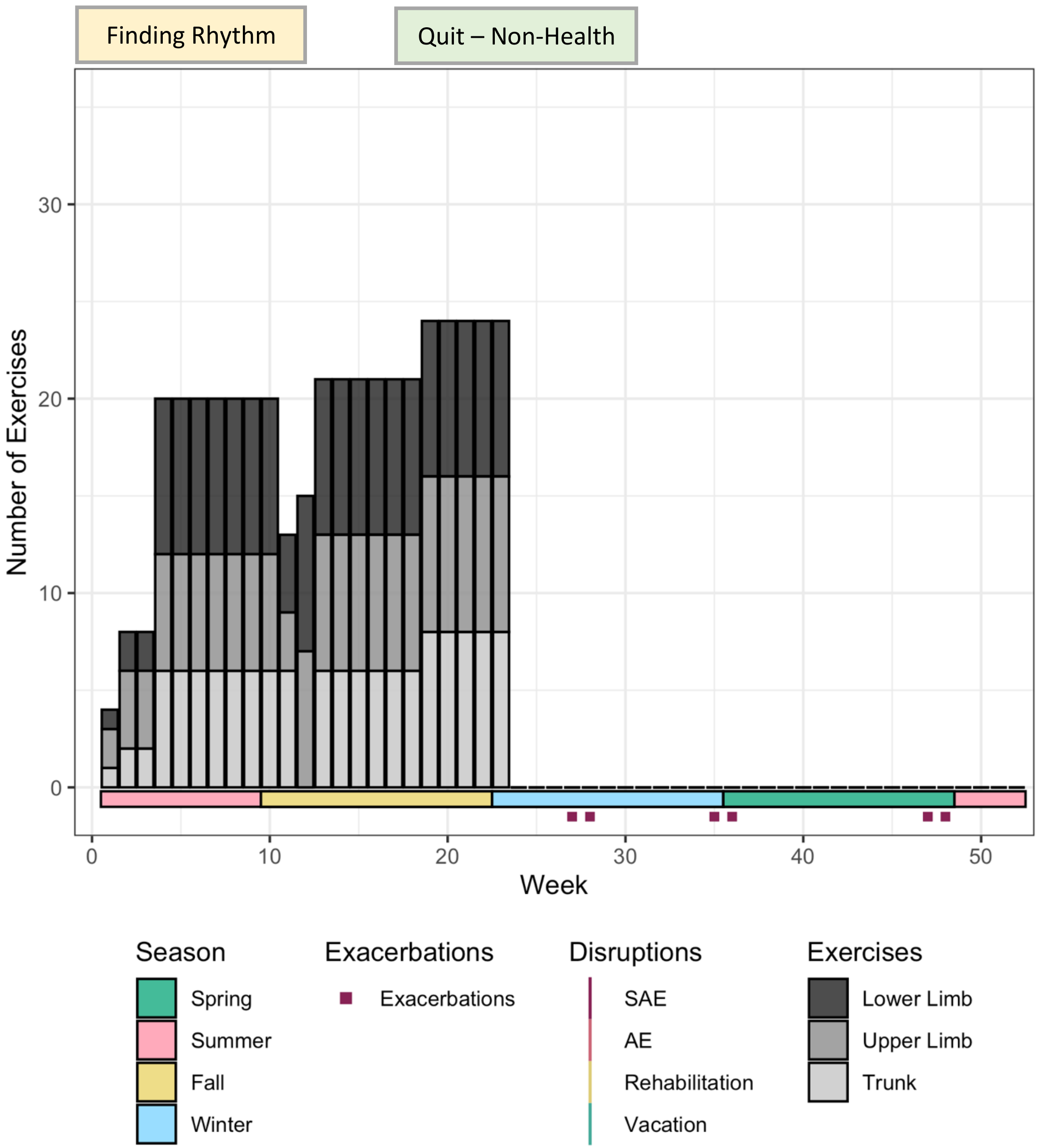

Participant 9

Adherence and Baseline Demographics

**Adherence: All Weeks (%)**	76.9
**Adherence: Healthy Weeks (%)**	84.4
**Bad Health (Weeks)**	7
**Age**	61
**Sex**	Female
**FEV1 (% Pred)**	26.8
**CRQ Dyspnea**	4.3
**Marital Status**	Widowed
**Living Situation**	Lives alone
**Comorbidities (n)**	1
**Sparring Partner**	Yes
**Set 2-Month Goal**	Yes
**Set 12-Month Goal**	Yes

Self-Efficacy

How confident are you in your ability to…

	3 mo.	6mo.	12 mo.
**Practice Daily**	9	9	8
**Practice Correctly**	8	9	8
**Adjust Intensity**	10	10	9
**Keep an Agenda**	8	9	9

Notes

The participant sometimes engaged in physical activity other than HOMEX, but this did not disrupt her training. She reported that he experienced positive effects of training in daily life.

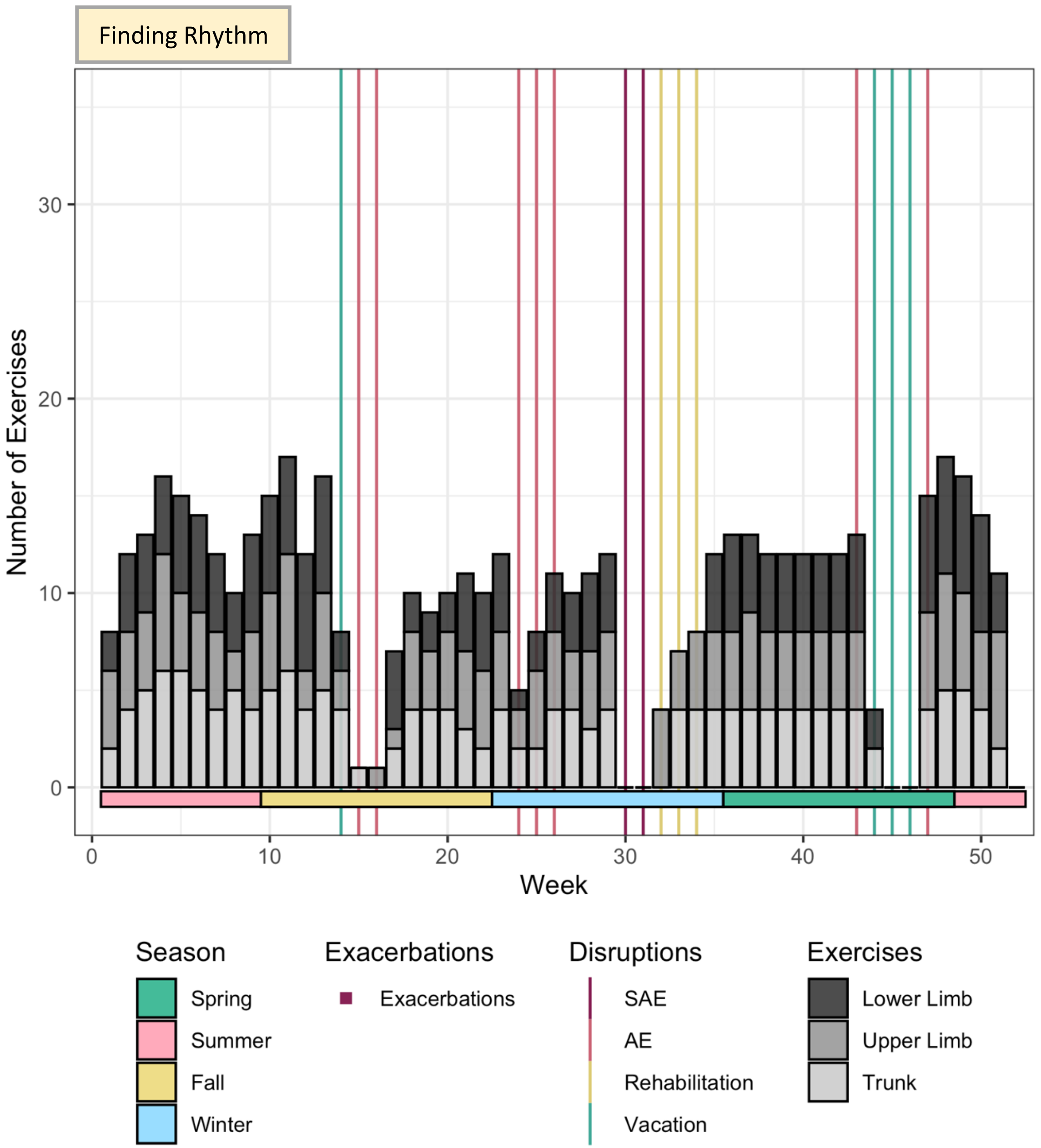

Participant 10

Adherence and Baseline Demographics

**Adherence: All Weeks (%)**	71.2
**Adherence: Healthy Weeks (%)**	78.3
**Bad Health (Weeks)**	6
**Age**	70
**Sex**	Female
**FEV1 (% Pred)**	26.2
**CRQ Dyspnea**	2
**Marital Status**	Divorced or separated
**Living Situation**	Lives with partner or children
**Comorbidities (n)**	4
**Sparring Partner**	Yes
**Set 2-Month Goal**	Yes
**Set 12-Month Goal**	Yes

Self-Efficacy

How confident are you in your ability to…

	3 mo.	6mo.	12 mo.
**Practice Daily**	9	9	10
**Practice Correctly**	9	9	10
**Adjust Intensity**	9	9	8
**Keep an Agenda**	9	9	10

Notes

The participant delayed program initiation due to illness and therefore completed less than 52 weeks of training. She underwent an operation during the program and was required to stop upper limb exercises thereafter. She reported that the experienced positive effects of training and was motivated by having a concrete goal.

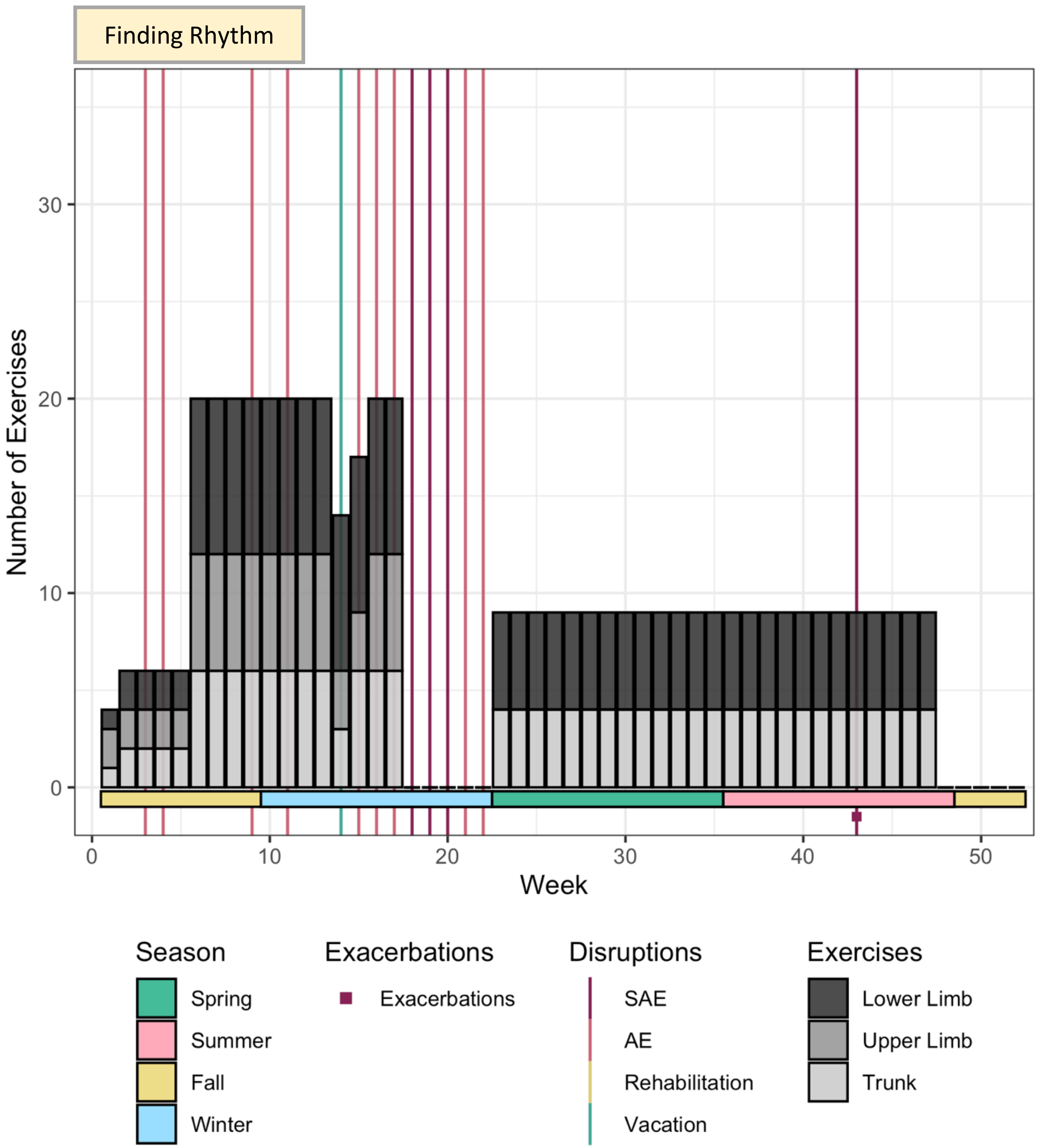

Participant 11

Adherence and Baseline Demographics

**Adherence: All Weeks (%)**	96.2
**Adherence: Healthy Weeks (%)**	98
**Bad Health (Weeks)**	2
**Age**	52
**Sex**	Female
**FEV1 (% Pred)**	44.7
**CRQ Dyspnea**	6.7
**Marital Status**	Widowed
**Living Situation**	Lives alone
**Comorbidities (n)**	6
**Sparring Partner**	Yes
**Set 2-Month Goal**	Yes
**Set 12-Month Goal**	Yes

Self-Efficacy

How confident are you in your ability to…

	3 mo.	6mo.	12 mo.
**Practice Daily**	9	9	7
**Practice Correctly**	9	8	9
**Adjust Intensity**	9	8	8
**Keep an Agenda**	9	9	8

Notes

The participant did not report engaging in physical activity other than HOMEX during the program. She had mixed feelings about whether she experienced positive effects of training in daily life. She was motivated to train because she understood the importance and was committed to the study.

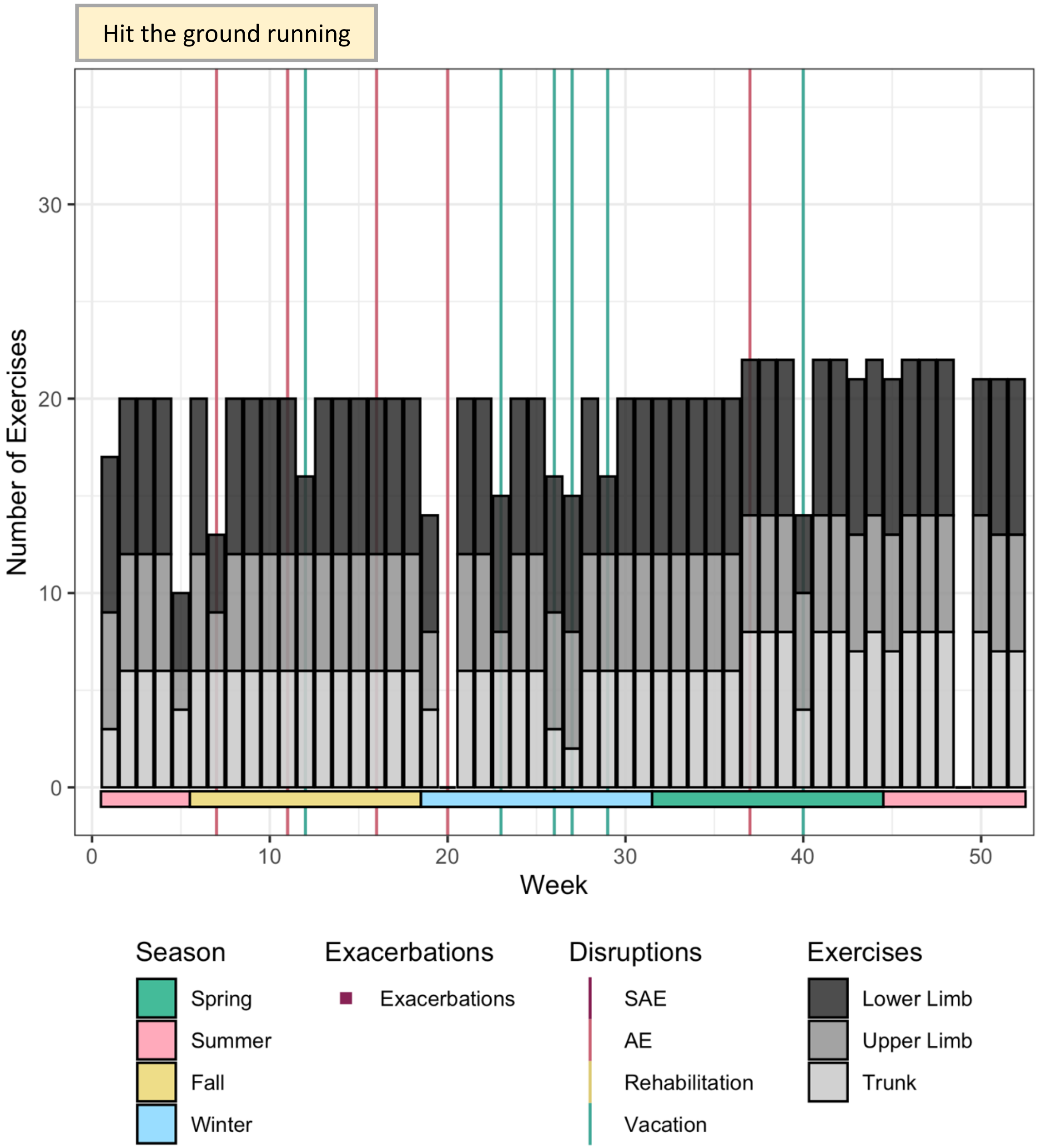

Participant 12

Adherence and Baseline Demographics

**Adherence: All Weeks (%)**	96.2
**Adherence: Healthy Weeks (%)**	100
**Bad Health (Weeks)**	3
**Age**	71
**Sex**	Female
**FEV1 (% Pred)**	49.5
**CRQ Dyspnea**	6.2
**Marital Status**	Married or partnership
**Living Situation**	Lives with partner or children
**Comorbidities (n)**	2
**Sparring Partner**	Yes
**Set 2-Month Goal**	Yes
**Set 12-Month Goal**	Yes

Self-Efficacy

How confident are you in your ability to…

	3 mo.	6mo.	12 mo.
**Practice Daily**	10	-	10
**Practice Correctly**	10	-	10
**Adjust Intensity**	10	-	10
**Keep an Agenda**	10	-	10

Notes

The participant began regularly engaging in physical activity other than HOMEX at week 20, coinciding with a drop in HOMEX training. She reported that she experienced positive effects of training in daily life and was motivated to continue training to improve her quality of life and independence.

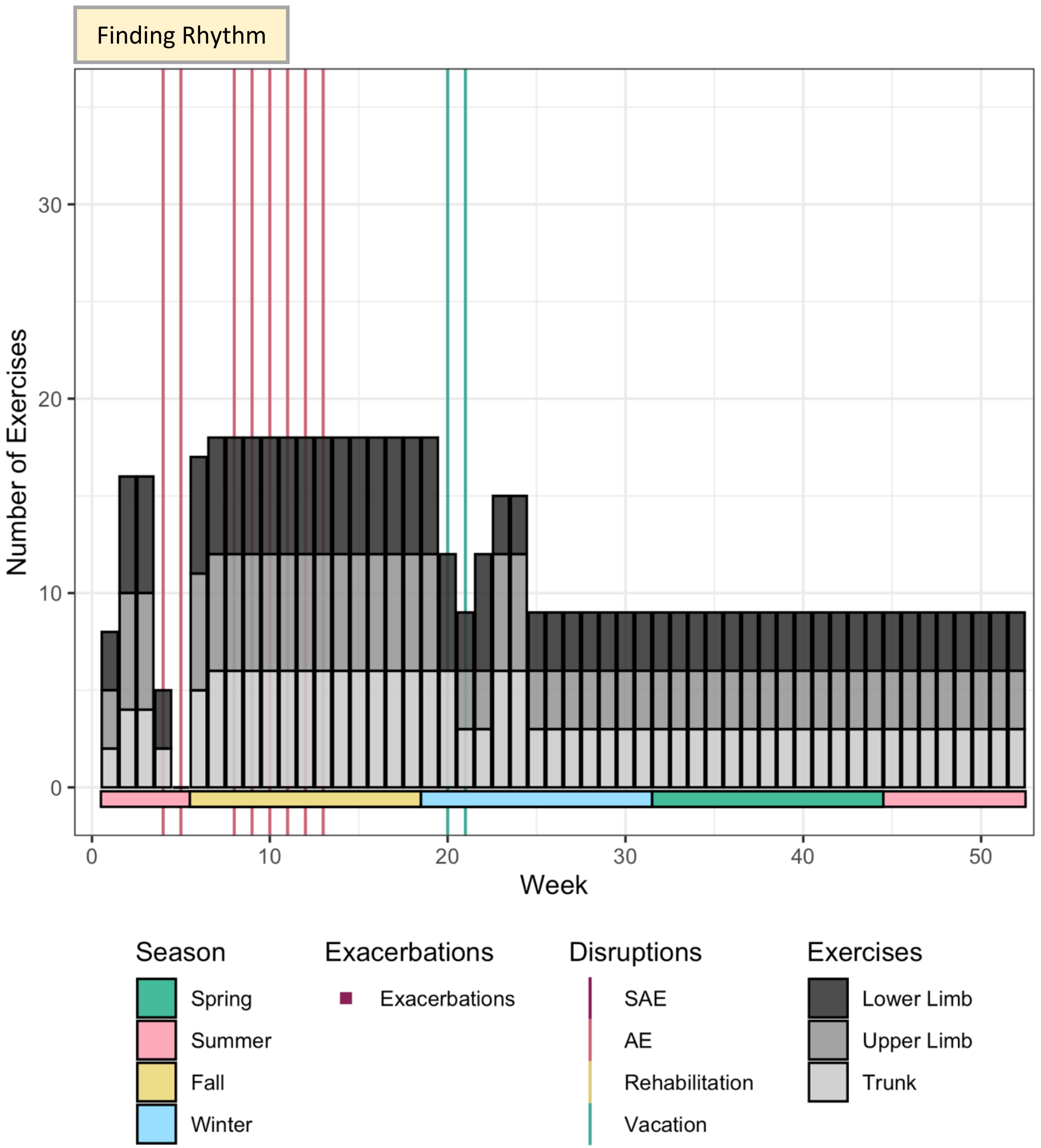

Participant 13

Adherence and Baseline Demographics

**Adherence: All Weeks (%)**	98.1
**Adherence: Healthy Weeks (%)**	98.1
**Bad Health (Weeks)**	0
**Age**	79
**Sex**	Male
**FEV1 (% Pred)**	65.6
**CRQ Dyspnea**	5.5
**Marital Status**	Single
**Living Situation**	Lives alone
**Comorbidities (n)**	5
**Sparring Partner**	Yes
**Set 2-Month Goal**	No
**Set 12-Month Goal**	No

Self-Efficacy

How confident are you in your ability to…

	3 mo.	6mo.	12 mo.
**Practice Daily**	9	9	10
**Practice Correctly**	9	9	10
**Adjust Intensity**	9	9	10
**Keep an Agenda**	9	9	10

Notes

The participant reported feeling positive effects of training in her daily life. She was motivated to continue training due to her commitment to the study.

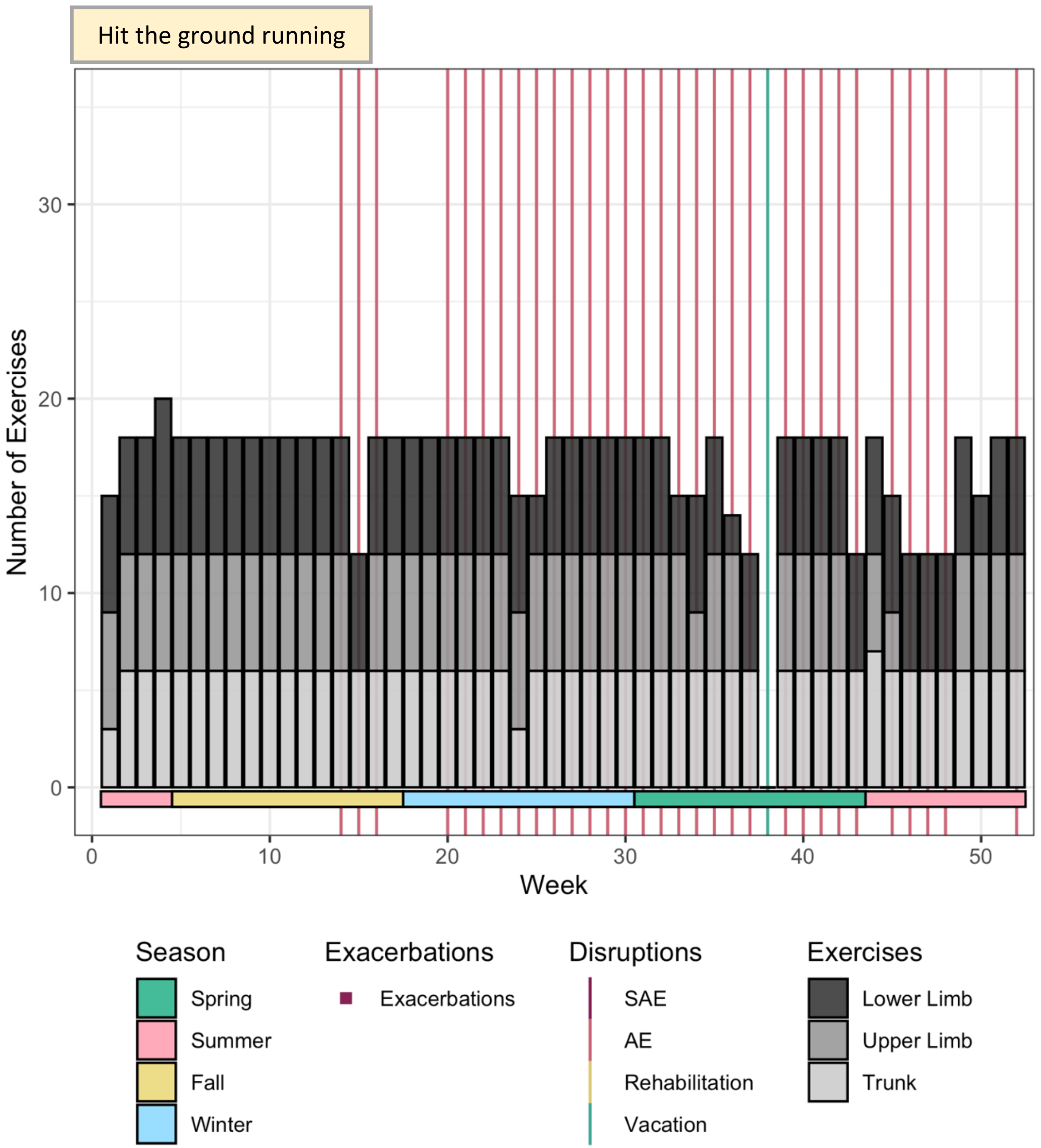

Participant 14

Adherence and Baseline Demographics

**Adherence: All Weeks (%)**	84.6
**Adherence: Healthy Weeks (%)**	100
**Bad Health (Weeks)**	10
**Age**	54
**Sex**	Male
**FEV1 (% Pred)**	70.9
**CRQ Dyspnea**	4
**Marital Status**	Divorced or separated
**Living Situation**	Lives alone
**Comorbidities (n)**	8
**Sparring Partner**	Yes
**Set 2-Month Goal**	Yes
**Set 12-Month Goal**	No

Self-Efficacy

How confident are you in your ability to…

	3 mo.	6mo.	12 mo.
**Practice Daily**	-	9	10
**Practice Correctly**	-	10	9
**Adjust Intensity**	-	9	10
**Keep an Agenda**	-	9	10

Notes

The participant did not report engaging in physical activity other than HOMEX during the program. He reported experiencing positive effects of training in his daily life and was motivated to train both by other people and to achieve greater independence and quality of life.

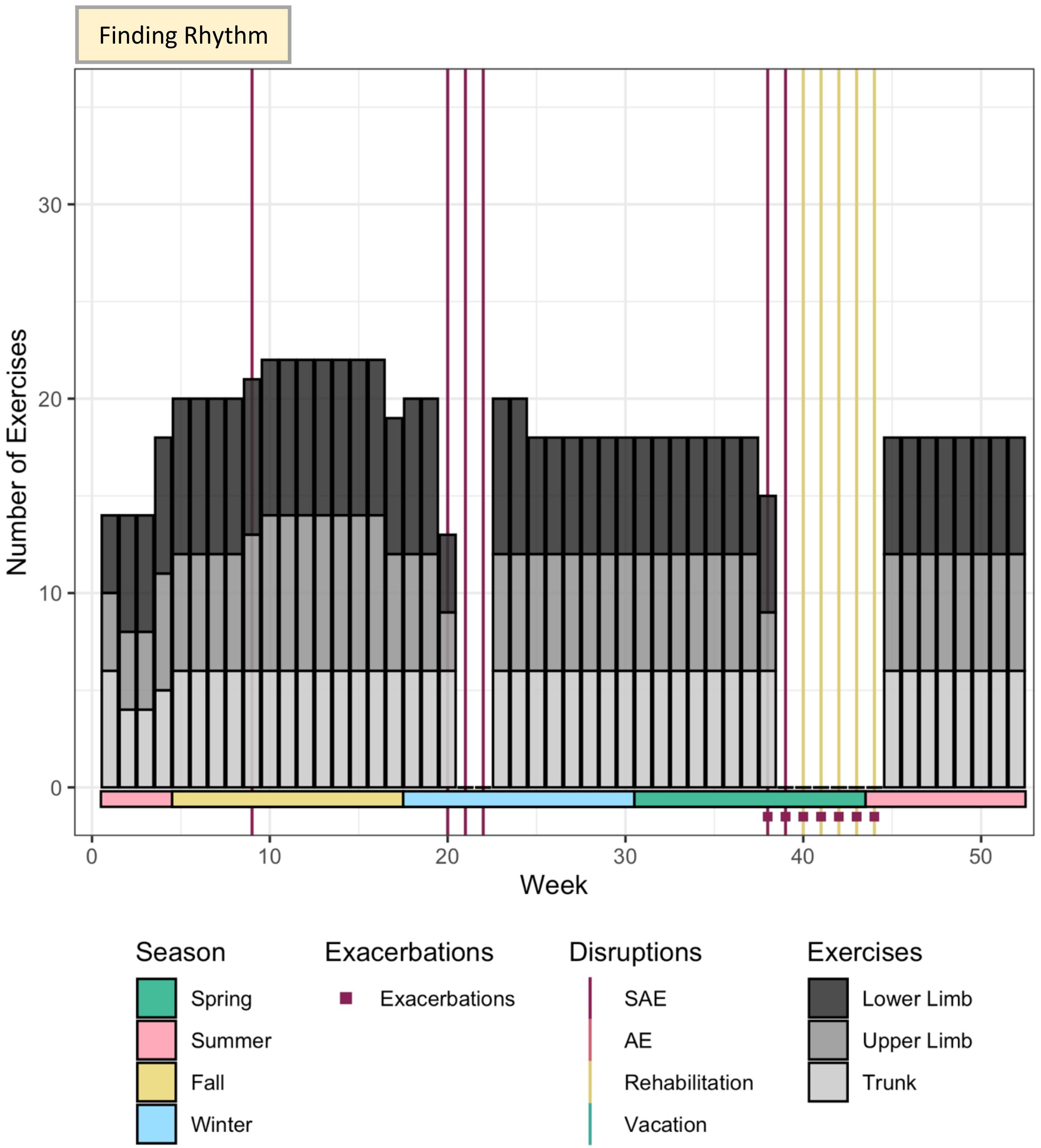

Participant 15

Adherence and Baseline Demographics

**Adherence: All Weeks (%)**	82.7
**Adherence: Healthy Weeks (%)**	94.9
**Bad Health (Weeks)**	13
**Age**	71
**Sex**	Female
**FEV1 (% Pred)**	20
**CRQ Dyspnea**	4
**Marital Status**	Single
**Living Situation**	Lives alone
**Comorbidities (n)**	3
**Sparring Partner**	Yes
**Set 2-Month Goal**	Yes
**Set 12-Month Goal**	Yes

Self-Efficacy

How confident are you in your ability to…

	3 mo.	6mo.	12 mo.
**Practice Daily**	9	-	8
**Practice Correctly**	9	-	10
**Adjust Intensity**	9	-	10
**Keep an Agenda**	9	-	8

Notes

The participant did not report engaging in physical activity other than HOMEX during the program. She reported experiencing positive effects of training in her daily life and was motivated to train to achieve greater health and fitness.

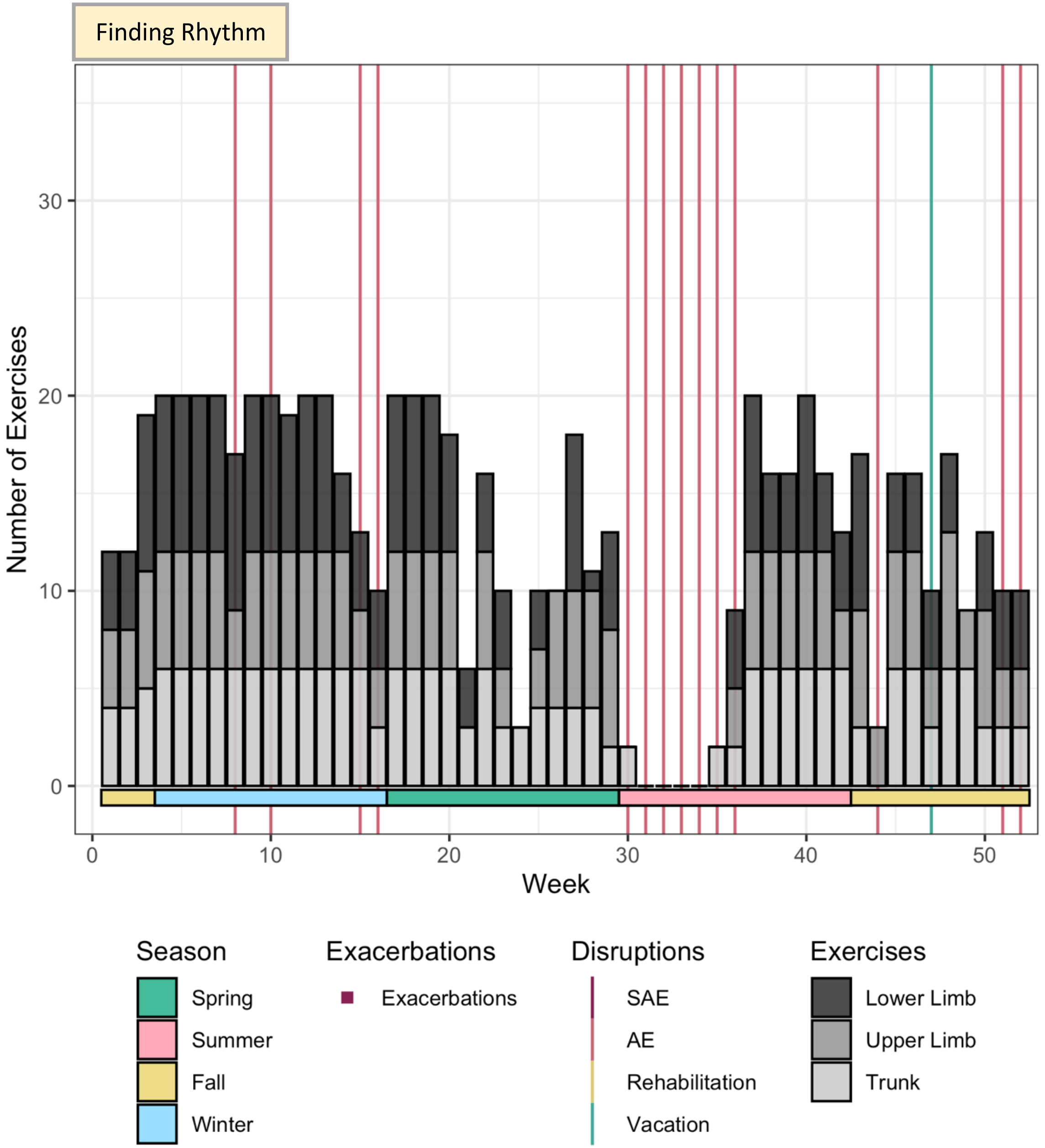

Participant 16

Adherence and Baseline Demographics

**Adherence: All Weeks (%)**	19.2
**Adherence: Healthy Weeks (%)**	29
**Bad Health (Weeks)**	9
**Age**	68
**Sex**	Male
**FEV1 (% Pred)**	50.5
**CRQ Dyspnea**	4
**Marital Status**	Married or partnership
**Living Situation**	Lives with partner or children
**Comorbidities (n)**	3
**Sparring Partner**	Yes
**Set 2-Month Goal**	Yes
**Set 12-Month Goal**	Yes

Self-Efficacy

How confident are you in your ability to…

	3 mo.	6mo.	12 mo.
**Practice Daily**	6	6	8
**Practice Correctly**	8	8	8
**Adjust Intensity**	7	7	8
**Keep an Agenda**	8	8	8

Notes

The participant regularly reported engaging in physical activities other than HOMEX throughout the year. He reported stopping HOMEX training due to joint pain, though he reported that he felt positive effects of training in his daily life. He was motivated to train to increase his health and fitness.

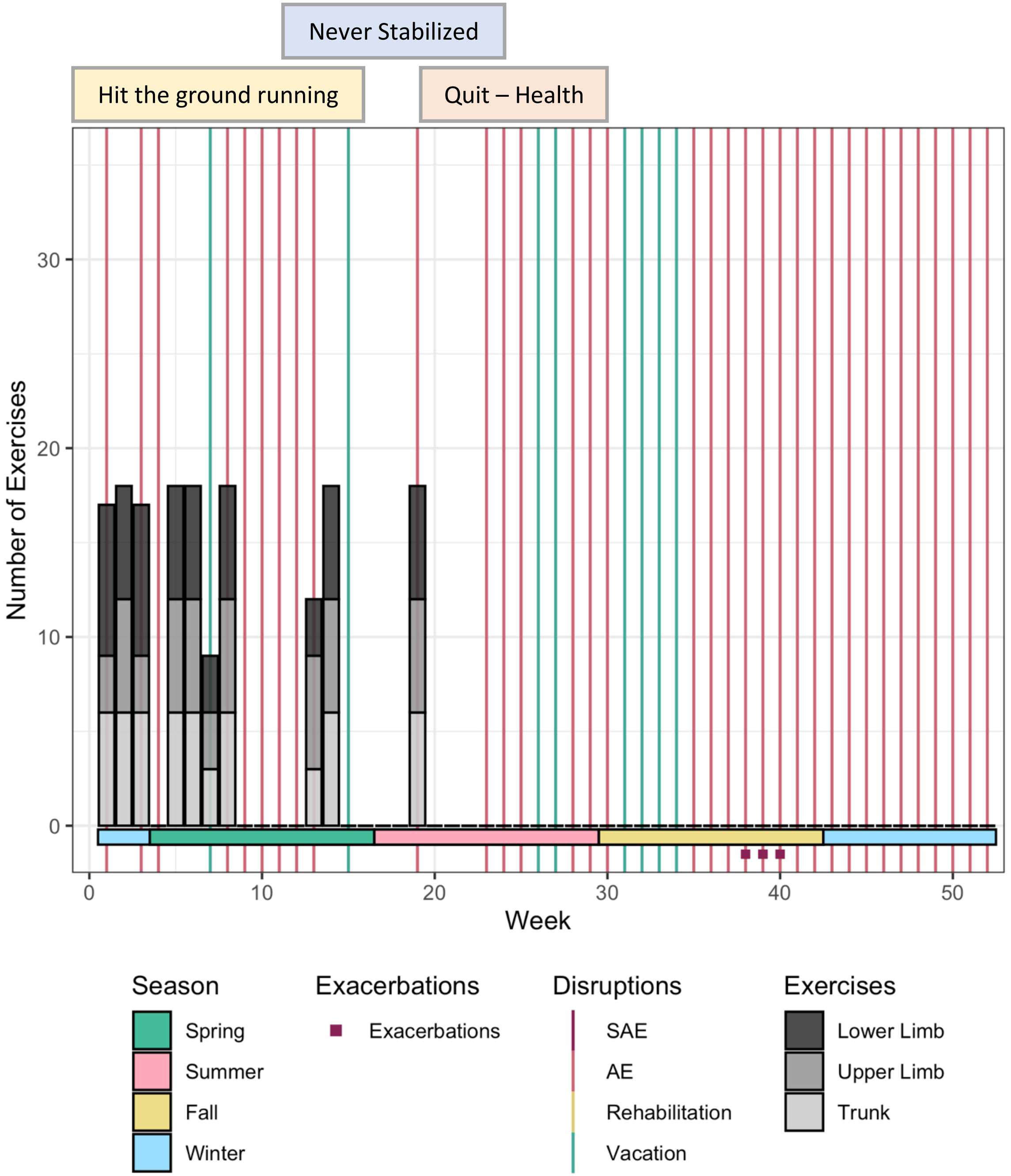

Participant 17

Adherence and Baseline Demographics

**Adherence: All Weeks (%)**	94.2
**Adherence: Healthy Weeks (%)**	100
**Bad Health (Weeks)**	12
**Age**	64
**Sex**	Female
**FEV1 (% Pred)**	57.9
**CRQ Dyspnea**	3.8
**Marital Status**	Married or partnership
**Living Situation**	Lives with partner or children
**Comorbidities (n)**	8
**Sparring Partner**	Yes
**Set 2-Month Goal**	Yes
**Set 12-Month Goal**	Yes

Self-Efficacy

How confident are you in your ability to…

	3 mo.	6mo.	12 mo.
**Practice Daily**	10	10	10
**Practice Correctly**	10	10	10
**Adjust Intensity**	10	10	10
**Keep an Agenda**	10	10	10

Notes

The participant occasionally reported engaging in physical activity other than HOMEX during the program. She reported experiencing positive effects of training in her daily life and was motivated to train because of her experience in pulmonary rehabilitation.

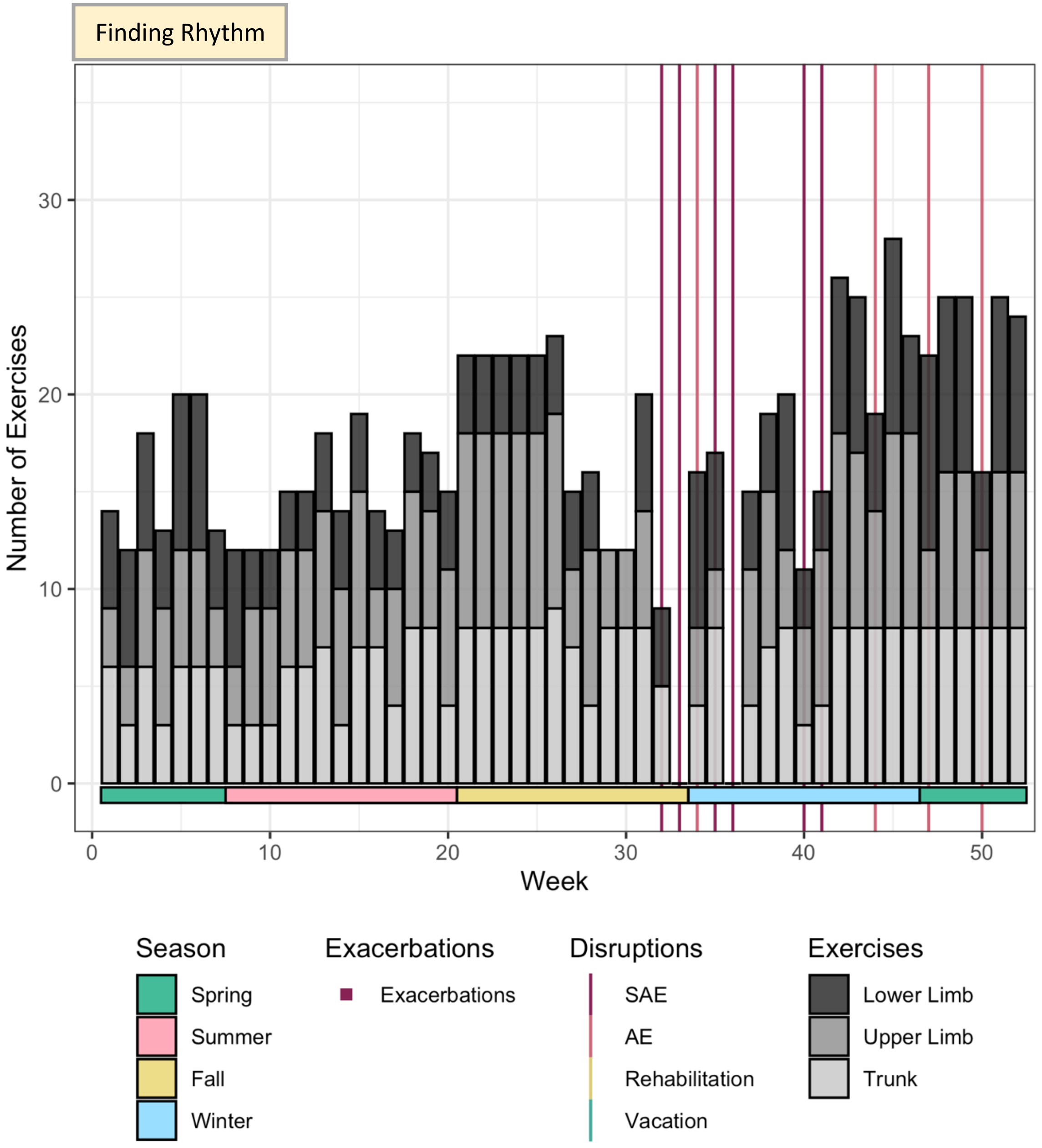

Participant 18

Adherence and Baseline Demographics

**Adherence: All Weeks (%)**	92.3
**Adherence: Healthy Weeks (%)**	97.7
**Bad Health (Weeks)**	8
**Age**	75
**Sex**	Female
**FEV1 (% Pred)**	27.8
**CRQ Dyspnea**	5
**Marital Status**	Married or partnership
**Living Situation**	Lives with partner or children
**Comorbidities (n)**	4
**Sparring Partner**	Yes
**Set 2-Month Goal**	Yes
**Set 12-Month Goal**	Yes

Self-Efficacy

How confident are you in your ability to…

	3 mo.	6mo.	12 mo.
**Practice Daily**	10	10	10
**Practice Correctly**	10	10	8
**Adjust Intensity**	10	10	10
**Keep an Agenda**	10	10	8

Notes

The participant regularly reported engaging in physical activity other than HOMEX during the program. She reported experiencing positive effects of training in her daily life and was motivated to train both by having a concrete goal and because of her positive experiences in pulmonary rehabilitation.

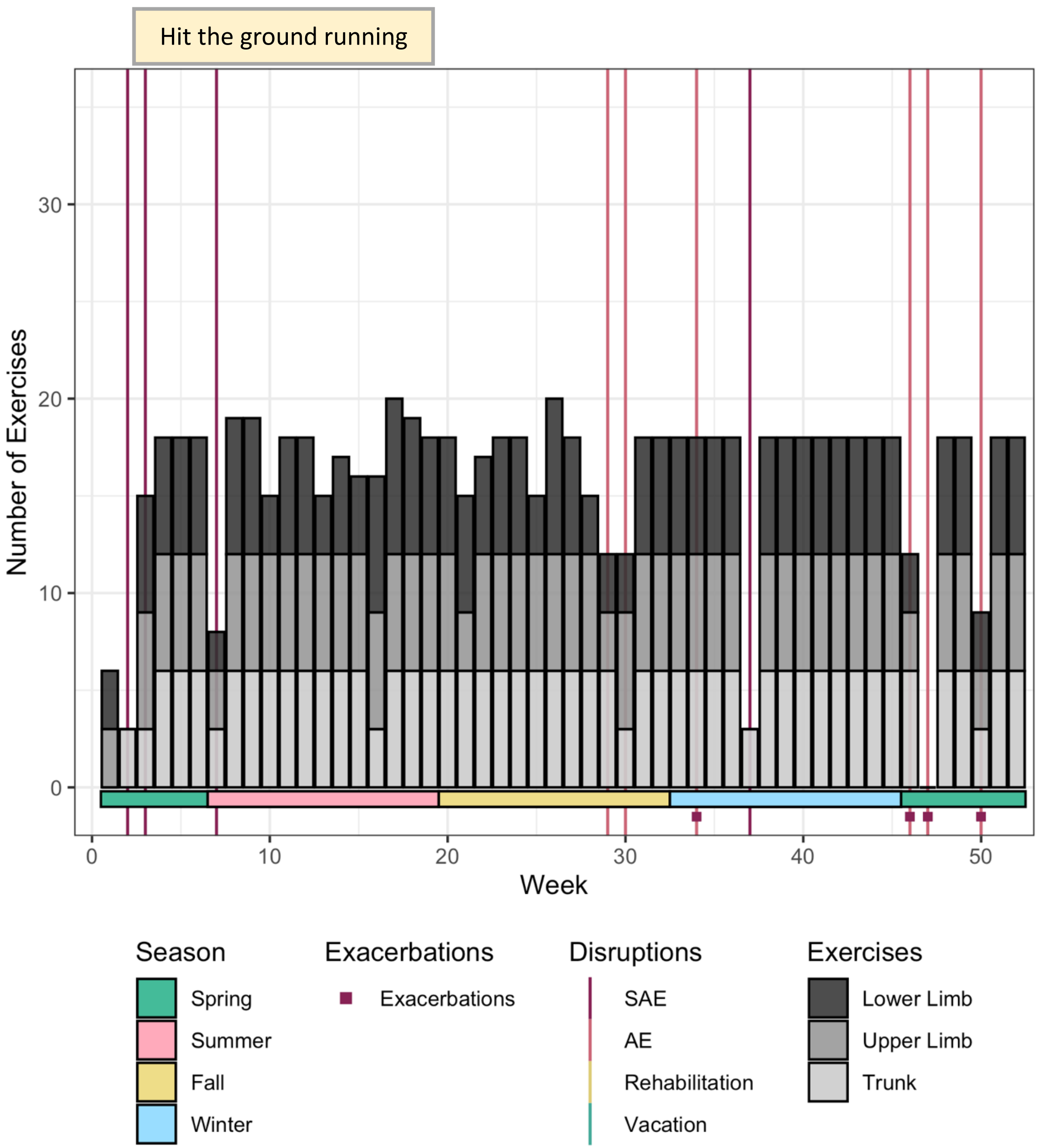

Participant 19

Adherence and Baseline Demographics

**Adherence: All Weeks (%)**	17.3
**Adherence: Healthy Weeks (%)**	18.6
**Bad Health (Weeks)**	9
**Age**	56
**Sex**	Male
**FEV1 (% Pred)**	30.4
**CRQ Dyspnea**	6
**Marital Status**	Divorced or separated
**Living Situation**	Lives alone
**Comorbidities (n)**	2
**Sparring Partner**	No
**Set 2-Month Goal**	Yes
**Set 12-Month Goal**	No

Self-Efficacy

How confident are you in your ability to…

	3 mo.	6mo.	12 mo.
**Practice Daily**	10	9	10
**Practice Correctly**	10	9	8
**Adjust Intensity**	10	9	10
**Keep an Agenda**	10	9	7

Notes

The participant regularly reported engaging in physical activity other than HOMEX throughout the program. This led to low adherence to HOMEX, since it left little time for and was less comprehensive than his other activities. He experienced positive effects of training in his daily life. He was motivated to train both because he understood the importance of training and wished to increase his independence and quality of life.

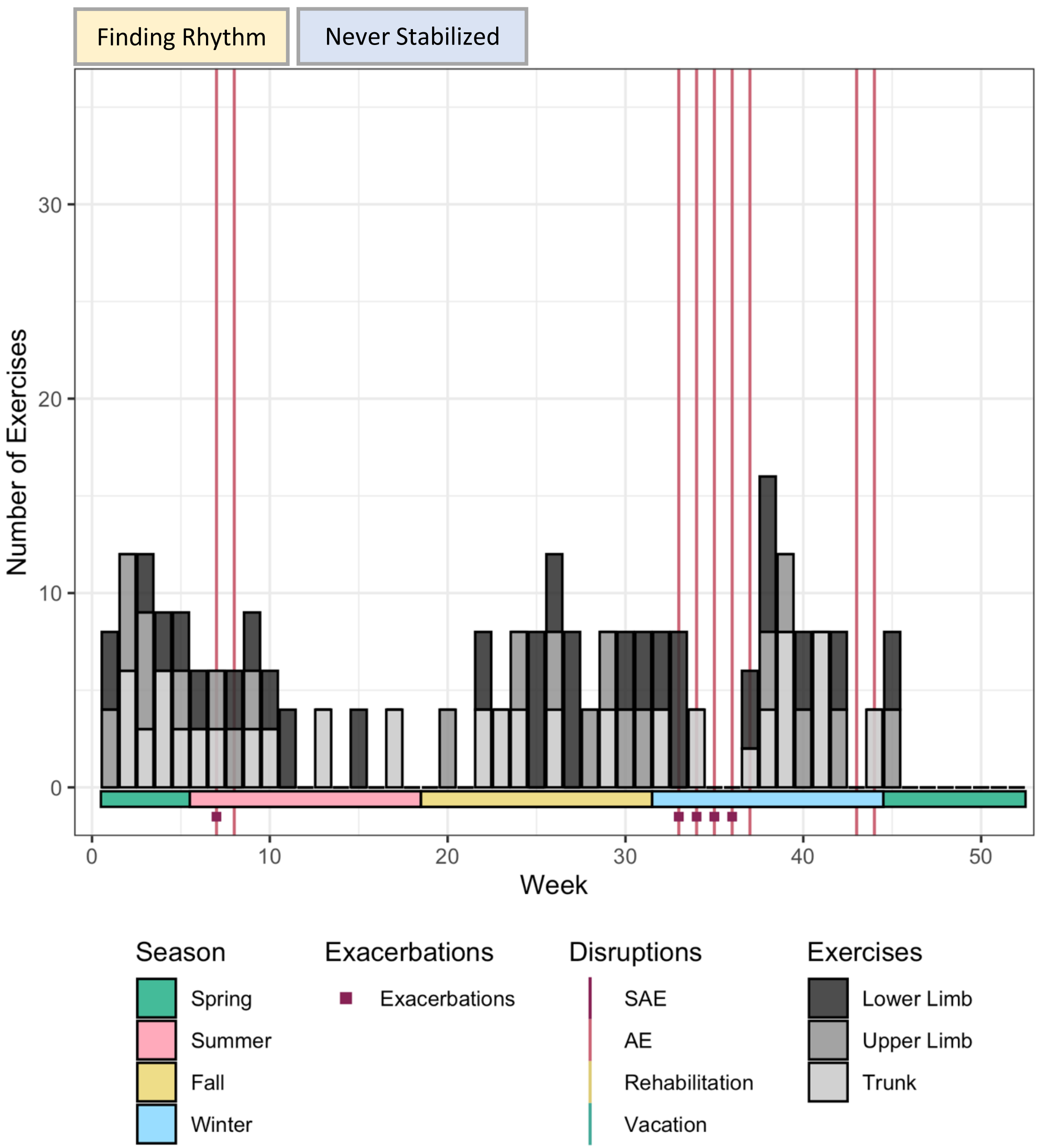

Participant 20

Adherence and Baseline Demographics

**Adherence: All Weeks (%)**	96.2
**Adherence: Healthy Weeks (%)**	96.2
**Bad Health (Weeks)**	0
**Age**	64
**Sex**	Female
**FEV1 (% Pred)**	40.7
**CRQ Dyspnea**	3.5
**Marital Status**	Divorced or separated
**Living Situation**	Lives alone
**Comorbidities (n)**	1
**Sparring Partner**	Yes
**Set 2-Month Goal**	Yes
**Set 12-Month Goal**	Yes

Self-Efficacy

How confident are you in your ability to…

	3 mo.	6mo.	12 mo.
**Practice Daily**	10	10	10
**Practice Correctly**	9	10	10
**Adjust Intensity**	10	10	10
**Keep an Agenda**	10	10	10

Notes

The participant regularly reported engaging in physical activity other than HOMEX during the program. She reported experiencing positive effects of training in her daily life and was motivated to train both by her experiences in pulmonary rehabilitation and because she understood the importance of training.

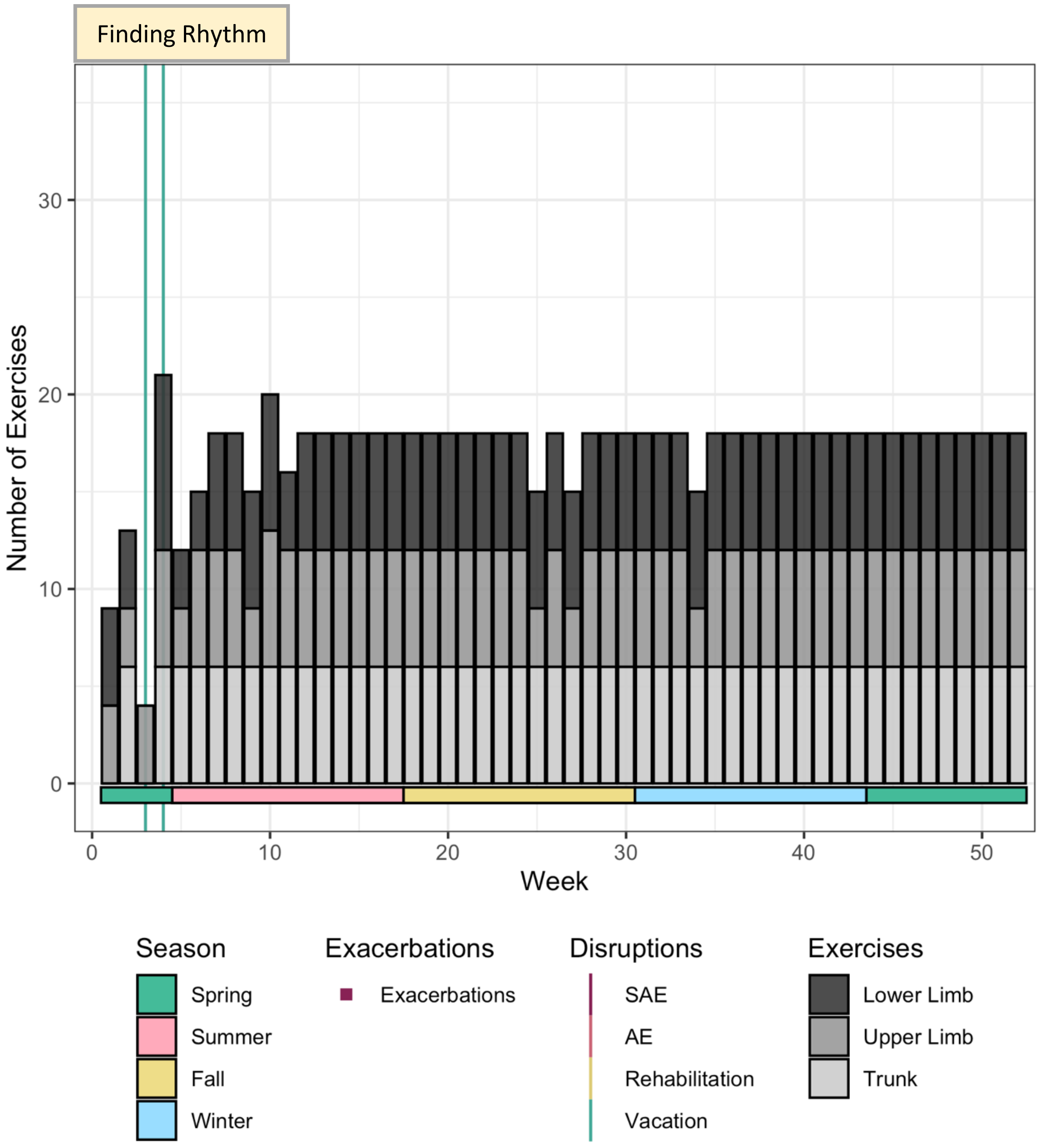

Participant 21

Adherence and Baseline Demographics

**Adherence: All Weeks (%)**	88.5
**Adherence: Healthy Weeks (%)**	97.6
**Bad Health (Weeks)**	10
**Age**	62
**Sex**	Female
**FEV1 (% Pred)**	33.9
**CRQ Dyspnea**	2
**Marital Status**	Married or partnership
**Living Situation**	Lives with partner or children
**Comorbidities (n)**	3
**Sparring Partner**	Yes
**Set 2-Month Goal**	Yes
**Set 12-Month Goal**	Yes

Self-Efficacy

How confident are you in your ability to…

	3 mo.	6mo.	12 mo.
**Practice Daily**	9	7	8
**Practice Correctly**	9	8	9
**Adjust Intensity**	7	9	7
**Keep an Agenda**	9	8	8

Notes

The participant regularly reported engaging in physical activity other than HOMEX during the program. She reported experiencing positive effects of training in her daily life and was motivated to train both because she understood the importance of training and because she wished to increase her independence and quality of life.

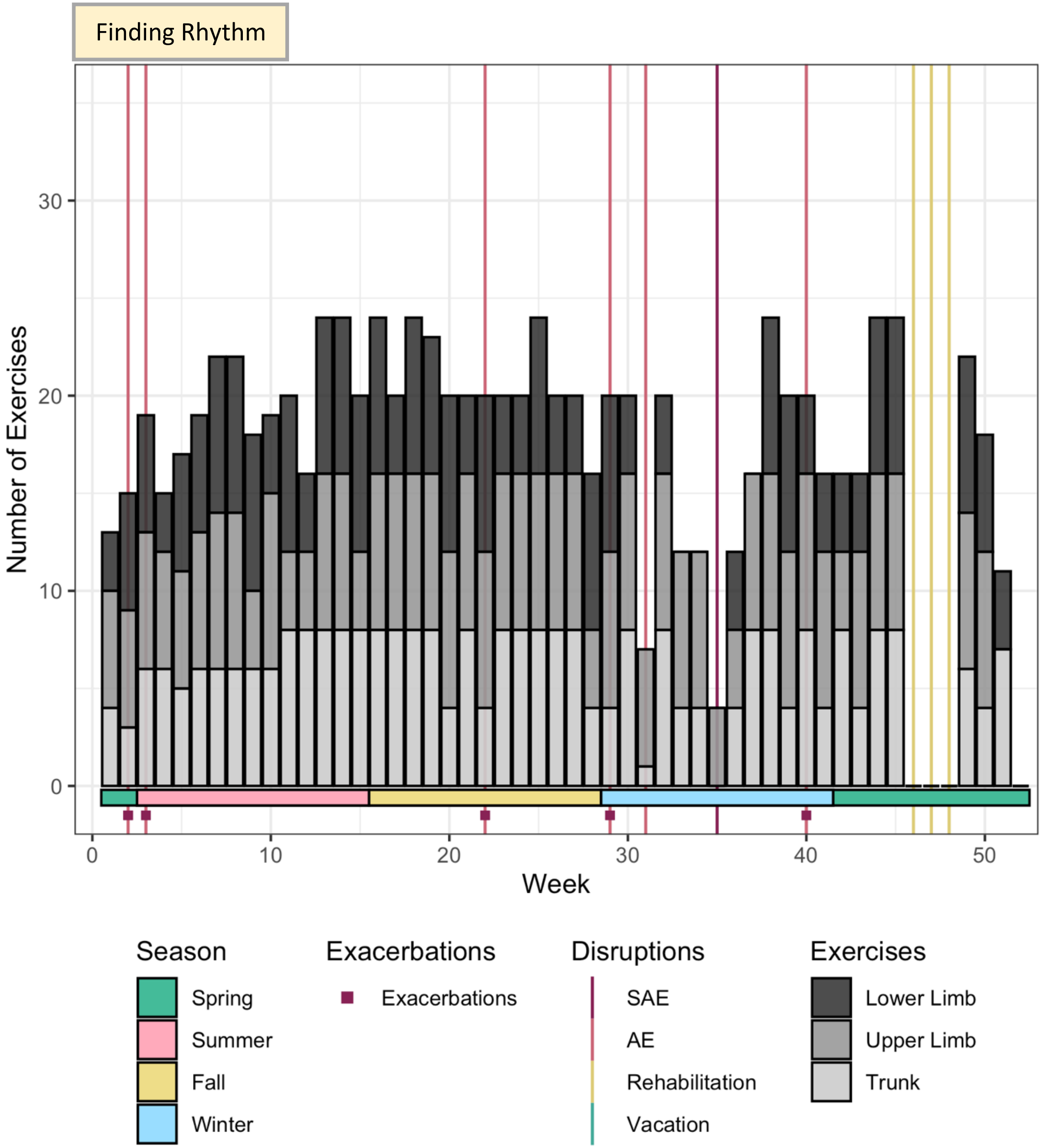

Participant 22

Adherence and Baseline Demographics

**Adherence: All Weeks (%)**	23.1
**Adherence: Healthy Weeks (%)**	29.7
**Bad Health (Weeks)**	15
**Age**	59
**Sex**	Female
**FEV1 (% Pred)**	17.5
**CRQ Dyspnea**	3
**Marital Status**	Married or partnership
**Living Situation**	Lives with partner or children
**Comorbidities (n)**	6
**Sparring Partner**	Yes
**Set 2-Month Goal**	Yes
**Set 12-Month Goal**	Yes

Self-Efficacy

How confident are you in your ability to…

	3 mo.	6mo.	12 mo.
**Practice Daily**	-	-	6
**Practice Correctly**	-	6	8
**Adjust Intensity**	-	8	10
**Keep an Agenda**	-	8	6

Notes

The participant experienced a severe exacerbation in week 20 and massive dyspnea thereafter. She verbally reported occasional training during coaching calls, but stopped documenting her training for the remainder of the study. However, she reported experiencing positive effects of training. She was originally motivated to train because of the coach and by setting a concrete goal.

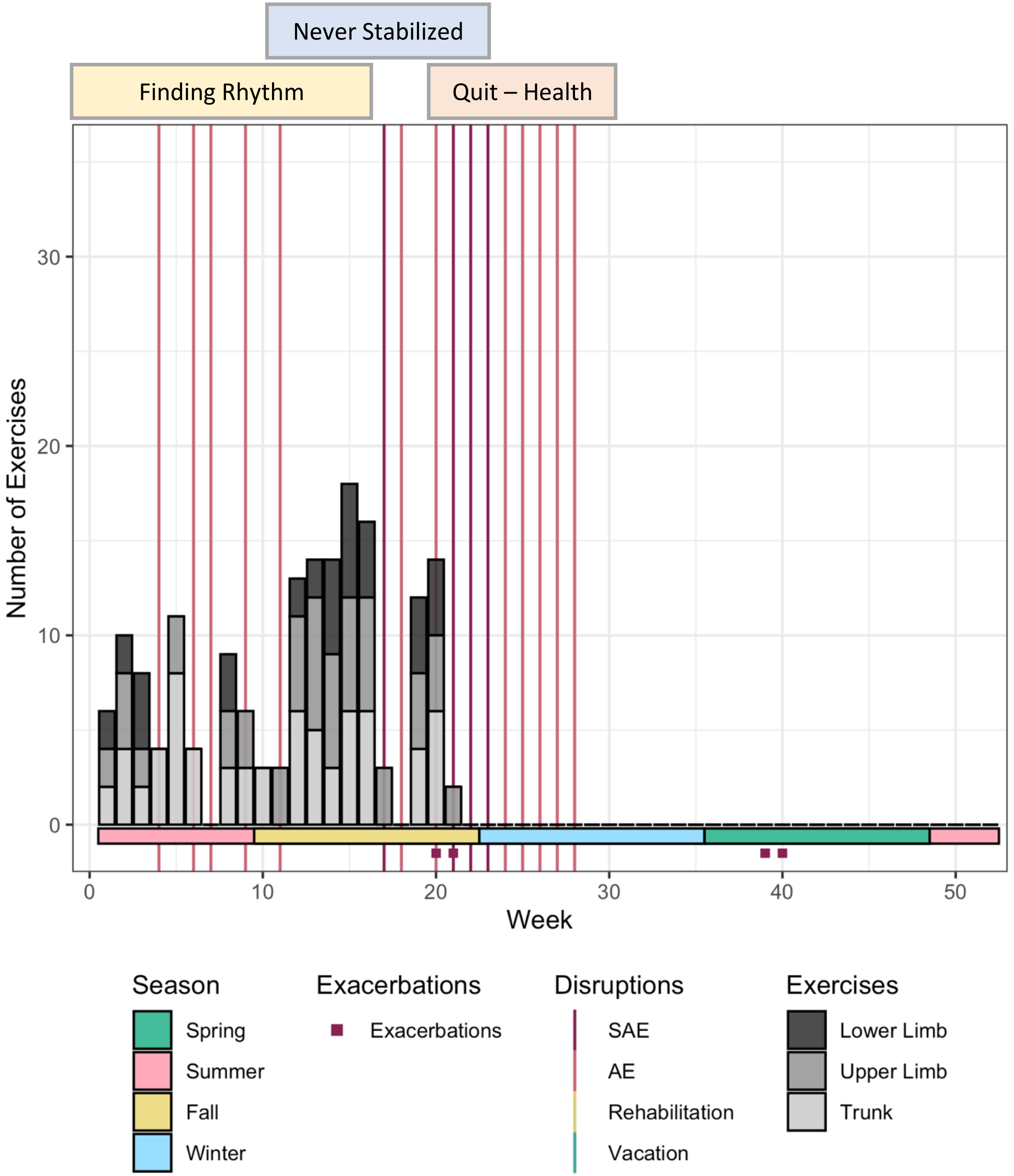

Participant 23

Adherence and Baseline Demographics

**Adherence: All Weeks (%)**	80.8
**Adherence: Healthy Weeks (%)**	84
**Bad Health (Weeks)**	2
**Age**	72
**Sex**	Female
**FEV1 (% Pred)**	29.5
**CRQ Dyspnea**	4.8
**Marital Status**	Married or partnership
**Living Situation**	Lives with partner or children
**Comorbidities (n)**	6
**Sparring Partner**	Yes
**Set 2-Month Goal**	Yes
**Set 12-Month Goal**	Yes

Self-Efficacy

How confident are you in your ability to…

	3 mo.	6mo.	12 mo.
**Practice Daily**	10	9	8
**Practice Correctly**	10	10	8
**Adjust Intensity**	10	10	8
**Keep an Agenda**	10	10	8

Notes

The participant did not report engaging in physical activity other than HOMEX during the program. She reported experiencing positive effects of training in her daily life and was motivated to train both by her experiences in pulmonary rehabilitation.

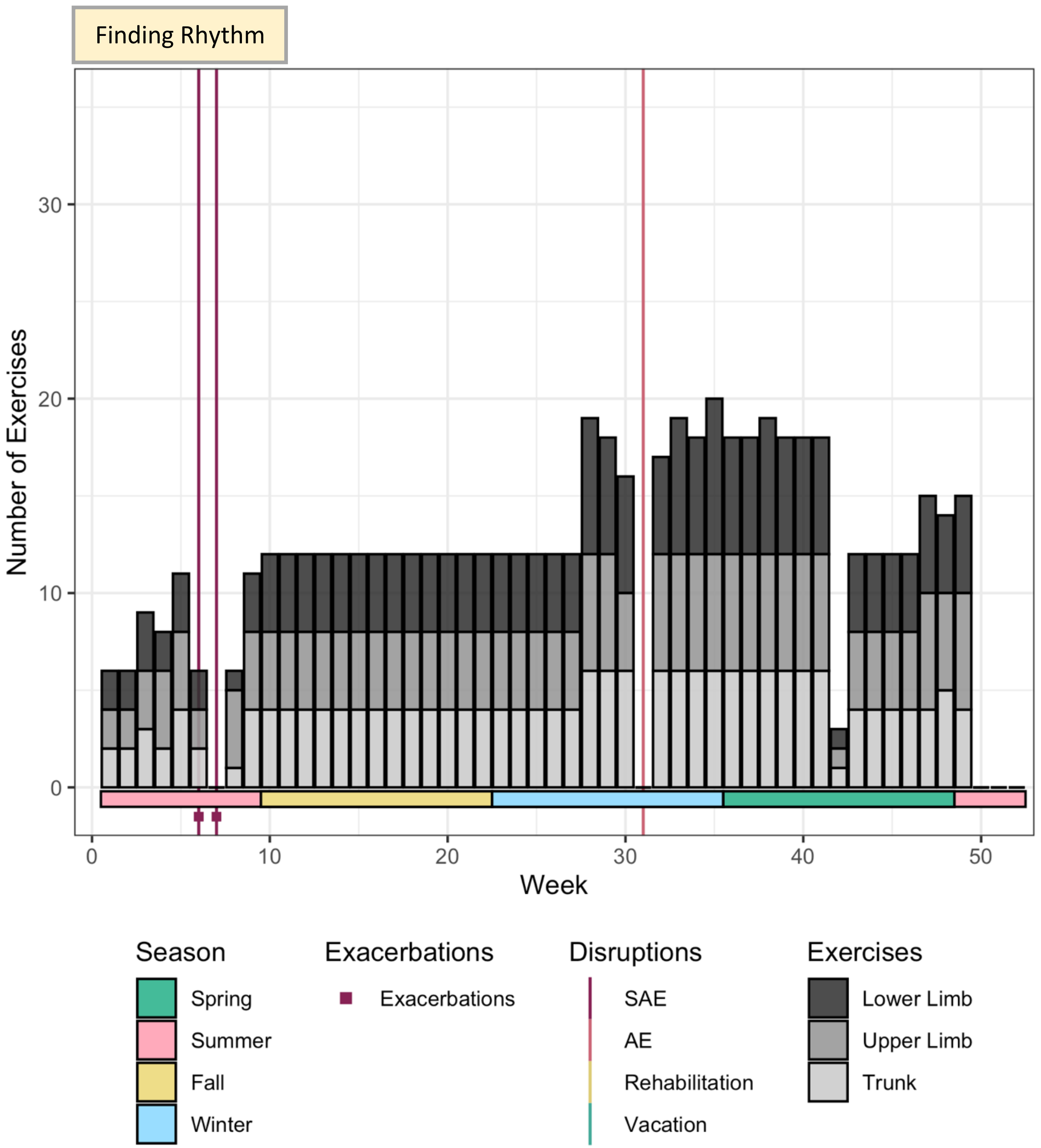

Participant 24

Adherence and Baseline Demographics

**Adherence: All Weeks (%)**	98.1
**Adherence: Healthy Weeks (%)**	97.9
**Bad Health (Weeks)**	4
**Age**	72
**Sex**	Male
**FEV1 (% Pred)**	31.4
**CRQ Dyspnea**	6
**Marital Status**	Married or partnership
**Living Situation**	Lives with partner or children
**Comorbidities (n)**	4
**Sparring Partner**	Yes
**Set 2-Month Goal**	Yes
**Set 12-Month Goal**	Yes

Self-Efficacy

How confident are you in your ability to…

	**3 mo.**	**6mo.**	**12 mo.**
**Practice Daily**	10	10	10
**Practice Correctly**	10	9	10
**Adjust Intensity**	9	10	10
**Keep an Agenda**	10	10	10

Notes

The participant regularly reported engaging in physical activity other than HOMEX during the program. He reported experiencing positive effects of training in his daily life and was motivated to train because he understand its importance, he wished to increase his health and fitness, and he wished to help develop something useful for other patients.

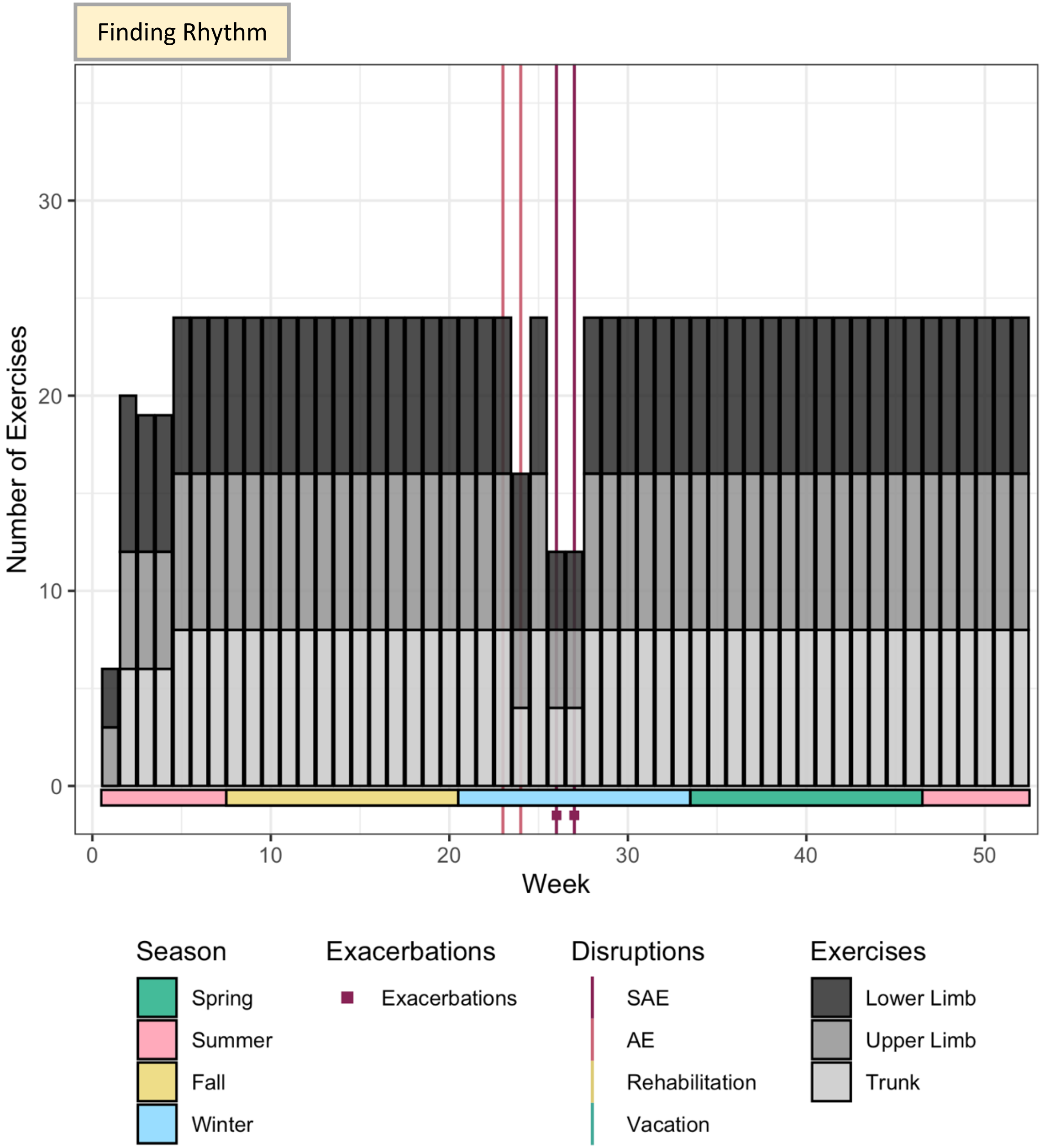

Participant 25

Adherence and Baseline Demographics

**Adherence: All Weeks (%)**	1.9
**Adherence: Healthy Weeks (%)**	1.9
**Bad Health (Weeks)**	0
**Age**	79
**Sex**	Male
**FEV1 (% Pred)**	54.7
**CRQ Dyspnea**	3
**Marital Status**	Married or partnership
**Living Situation**	Lives with partner or children
**Comorbidities (n)**	8
**Sparring Partner**	Yes
**Set 2-Month Goal**	No
**Set 12-Month Goal**	No

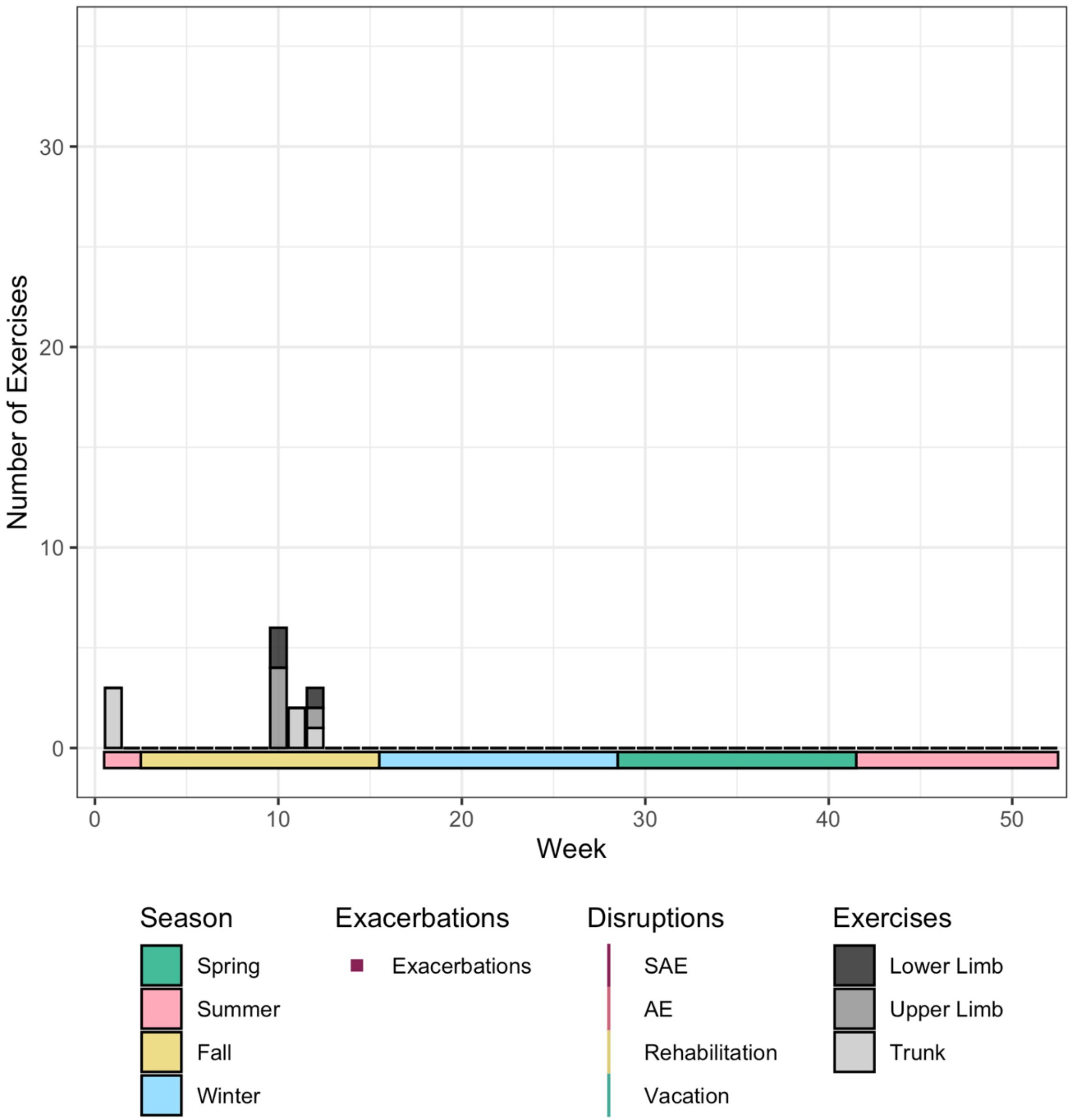

Self-Efficacy

How confident are you in your ability to…

	3 mo.	6mo.	12 mo.
**Practice Daily**	-	3	2
**Practice Correctly**	-	7	6
**Adjust Intensity**	-	5	6
**Keep an Agenda**	-	5	2

Notes

This participant's reasons for not training are unclear. No disruptions or other training were documented over the year. He did not report any experiences or motivational factors during the exit interviews.

Participant 26

Adherence and Baseline Demographics

**Adherence: All Weeks (%)**	75
**Adherence: Healthy Weeks (%)**	97.2
**Bad Health (Weeks)**	15
**Age**	55
**Sex**	Male
**FEV1 (% Pred)**	26.2
**CRQ Dyspnea**	6.2
**Marital Status**	Divorced or separated
**Living Situation**	Community or assisted living
**Comorbidities (n)**	8
**Sparring Partner**	No
**Set 2-Month Goal**	No
**Set 12-Month Goal**	Yes

Self-Efficacy

How confident are you in your ability to…

	3 mo.	6mo.	12 mo.
**Practice Daily**	10	8	10
**Practice Correctly**	10	9	10
**Adjust Intensity**	10	9	10
**Keep an Agenda**	10	9	10

Notes

The participant did not report engaging in physical activity other than HOMEX during the program. He reported experiencing positive effects of training in her daily life and was motivated to train because of his experiences in pulmonary rehabilitation.

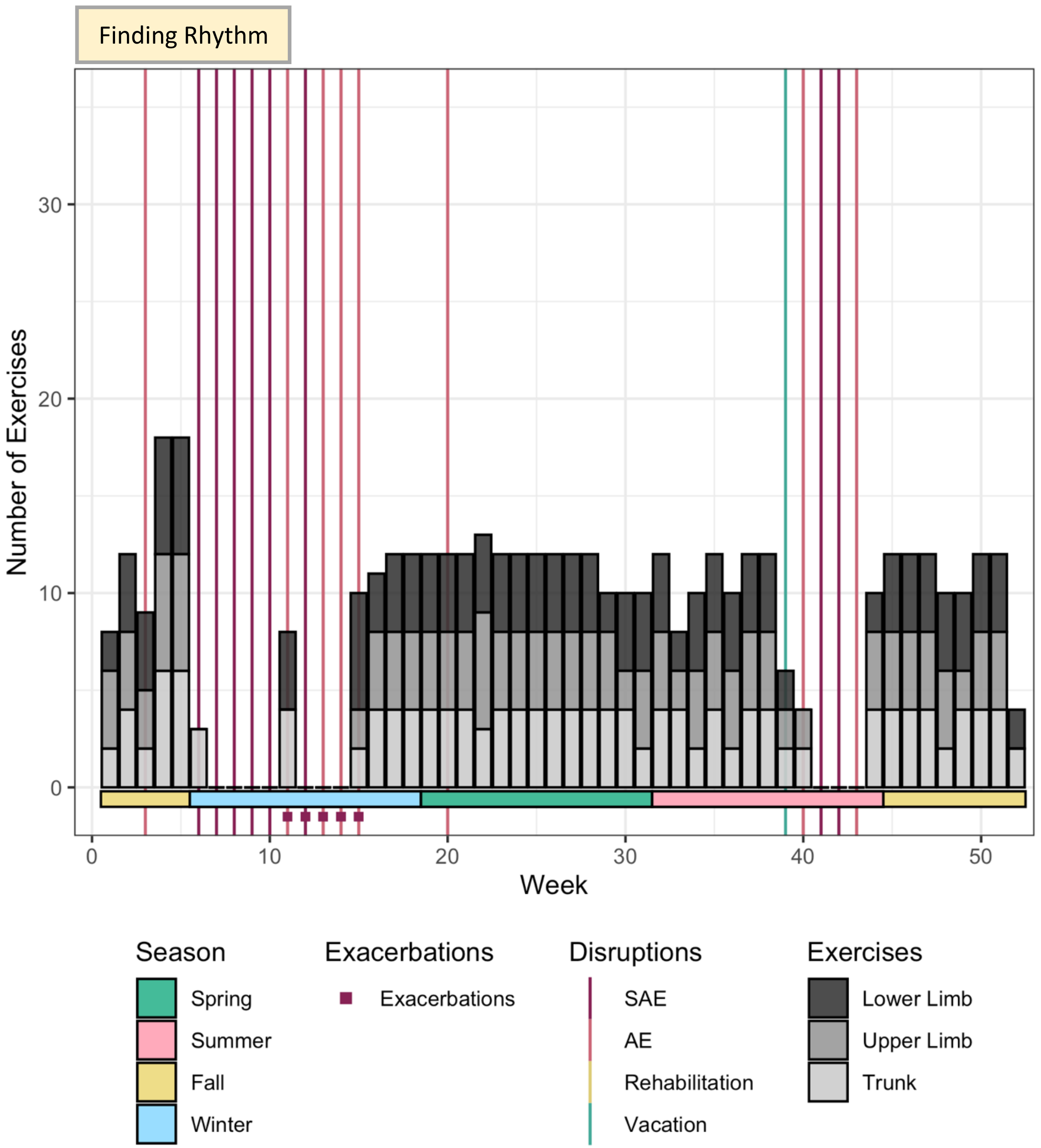

Participant 27

Adherence and Baseline Demographics

**Adherence: All Weeks (%)**	98.1
**Adherence: Healthy Weeks (%)**	100
**Bad Health (Weeks)**	2
**Age**	63
**Sex**	Female
**FEV1 (% Pred)**	38.1
**CRQ Dyspnea**	4.2
**Marital Status**	Married or partnership
**Living Situation**	Lives with partner or children
**Comorbidities (n)**	3
**Sparring Partner**	Yes
**Set 2-Month Goal**	No
**Set 12-Month Goal**	No

Self-Efficacy

How confident are you in your ability to…

	3 mo.	6mo.	12 mo.
**Practice Daily**	8	9	5
**Practice Correctly**	8	7	10
**Adjust Intensity**	8	7	5
**Keep an Agenda**	8	9	10

Notes

The participant regularly reported engaging in physical activity other than HOMEX during the program. She reported experiencing positive effects of training in her daily life. She was motivated to train because she understood the importance of training, because she wished to improve her health and fitness, and because she felt committed to the study.

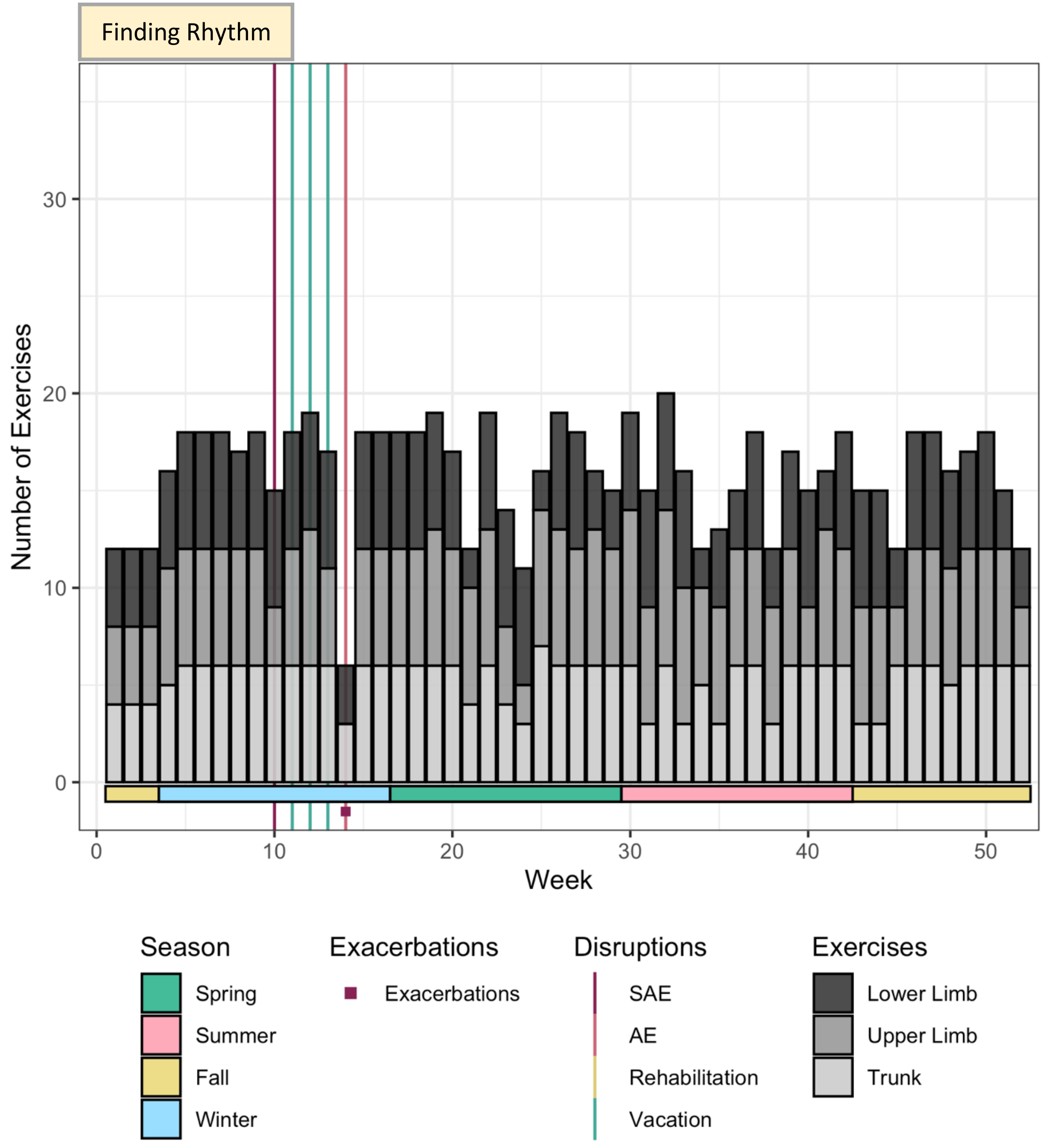

Participant 28

Adherence and Baseline Demographics

**Adherence: All Weeks (%)**	36.5
**Adherence: Healthy Weeks (%)**	44.2
**Bad Health (Weeks)**	9
**Age**	68
**Sex**	Male
**FEV1 (% Pred)**	24.4
**CRQ Dyspnea**	4.8
**Marital Status**	Married or partnership
**Living Situation**	Lives with partner or children
**Comorbidities (n)**	6
**Sparring Partner**	Yes
**Set 2-Month Goal**	No
**Set 12-Month Goal**	Yes

Self-Efficacy

How confident are you in your ability to…

	3 mo.	6mo.	12 mo.
**Practice Daily**	-	-	3
**Practice Correctly**	-	-	6
**Adjust Intensity**	-	-	8
**Keep an Agenda**	-	-	5

Notes

The participant experienced sudden worsening and massive dyspnea at week 24, which eventually resulted in hospitalization and rehabilitation. He re-started training at a lower, 'sub-adherent' rate when he felt well enough to do so. He reported feeling positive effects from training and continued because he understood its importance.

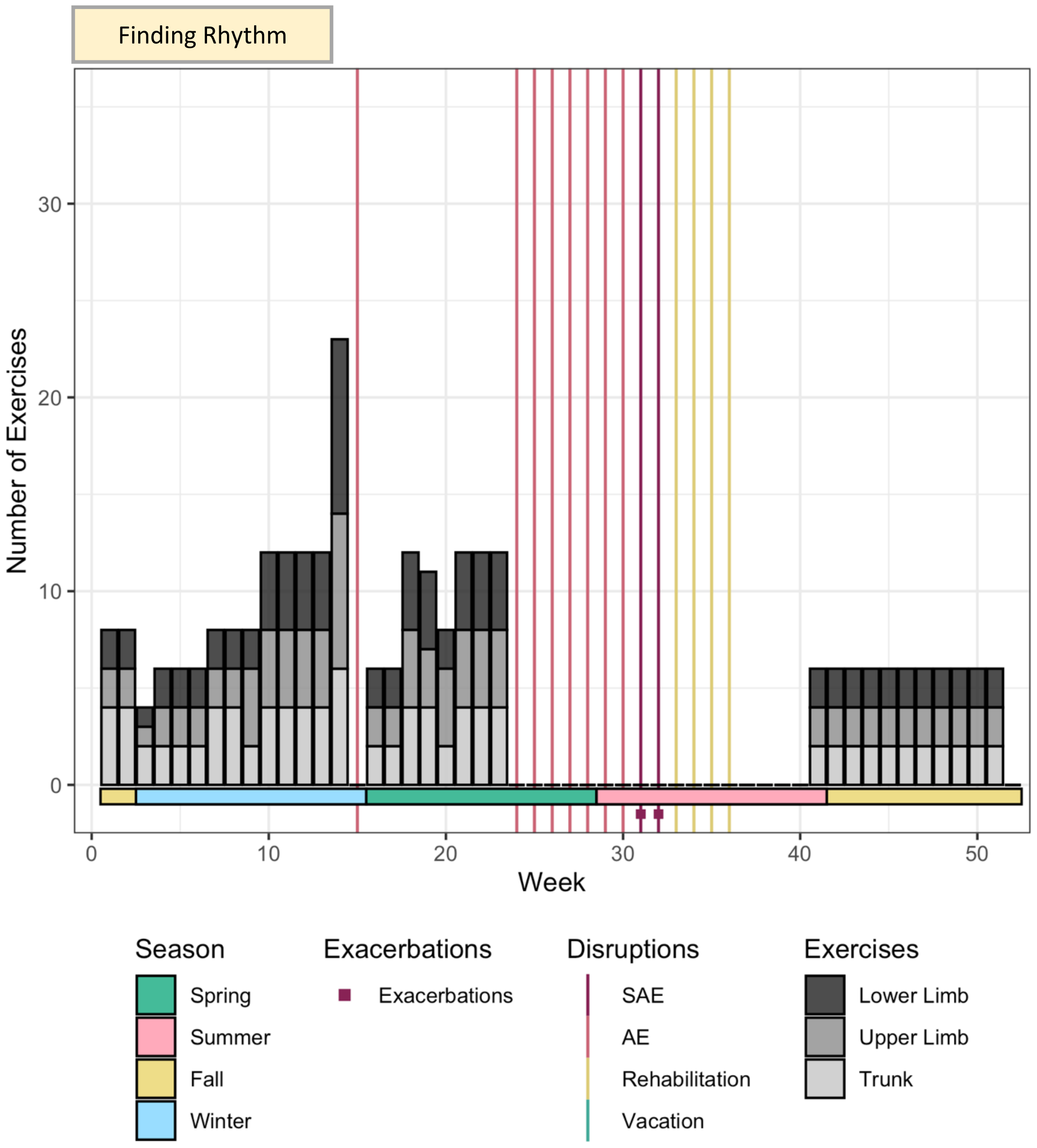

Participant 29

Adherence and Baseline Demographics

**Adherence: All Weeks (%)**	75
**Adherence: Healthy Weeks (%)**	97.1
**Bad Health (Weeks)**	18
**Age**	72
**Sex**	Male
**FEV1 (% Pred)**	72.9
**CRQ Dyspnea**	4
**Marital Status**	Single
**Living Situation**	Lives alone
**Comorbidities (n)**	8
**Sparring Partner**	No
**Set 2-Month Goal**	No
**Set 12-Month Goal**	Yes

Self-Efficacy

How confident are you in your ability to…

	3 mo.	6mo.	12 mo.
**Practice Daily**	7	8	10
**Practice Correctly**	10	8	10
**Adjust Intensity**	8	7	10
**Keep an Agenda**	8	9	9

Notes

The participant did not report engaging in physical activity other than HOMEX during the program. He reported experiencing positive effects of training in his daily life. He was motivated to train despite frequent exacerbations and hospitalizations because of his experiences in pulmonary rehabilitation, the importance of training, a wish to increase his health and fitness, and because he set a concrete goal.

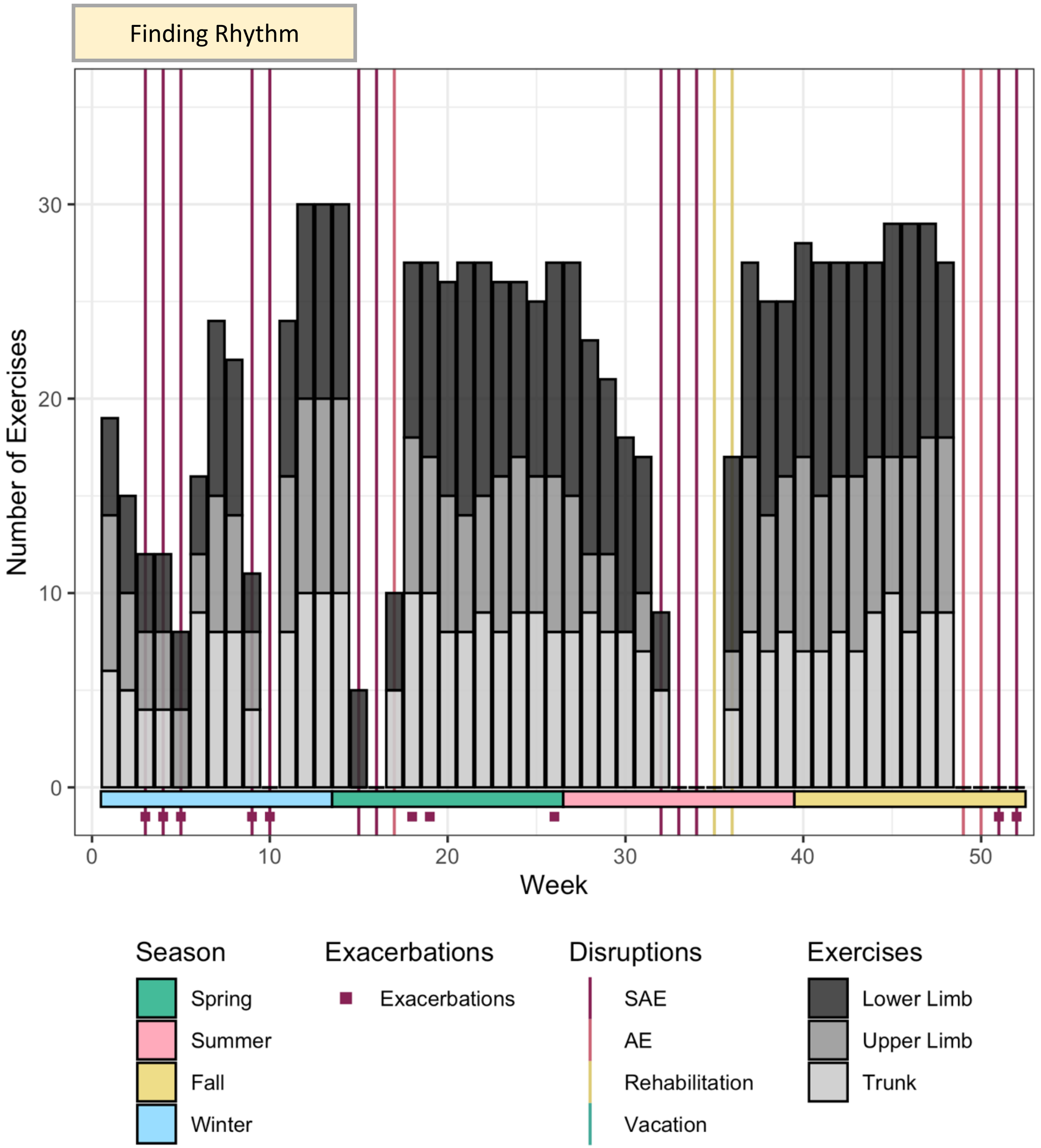

Participant 30

Adherence and Baseline Demographics

**Adherence: All Weeks (%)**	76.9
**Adherence: Healthy Weeks (%)**	92.9
**Bad Health (Weeks)**	10
**Age**	60
**Sex**	Female
**FEV1 (% Pred)**	28.4
**CRQ Dyspnea**	3.3
**Marital Status**	Married or partnership
**Living Situation**	Lives with partner or children
**Comorbidities (n)**	2
**Sparring Partner**	Yes
**Set 2-Month Goal**	Yes
**Set 12-Month Goal**	Yes

Self-Efficacy

How confident are you in your ability to…

	3 mo.	6mo.	12 mo.
**Practice Daily**	10	10	10
**Practice Correctly**	10	10	10
**Adjust Intensity**	10	10	10
**Keep an Agenda**	10	10	10

Notes

At first the participant reported engaging in other physical activity, but transitioned mostly to HOMEX training after week 16. She prepared for and underwent a lung transplantation near the end of the program. She reported experiencing positive effects of training in her daily life and was motivated to train both because of her concrete goal and to improve her health and fitness.

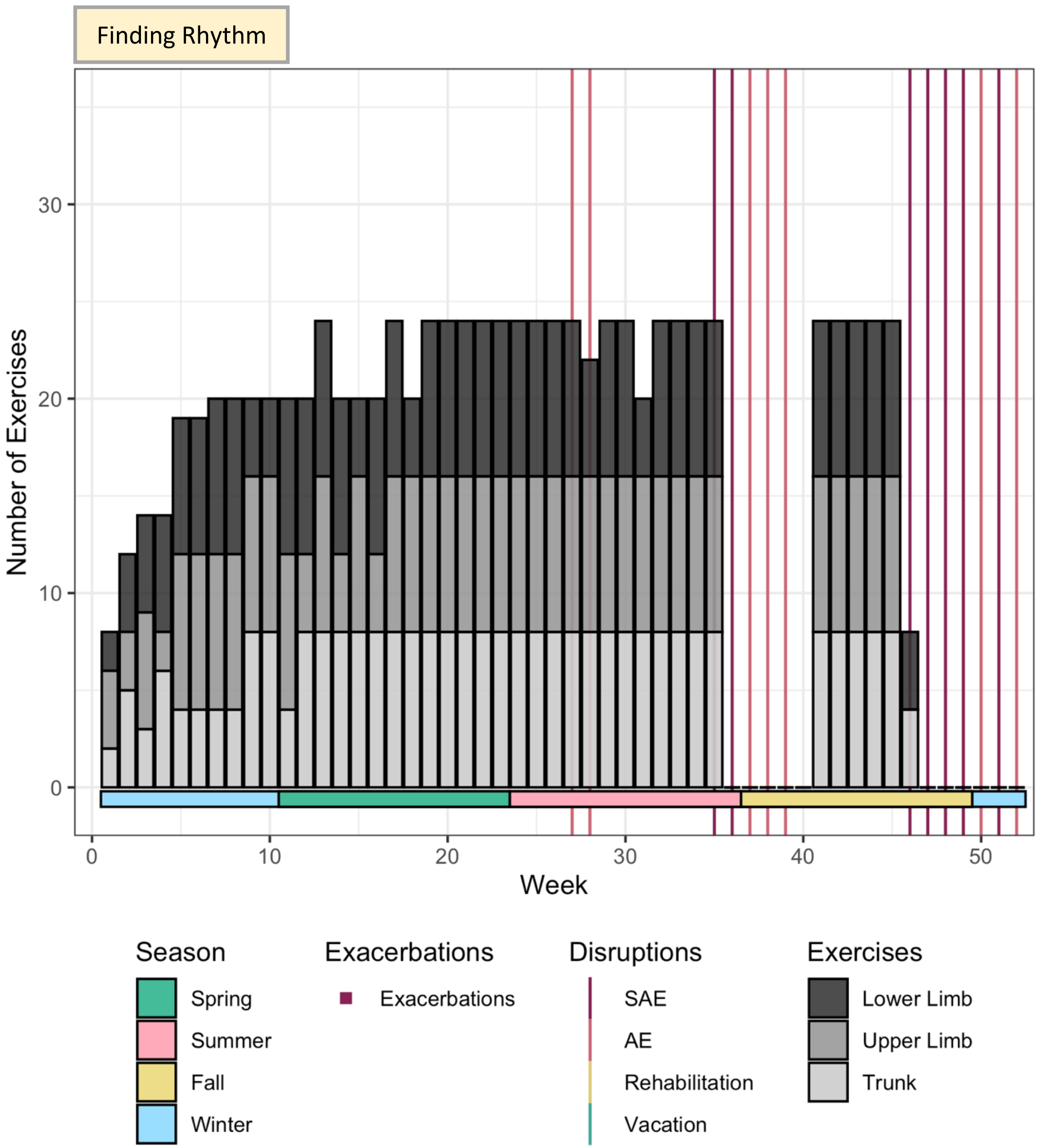

Participant 31

Adherence and Baseline Demographics

**Adherence: All Weeks (%)**	94.2
**Adherence: Healthy Weeks (%)**	95.9
**Bad Health (Weeks)**	3
**Age**	60
**Sex**	Female
**FEV1 (% Pred)**	68
**CRQ Dyspnea**	6
**Marital Status**	Single
**Living Situation**	Lives alone
**Comorbidities (n)**	2
**Sparring Partner**	Yes
**Set 2-Month Goal**	Yes
**Set 12-Month Goal**	Yes

Self-Efficacy

How confident are you in your ability to…

	3 mo.	6mo.	12 mo.
**Practice Daily**	-	9	7
**Practice Correctly**	-	10	10
**Adjust Intensity**	-	10	9
**Keep an Agenda**	-	9	3

Notes

The participant regularly reported engaging in physical activity other than HOMEX during the program. She reported experiencing positive effects of training in her daily life and was motivated to train because of her experiences in pulmonary rehabilitation.

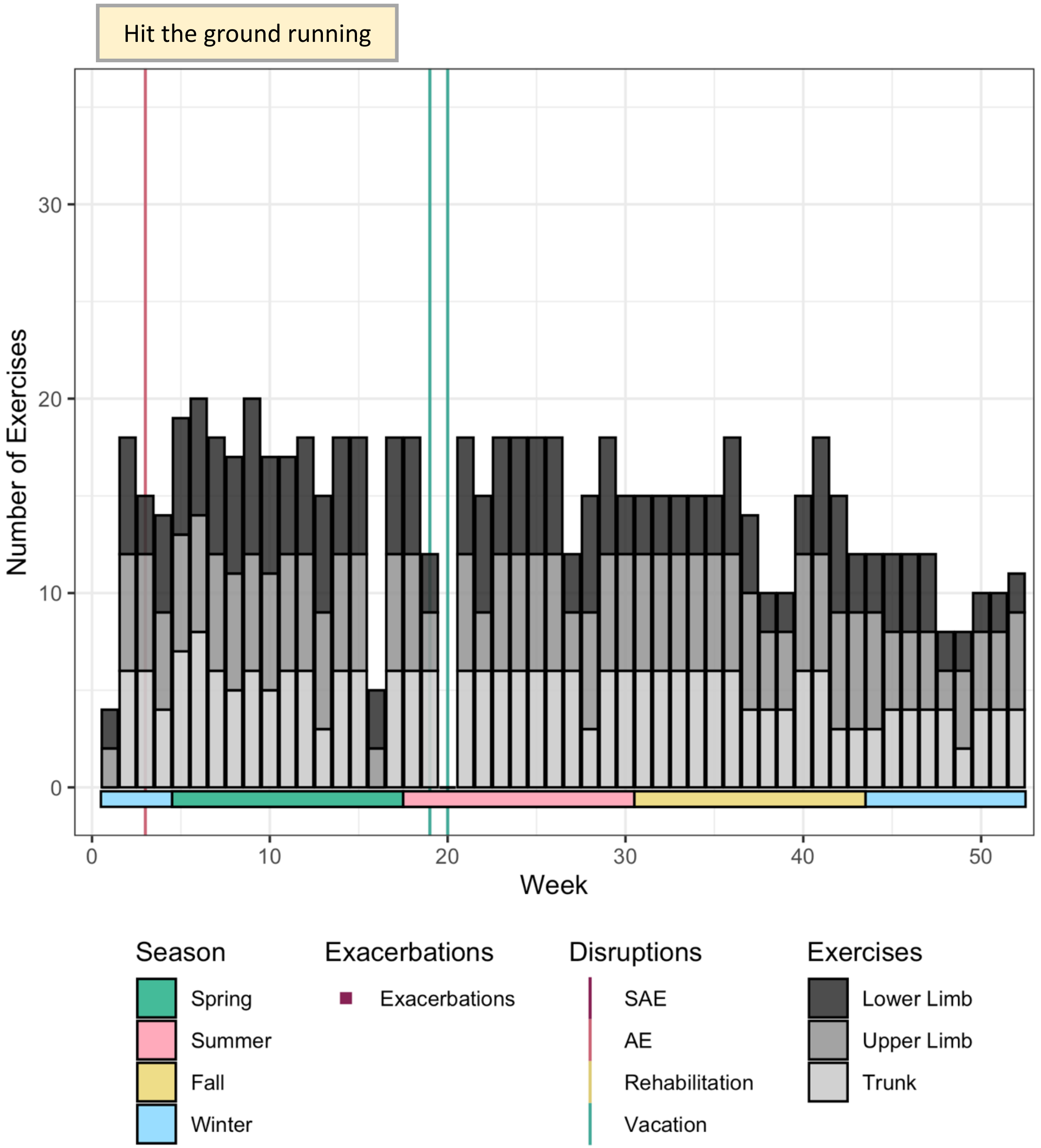

Participant 32

Adherence and Baseline Demographics

**Adherence: All Weeks (%)**	98.1
**Adherence: Healthy Weeks (%)**	98.1
**Bad Health (Weeks)**	0
**Age**	67
**Sex**	Female
**FEV1 (% Pred)**	47.5
**CRQ Dyspnea**	6.5
**Marital Status**	Married or partnership
**Living Situation**	Lives with partner or children
**Comorbidities (n)**	5
**Sparring Partner**	Yes
**Set 2-Month Goal**	No
**Set 12-Month Goal**	Yes

Self-Efficacy

How confident are you in your ability to…

	3 mo.	6mo.	12 mo.
**Practice Daily**	10	10	10
**Practice Correctly**	10	10	10
**Adjust Intensity**	10	10	10
**Keep an Agenda**	10	10	10

Notes

The participant did not report engaging in physical activity other than HOMEX during the program. She reported experiencing positive effects of training in her daily life and was motivated to train both by her experiences in pulmonary rehabilitation.

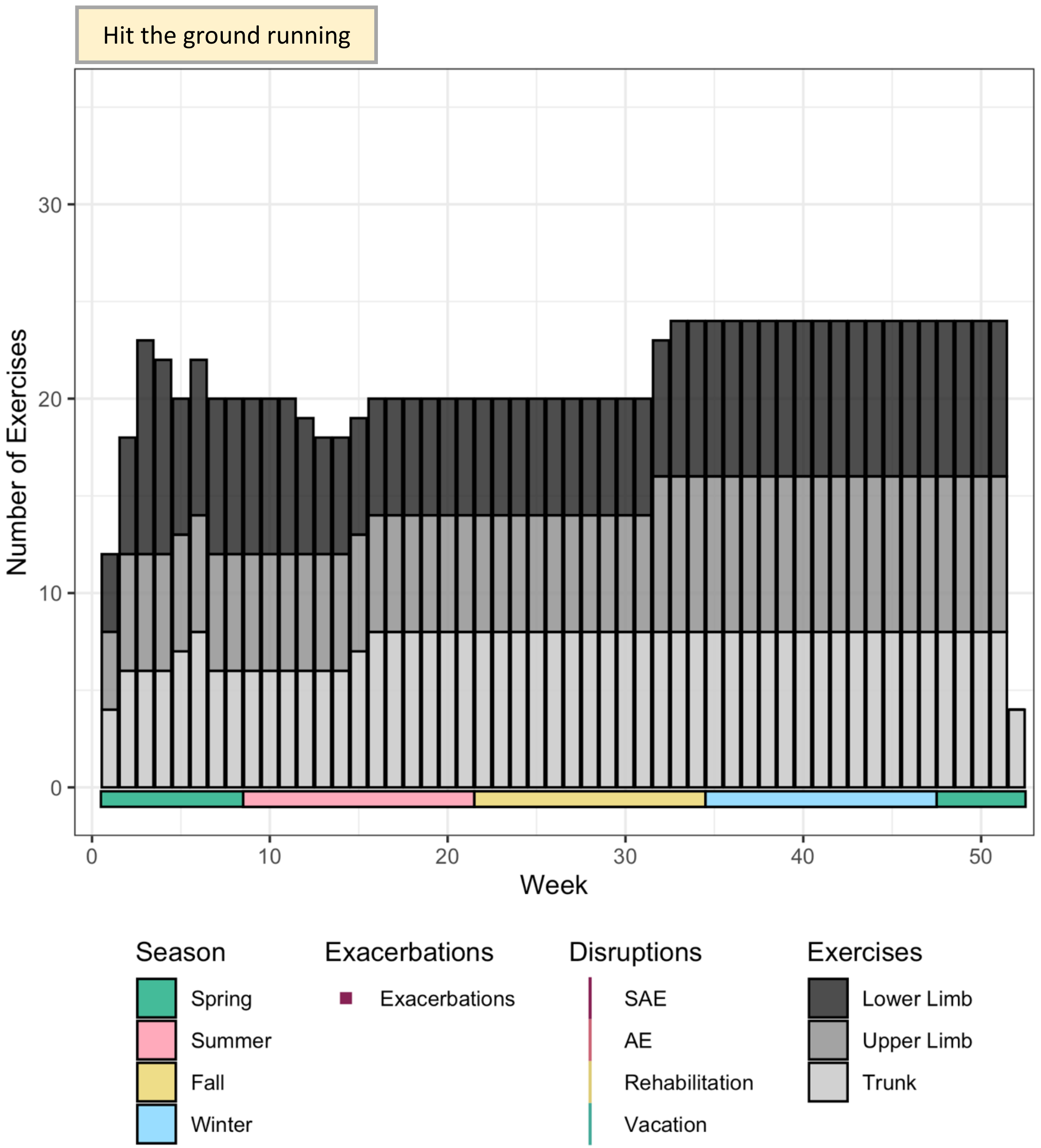

Participant 33

Adherence and Baseline Demographics

**Adherence: All Weeks (%)**	69.2
**Adherence: Healthy Weeks (%)**	94.1
**Bad Health (Weeks)**	18
**Age**	81
**Sex**	Male
**FEV1 (% Pred)**	38
**CRQ Dyspnea**	3.7
**Marital Status**	Widowed
**Living Situation**	Lives alone
**Comorbidities (n)**	6
**Sparring Partner**	Yes
**Set 2-Month Goal**	Yes
**Set 12-Month Goal**	Yes

Self-Efficacy

How confident are you in your ability to…

	3 mo.	6mo.	12 mo.
**Practice Daily**	7	3	3
**Practice Correctly**	8	7	5
**Adjust Intensity**	8	8	9
**Keep an Agenda**	8	9	8

Notes

The participant did not regularly engage in physical activity outside of HOMEX. Though he re-started training following a severe exacerbation, he was eventually forced to stop due to a worsening health condition. He reported experiencing positive effects of training in his daily life and was motivated to continue training to increase his health and fitness.

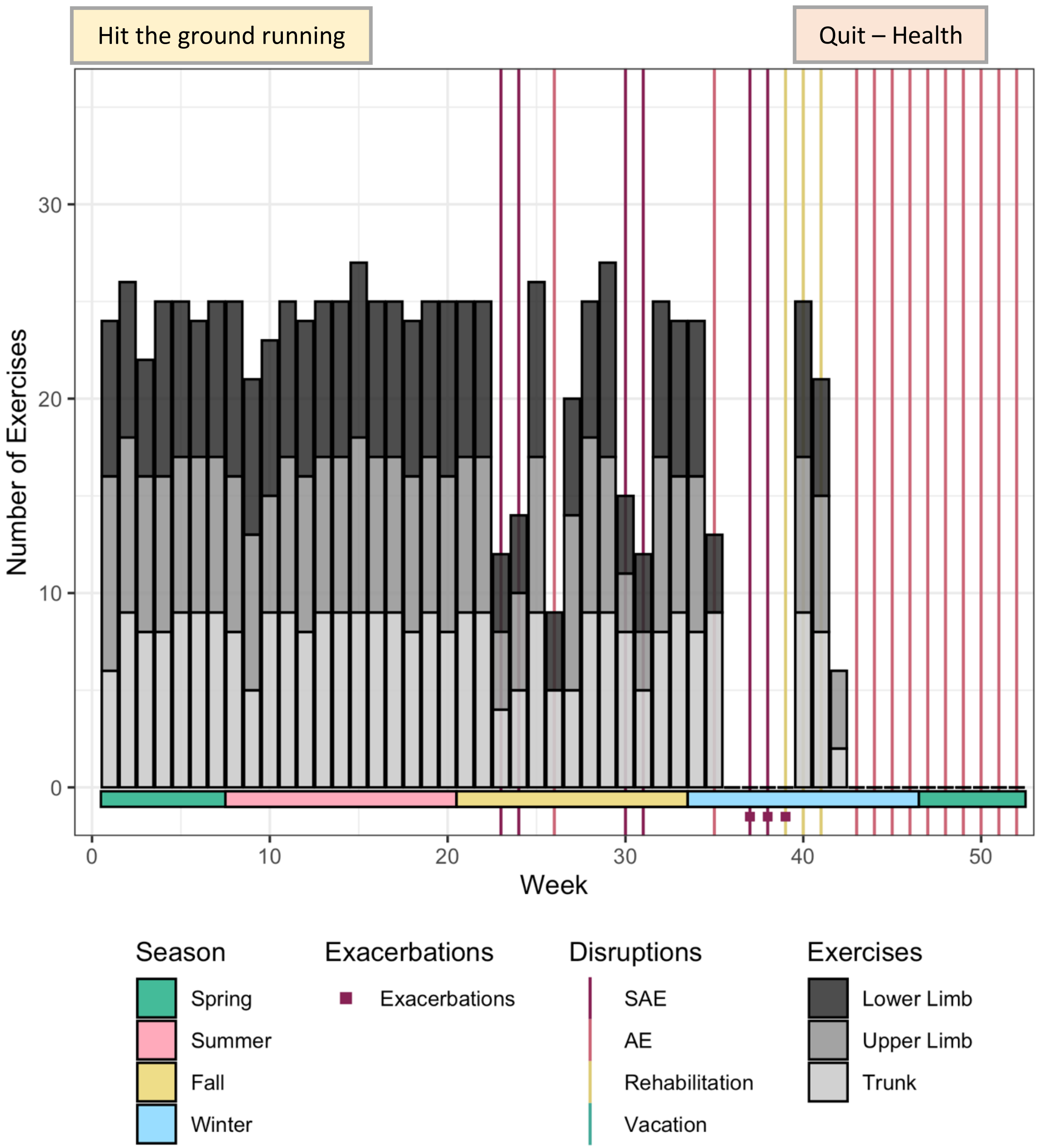

Participant 34

Adherence and Baseline Demographics

**Adherence: All Weeks (%)**	100
**Adherence: Healthy Weeks (%)**	100
**Bad Health (Weeks)**	0
**Age**	57
**Sex**	Male
**FEV1 (% Pred)**	24.9
**CRQ Dyspnea**	4
**Marital Status**	Divorced or separated
**Living Situation**	Lives alone
**Comorbidities (n)**	3
**Sparring Partner**	Yes
**Set 2-Month Goal**	Yes
**Set 12-Month Goal**	Yes

Self-Efficacy

How confident are you in your ability to…

	3 mo.	6mo.	12 mo.
**Practice Daily**	-	9	9
**Practice Correctly**	-	9	9
**Adjust Intensity**	-	10	9
**Keep an Agenda**	-	10	9

Notes

This participant did not report engaging in physical activity other than HOMEX. She experienced the positive effects of training in her daily life and was motivated to train to achieve greater health and fitness.

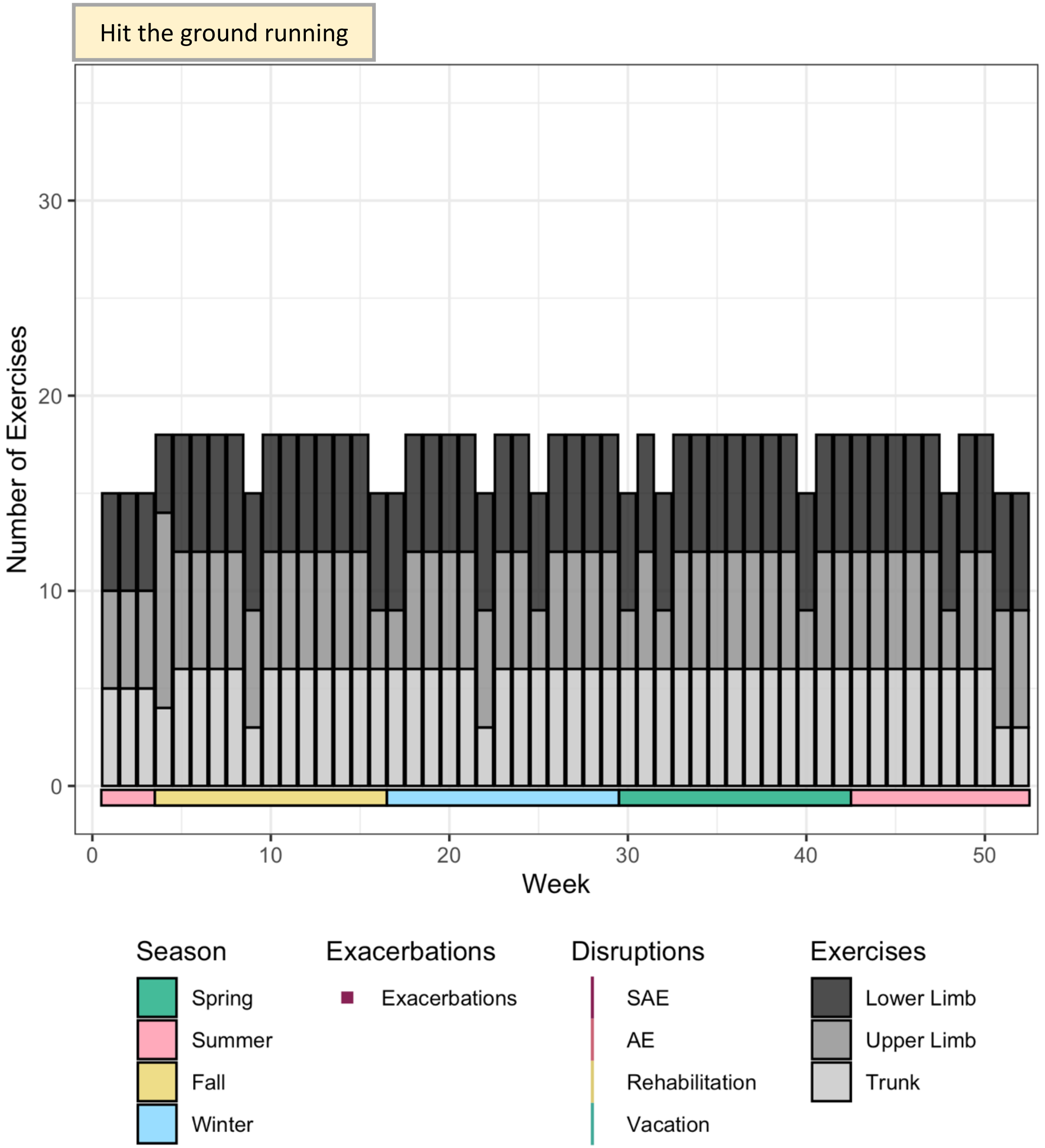

Participant 35

Adherence and Baseline Demographics

**Adherence: All Weeks (%)**	75
**Adherence: Healthy Weeks (%)**	86.7
**Bad Health (Weeks)**	7
**Age**	68
**Sex**	Male
**FEV1 (% Pred)**	25.1
**CRQ Dyspnea**	4.2
**Marital Status**	Married or partnership
**Living Situation**	Lives with partner or children
**Comorbidities (n)**	3
**Sparring Partner**	Yes
**Set 2-Month Goal**	Yes
**Set 12-Month Goal**	Yes

Self-Efficacy

How confident are you in your ability to…

	3 mo.	6mo.	12 mo.
**Practice Daily**	7	9	10
**Practice Correctly**	8	8	10
**Adjust Intensity**	8	9	9
**Keep an Agenda**	8	9	10

Notes

The participant trained only lightly (without documenting training) at the beginning of the program due to stress. However, he re-started training during rehabilitation and remained adherent thereafter. He did not participate in exit interviews because he died shortly after completing the study.

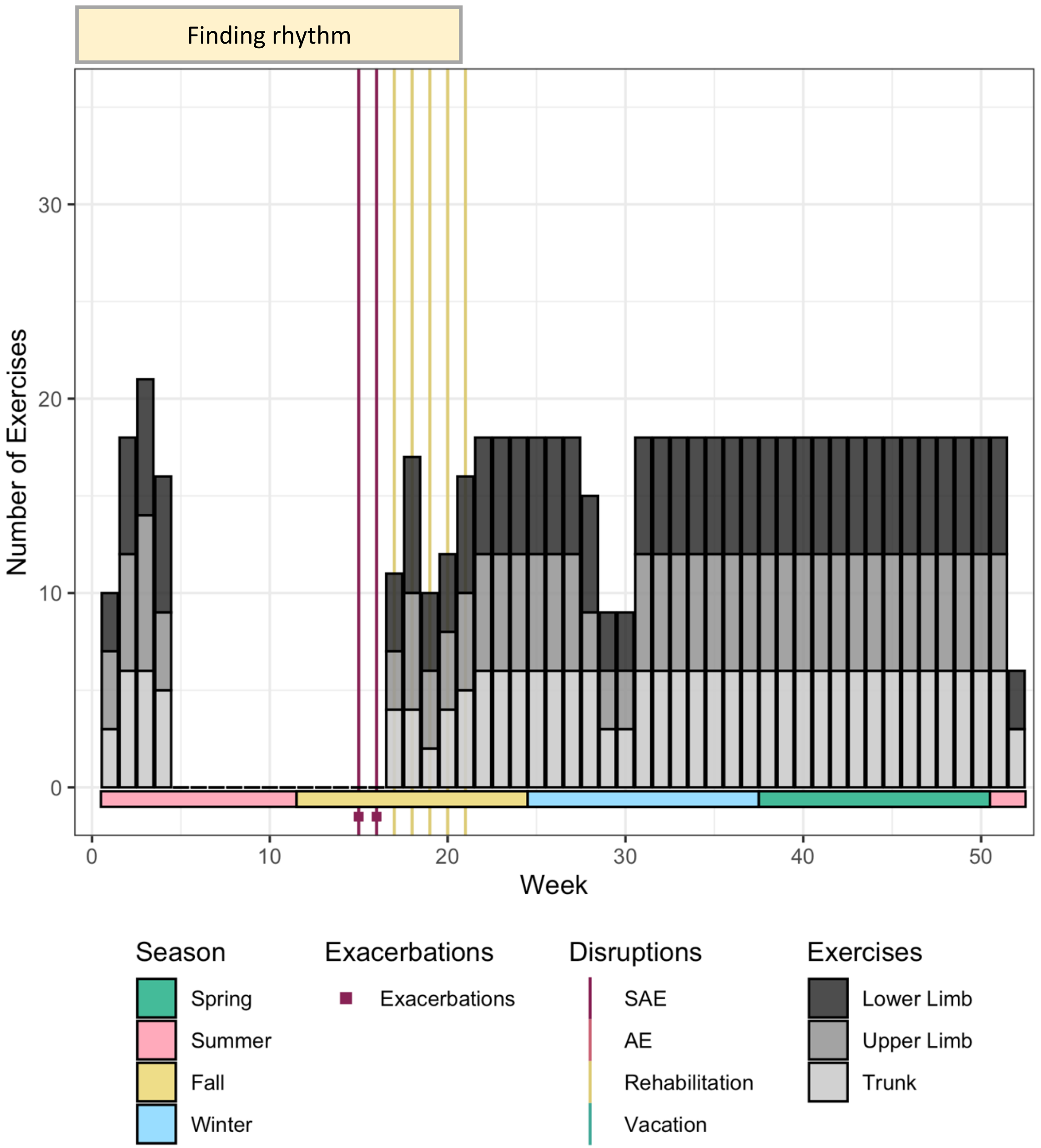

Participant 36

Adherence and Baseline Demographics

**Adherence: All Weeks (%)**	96.2
**Adherence: Healthy Weeks (%)**	98
**Bad Health (Weeks)**	3
**Age**	78
**Sex**	Female
**FEV1 (% Pred)**	30.2
**CRQ Dyspnea**	3.4
**Marital Status**	Divorced or separated
**Living Situation**	Lives alone
**Comorbidities (n)**	2
**Sparring Partner**	Yes
**Set 2-Month Goal**	No
**Set 12-Month Goal**	No

Self-Efficacy

How confident are you in your ability to…

	**3 mo.**	**6mo.**	**12 mo.**
**Practice Daily**	10	9	10
**Practice Correctly**	10	8	8
**Adjust Intensity**	10	8	6
**Keep an Agenda**	10	9	6

Notes

This participant started one week late due to heart problems, therefore only 51 weeks are recorded. She did not participate in the exit interviews.

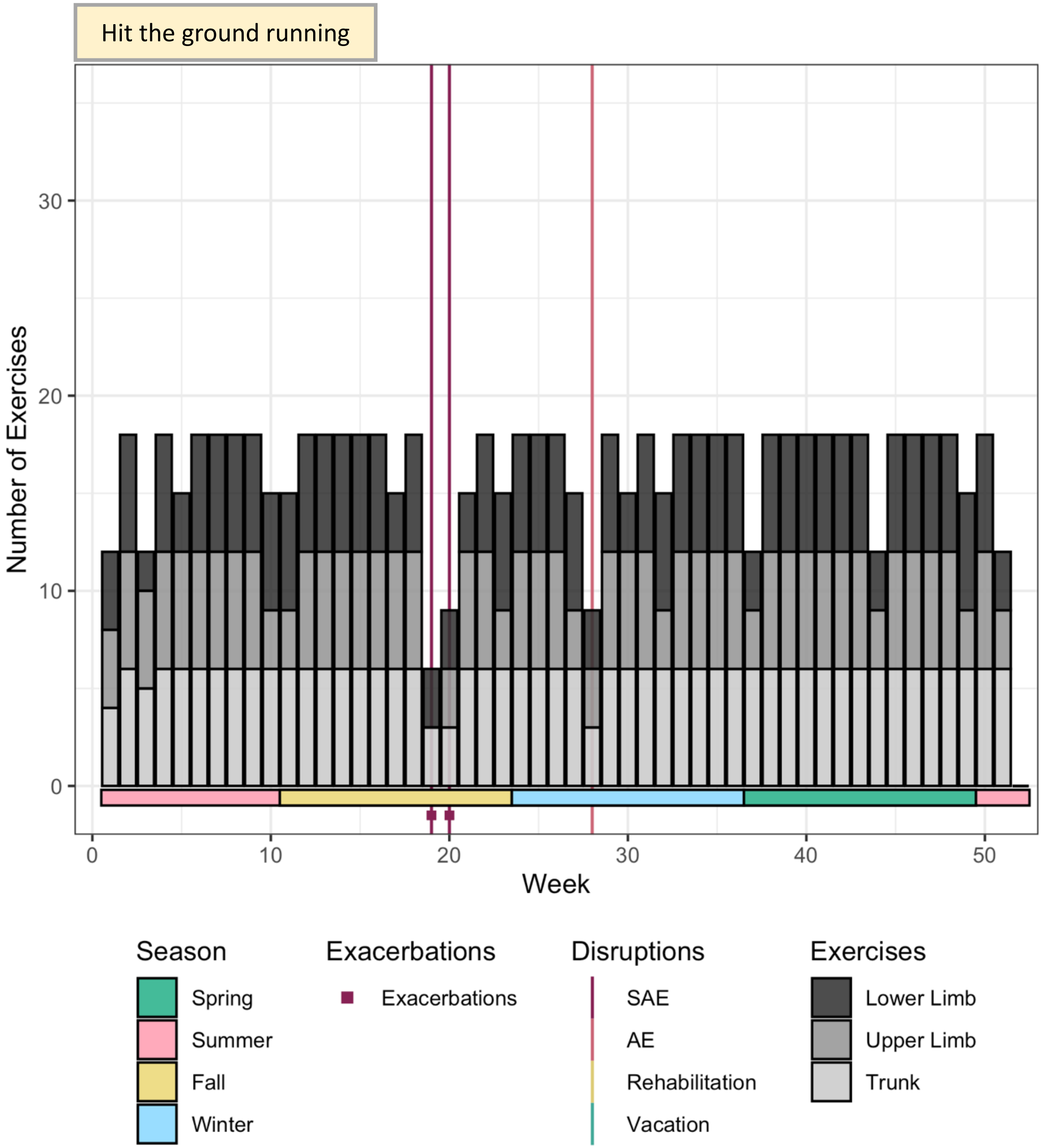

Participant 37

Adherence and Baseline Demographics

**Adherence: All Weeks (%)**	57.7
**Adherence: Healthy Weeks (%)**	60
**Bad Health (Weeks)**	2
**Age**	68
**Sex**	Female
**FEV1 (% Pred)**	44.7
**CRQ Dyspnea**	6.5
**Marital Status**	Married or partnership
**Living Situation**	Lives with partner or children
**Comorbidities (n)**	4
**Sparring Partner**	Yes
**Set 2-Month Goal**	Yes
**Set 12-Month Goal**	Yes

Self-Efficacy

How confident are you in your ability to…

	3 mo.	6mo.	12 mo.
**Practice Daily**	10	10	6
**Practice Correctly**	9	10	10
**Adjust Intensity**	10	8	10
**Keep an Agenda**	10	9	10

Notes

The participant did not report engaging in physical activity other than HOMEX throughout the year. She reported worsening dyspnea and other health conditions prior to stopping HOMEX training at week 35. She reported that she felt positive effects of training and was motivated by her coach.

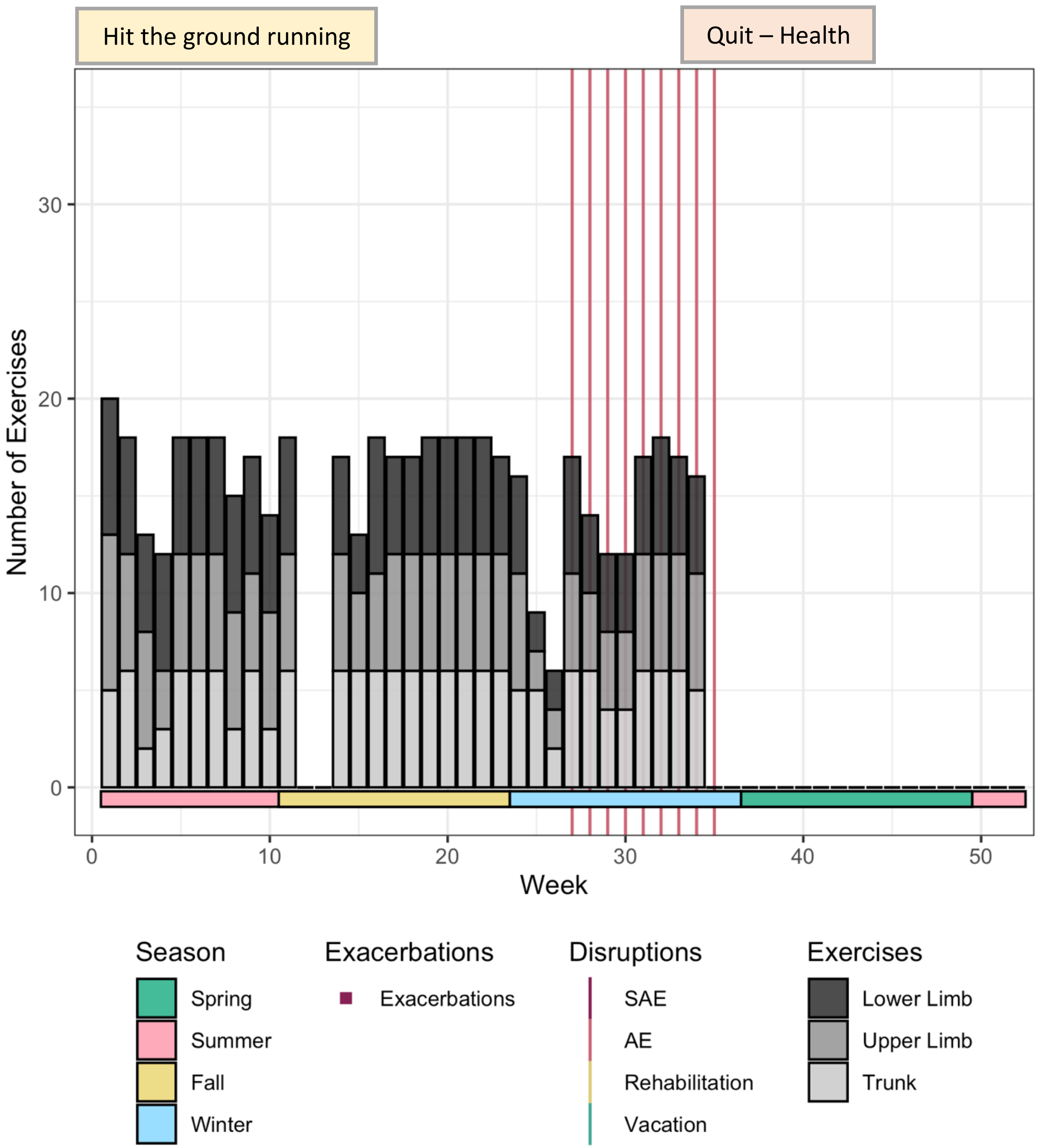

Participant 38

Adherence and Baseline Demographics

**Adherence: All Weeks (%)**	82.7
**Adherence: Healthy Weeks (%)**	84.3
**Bad Health (Weeks)**	1
**Age**	73
**Sex**	Female
**FEV1 (% Pred)**	65.5
**CRQ Dyspnea**	6.7
**Marital Status**	Widowed
**Living Situation**	Lives alone
**Comorbidities (n)**	8
**Sparring Partner**	Yes
**Set 2-Month Goal**	Yes
**Set 12-Month Goal**	Yes

Self-Efficacy

How confident are you in your ability to…

	3 mo.	6mo.	12 mo.
**Practice Daily**	10	10	10
**Practice Correctly**	9	8	9
**Adjust Intensity**	10	10	10
**Keep an Agenda**	10	10	10

Notes

This participant did not regularly report engaging in physical activity other than HOMEX. She experienced the positive effects of training in her daily life and was motivated to train to achieve greater health and fitness.

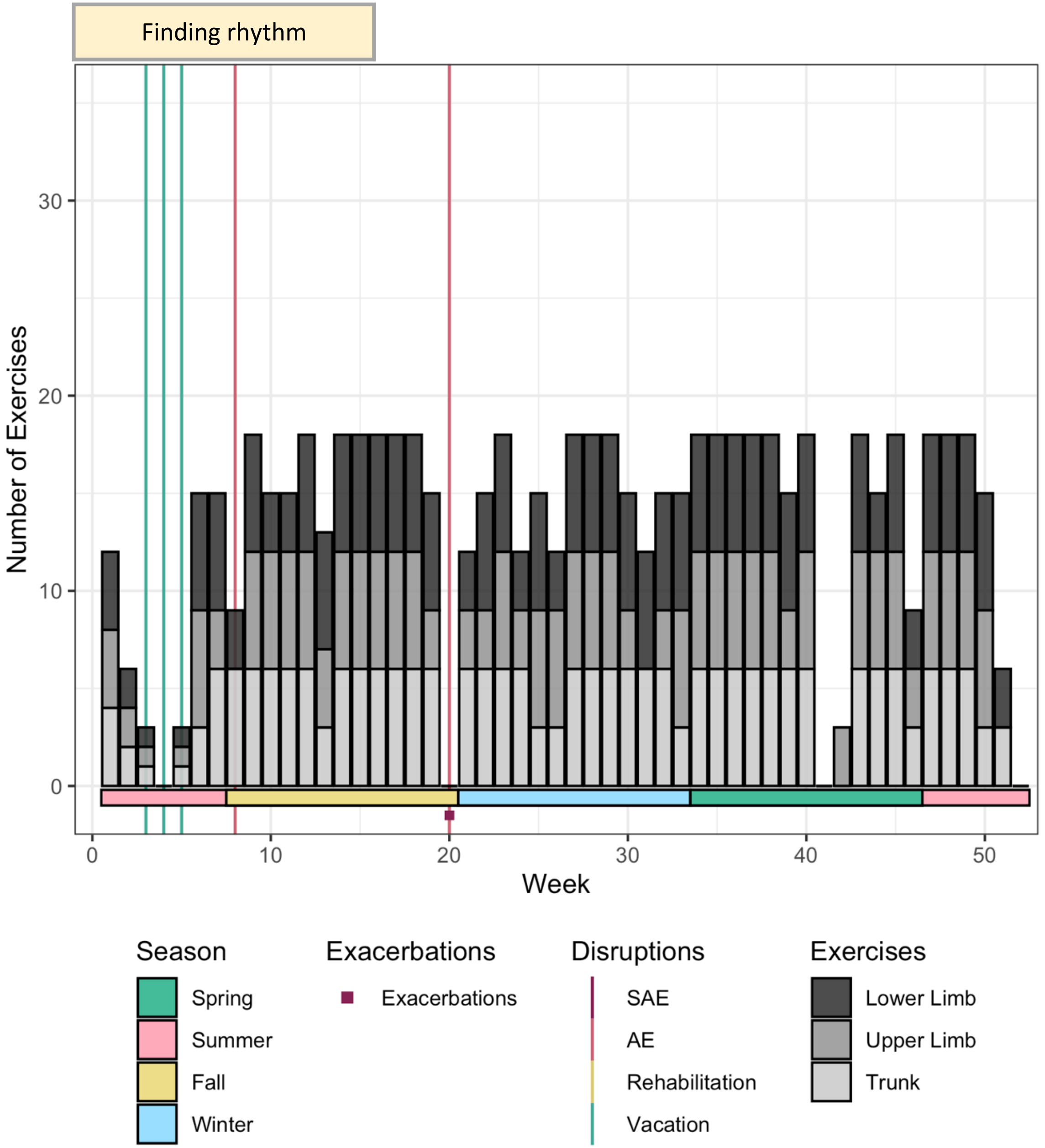

Participant 39

Adherence and Baseline Demographics

**Adherence: All Weeks (%)**	98.1
**Adherence: Healthy Weeks (%)**	100
**Bad Health (Weeks)**	1
**Age**	70
**Sex**	Male
**FEV1 (% Pred)**	37
**CRQ Dyspnea**	4.2
**Marital Status**	Married or partnership
**Living Situation**	Lives with partner or children
**Comorbidities (n)**	3
**Sparring Partner**	Yes
**Set 2-Month Goal**	No
**Set 12-Month Goal**	No

Self-Efficacy

How confident are you in your ability to…

	3 mo.	6mo.	12 mo.
**Practice Daily**	10	10	10
**Practice Correctly**	10	10	10
**Adjust Intensity**	10	10	10
**Keep an Agenda**	10	10	10

Notes

This participant regularly reported engaging in physical activity other than HOMEX. He experienced the positive effects of training in his daily life and was motivated to train because of his experiences in pulmonary rehabilitation.

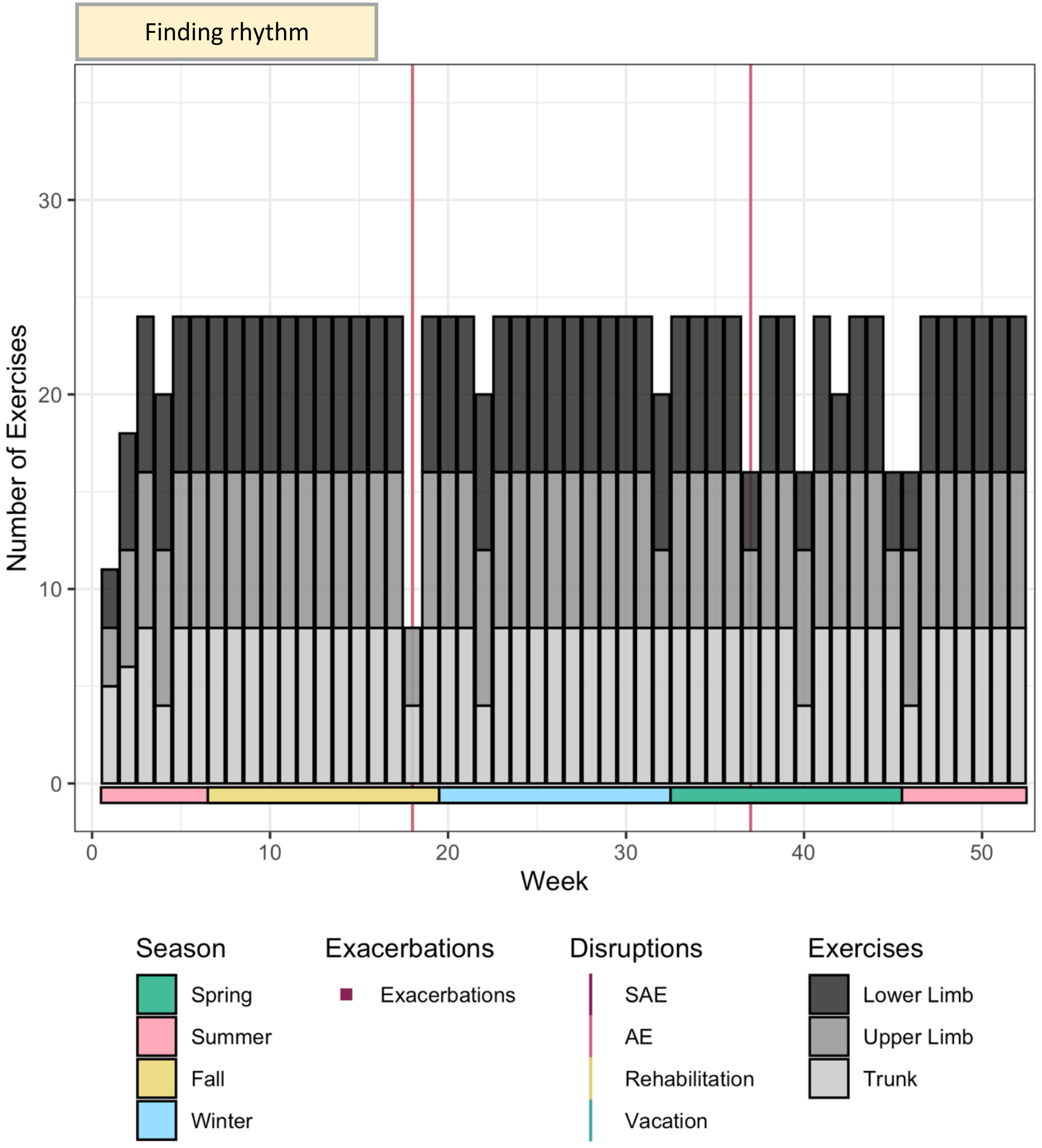

Participant 40

Adherence and Baseline Demographics

**Adherence: All Weeks (%)**	67.3
**Adherence: Healthy Weeks (%)**	94.4
**Bad Health (Weeks)**	16
**Age**	59
**Sex**	Female
**FEV1 (% Pred)**	35.4
**CRQ Dyspnea**	4.8
**Marital Status**	Married or partnership
**Living Situation**	Lives with partner or children
**Comorbidities (n)**	2
**Sparring Partner**	Yes
**Set 2-Month Goal**	Yes
**Set 12-Month Goal**	Yes

Self-Efficacy

How confident are you in your ability to…

	3 mo.	6mo.	12 mo.
**Practice Daily**	9	9	10
**Practice Correctly**	7	9	10
**Adjust Intensity**	9	8	10
**Keep an Agenda**	10	9	10

Notes

This participant did not report engaging in physical activity other than HOMEX. She reduced and then stopped training due to worsening dyspnea and the effects of cortisone following an exacerbation. She experienced the positive effects of training in her daily life and was motivated to train both by her coach and because of her experiences in rehabilitation.

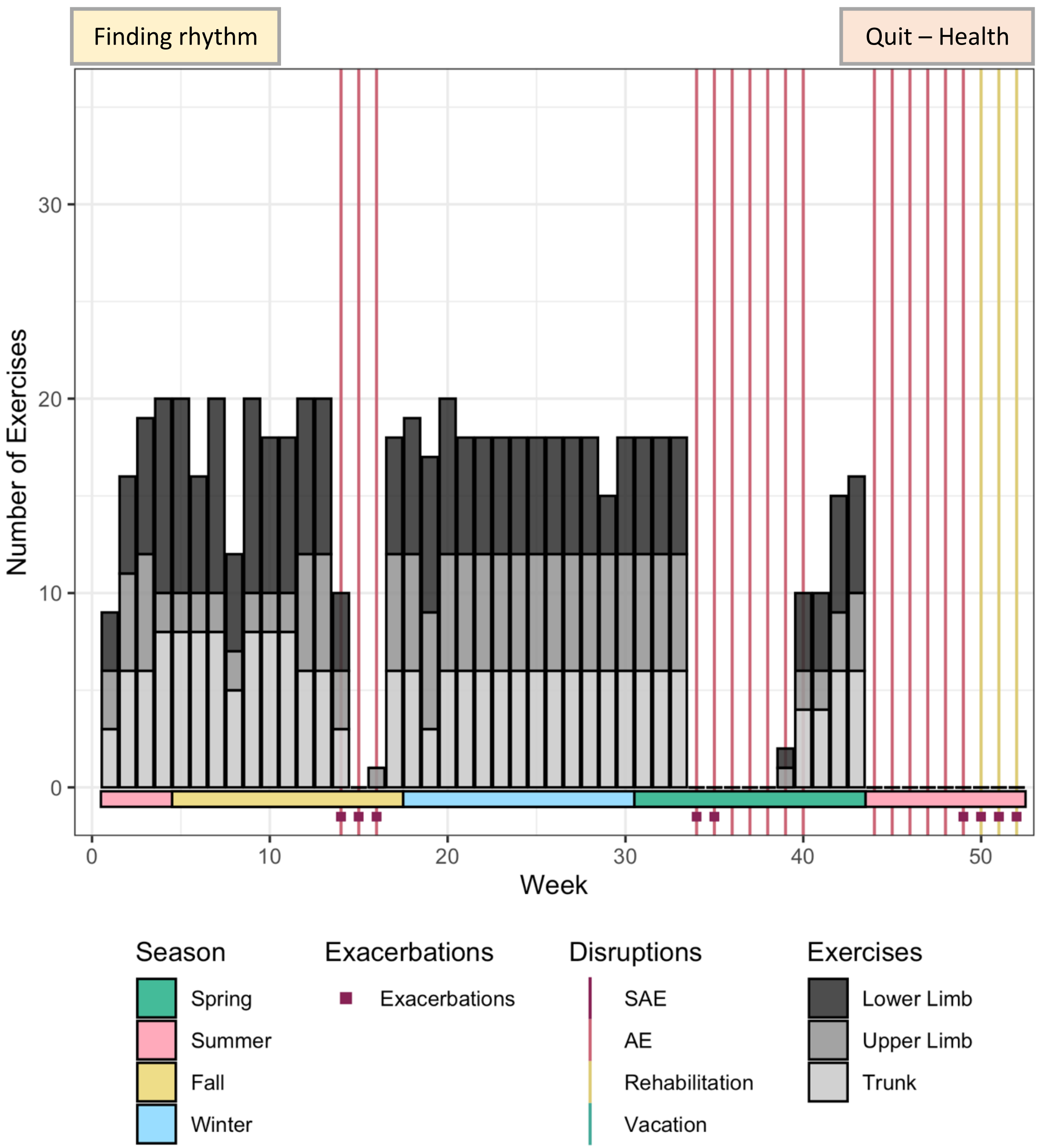

Participant 41

Adherence and Baseline Demographics

**Adherence: All Weeks (%)**	50
**Adherence: Healthy Weeks (%)**	55.3
**Bad Health (Weeks)**	5
**Age**	70
**Sex**	Male
**FEV1 (% Pred)**	54.9
**CRQ Dyspnea**	6.5
**Marital Status**	Married or partnership
**Living Situation**	Lives with partner or children
**Comorbidities (n)**	2
**Sparring Partner**	Yes
**Set 2-Month Goal**	Yes
**Set 12-Month Goal**	Yes

Self-Efficacy

How confident are you in your ability to…

	3 mo.	6mo.	12 mo.
**Practice Daily**	10	9	2
**Practice Correctly**	10	10	7
**Adjust Intensity**	10	10	10
**Keep an Agenda**	10	9	2

Notes

This participant did not report engaging in physical activity other than HOMEX. He reduced and then stopped training because he was experiencing pain. He experienced the positive effects of training in his daily life.

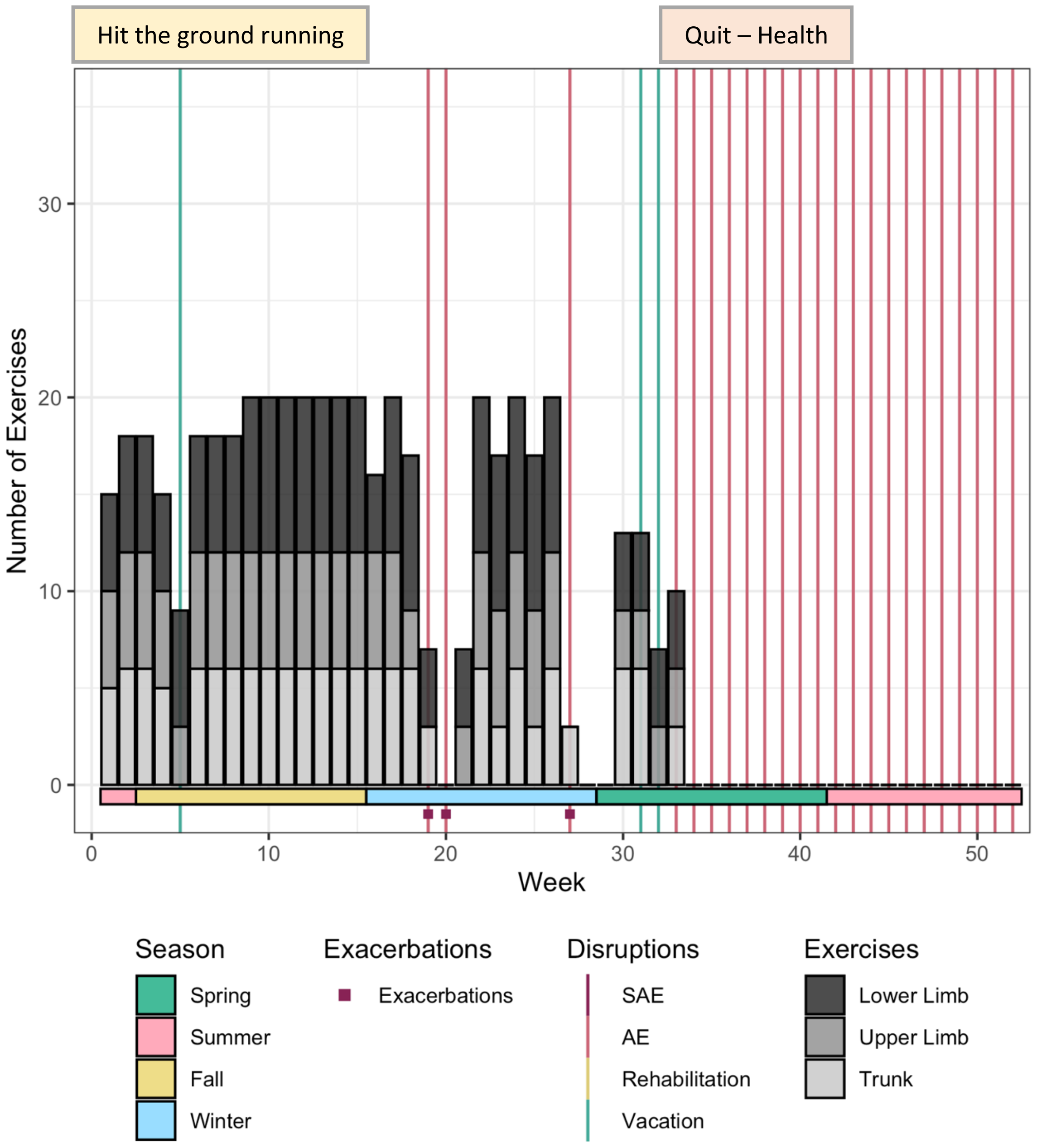

Participant 42

Adherence and Baseline Demographics

**Adherence: All Weeks (%)**	100
**Adherence: Healthy Weeks (%)**	100
**Bad Health (Weeks)**	1
**Age**	54
**Sex**	Male
**FEV1 (% Pred)**	36
**CRQ Dyspnea**	3
**Marital Status**	Married or partnership
**Living Situation**	Lives alone
**Comorbidities (n)**	2
**Sparring Partner**	No
**Set 2-Month Goal**	No
**Set 12-Month Goal**	No

Self-Efficacy

How confident are you in your ability to…

	**3 mo.**	**6mo.**	**12 mo.**
**Practice Daily**	10	10	9
**Practice Correctly**	7	7	8
**Adjust Intensity**	8	8	8
**Keep an Agenda**	10	9	8

Notes

This participant did not report engaging in physical activity other than HOMEX. He experienced the positive effects of training in his daily life and was motivated to train to achieve greater health and fitness.

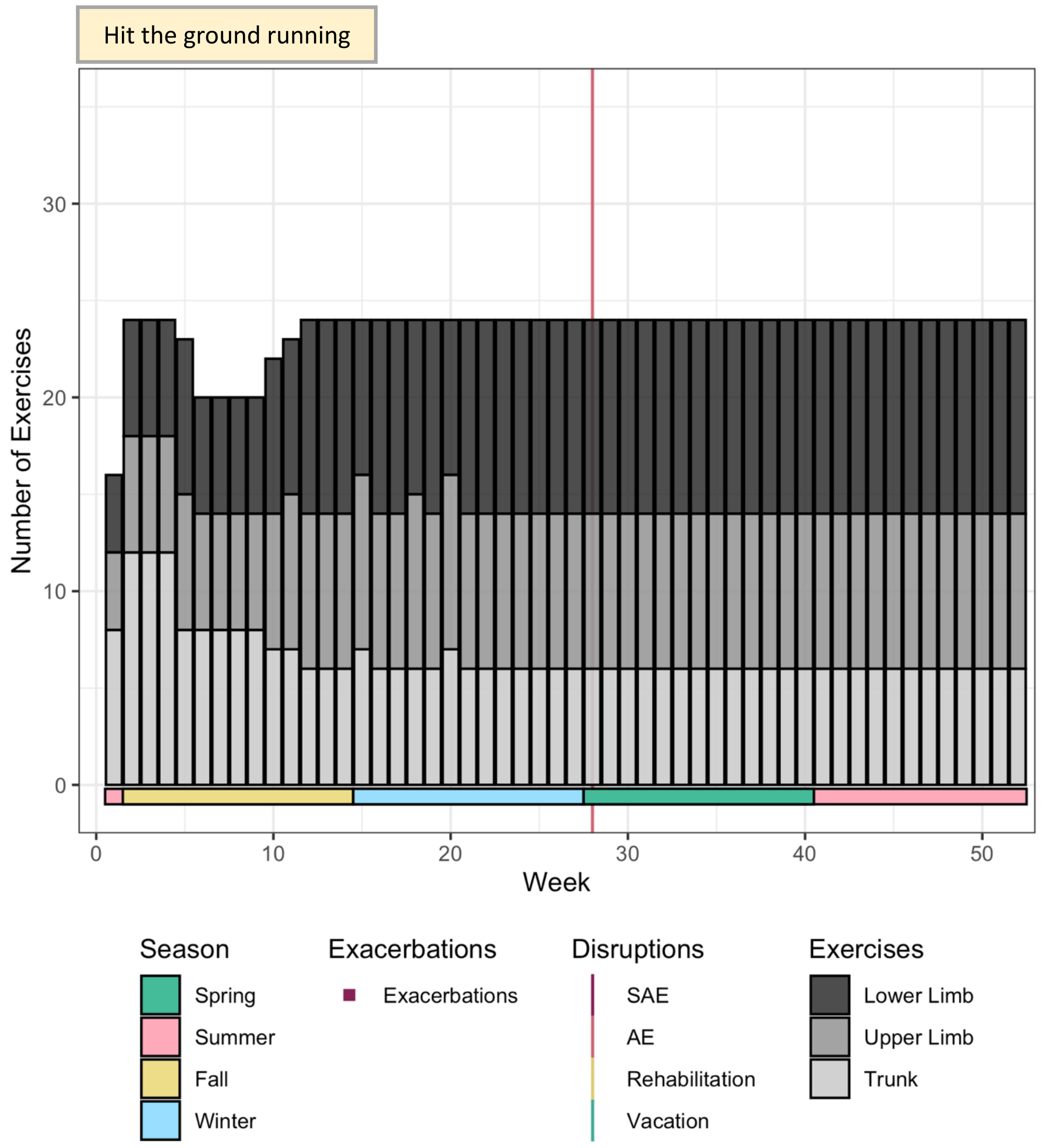

Participant 43

Adherence and Baseline Demographics

**Adherence: All Weeks (%)**	92.3
**Adherence: Healthy Weeks (%)**	93.9
**Bad Health (Weeks)**	3
**Age**	68
**Sex**	Female
**FEV1 (% Pred)**	34.2
**CRQ Dyspnea**	5.2
**Marital Status**	Divorced or separated
**Living Situation**	Lives alone
**Comorbidities (n)**	1
**Sparring Partner**	Yes
**Set 2-Month Goal**	Yes
**Set 12-Month Goal**	Yes

Self-Efficacy

How confident are you in your ability to…

	3 mo.	6mo.	12 mo.
**Practice Daily**	10	10	10
**Practice Correctly**	10	10	10
**Adjust Intensity**	10	10	10
**Keep an Agenda**	10	10	10

Notes

This participant regularly reported engaging in physical activity other than HOMEX. She did not experience positive effects and reported that despite training her oxygen saturation became worse over time.

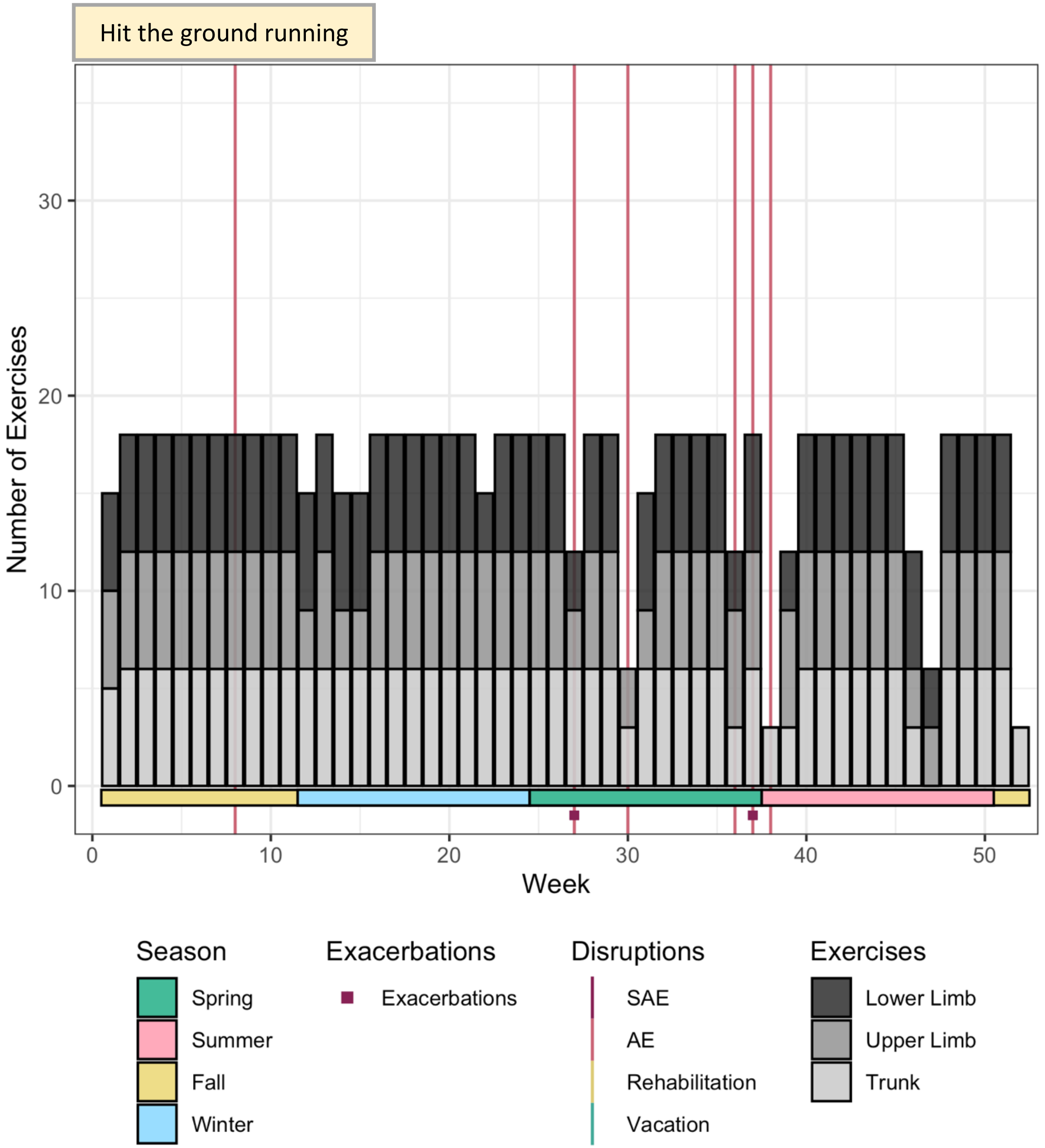

Participant 44

Adherence and Baseline Demographics

**Adherence: All Weeks (%)**	76.9
**Adherence: Healthy Weeks (%)**	97.4
**Bad Health (Weeks)**	13
**Age**	73
**Sex**	Female
**FEV1 (% Pred)**	50.2
**CRQ Dyspnea**	4.7
**Marital Status**	Widowed
**Living Situation**	Lives alone
**Comorbidities (n)**	8
**Sparring Partner**	Yes
**Set 2-Month Goal**	Yes
**Set 12-Month Goal**	Yes

Self-Efficacy

How confident are you in your ability to…

	**3 mo.**	**6mo.**	**12 mo.**
**Practice Daily**	10	8	8
**Practice Correctly**	10	9	10
**Adjust Intensity**	10	9	10
**Keep an Agenda**	10	10	4

Notes

This participant did not engage in physical activity other than HOMEX. She did not experience positive effects in her daily life and reported she still had breathing difficulties despite training. She was originally motivated to train to increase her health and fitness.

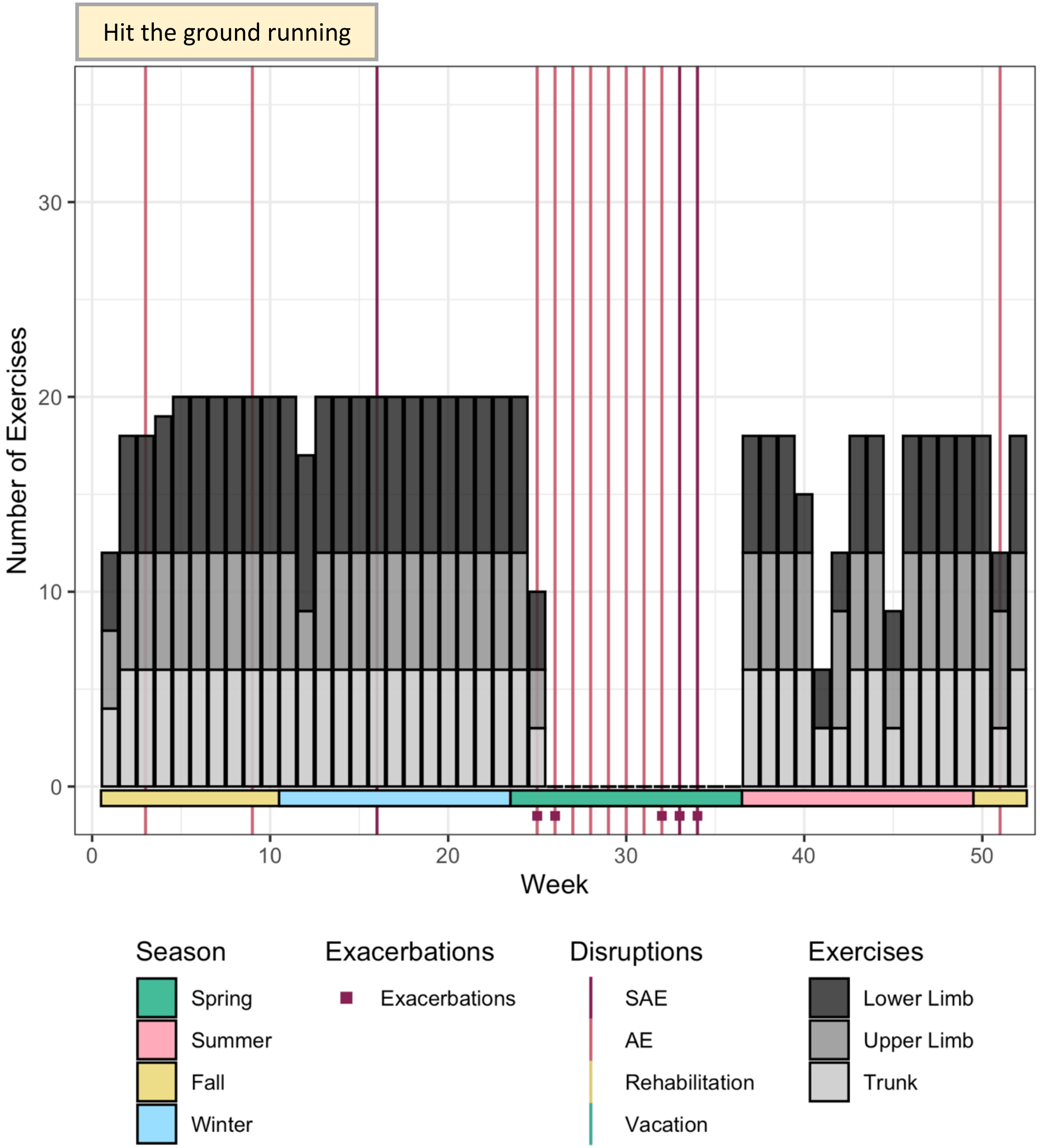

Participant 45

Adherence and Baseline Demographics

**Adherence: All Weeks (%)**	94.2
**Adherence: Healthy Weeks (%)**	97.8
**Bad Health (Weeks)**	6
**Age**	69
**Sex**	Male
**FEV1 (% Pred)**	25.8
**CRQ Dyspnea**	4.3
**Marital Status**	Married or partnership
**Living Situation**	Lives with partner or children
**Comorbidities (n)**	3
**Sparring Partner**	Yes
**Set 2-Month Goal**	Yes
**Set 12-Month Goal**	Yes

Self-Efficacy

How confident are you in your ability to…

	3 mo.	6mo.	12 mo.
**Practice Daily**	10	10	8
**Practice Correctly**	8	9	10
**Adjust Intensity**	8	9	8
**Keep an Agenda**	10	10	8

Notes

This participant did not report engaging in physical activity other than HOMEX. He experienced the positive effects of training in his daily life and was motivated to train because of his. experiences in pulmonary rehabilitation.

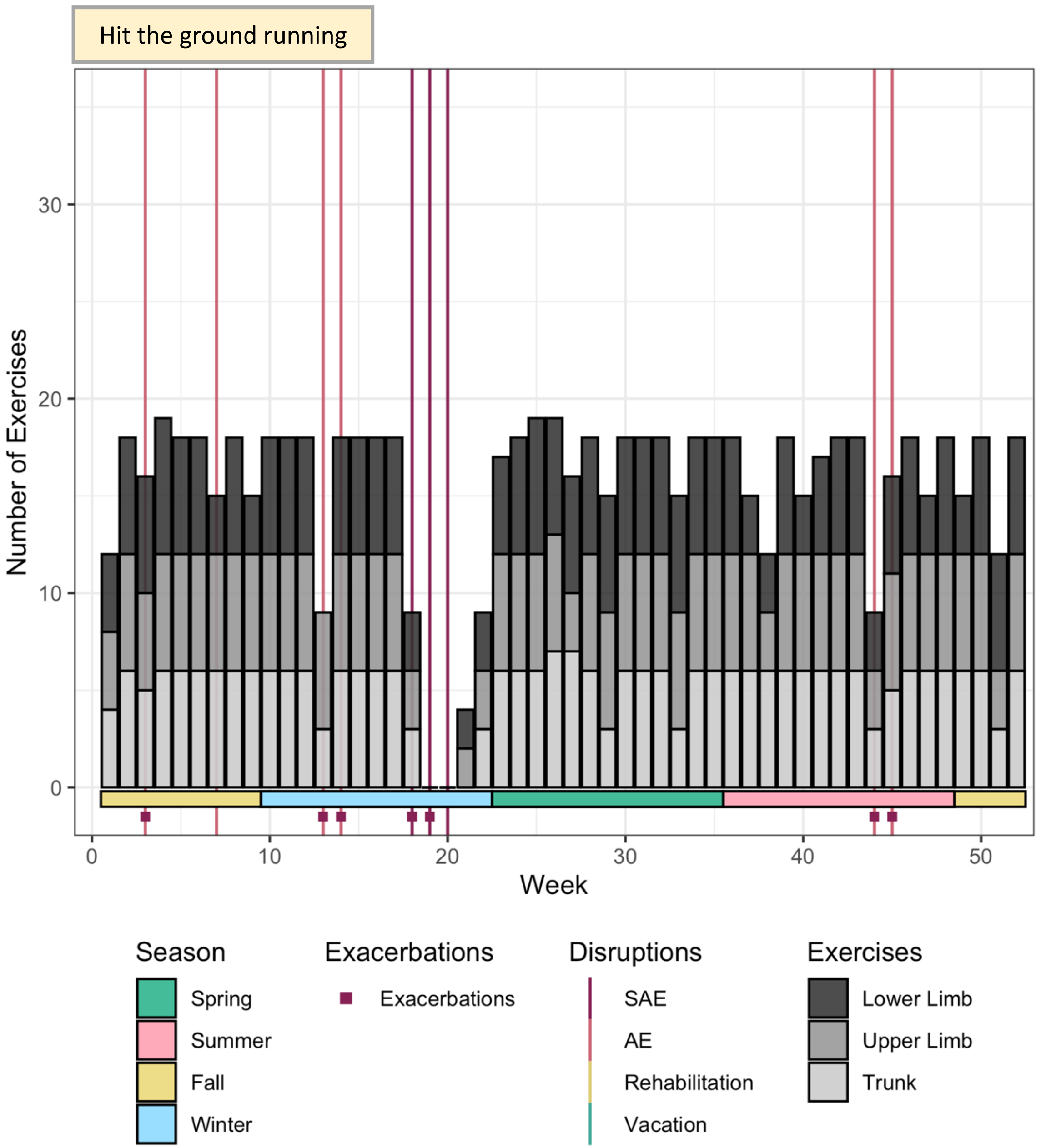

Participant 46

Adherence and Baseline Demographics

**Adherence: All Weeks (%)**	90.4
**Adherence: Healthy Weeks (%)**	100
**Bad Health (Weeks)**	7
**Age**	57
**Sex**	Male
**FEV1 (% Pred)**	73
**CRQ Dyspnea**	5
**Marital Status**	Divorced or separated
**Living Situation**	Lives alone
**Comorbidities (n)**	4
**Sparring Partner**	No
**Set 2-Month Goal**	Yes
**Set 12-Month Goal**	Yes

Self-Efficacy

How confident are you in your ability to...

	3 mo.	6mo.	12 mo.
**Practice Daily**	-	4	9
**Practice Correctly**	-	9	9
**Adjust Intensity**	-	9	9
**Keep an Agenda**	-	4	9

Notes

This participant occasionally reported engaging in physical activity other than HOMEX. He paused training following a motorcycle accident. He experienced the positive effects of training in his daily life and was motivated to train both because of his experiences in pulmonary rehabilitation and to increase his health and fitness.

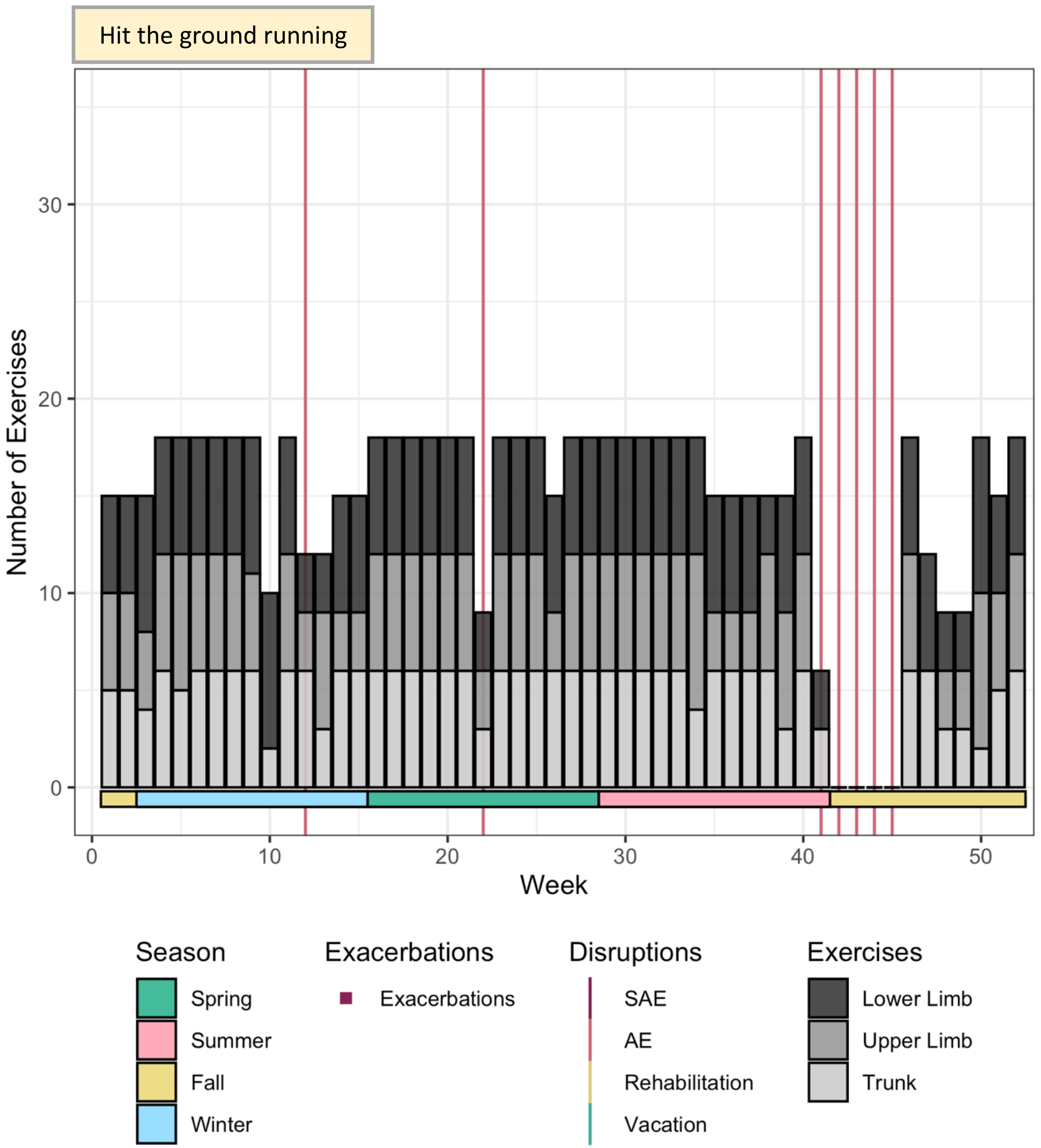

Participant 47

Adherence and Baseline Demographics

**Adherence: All Weeks (%)**	76.9
**Adherence: Healthy Weeks (%)**	76.9
**Bad Health (Weeks)**	0
**Age**	65
**Sex**	Male
**FEV1 (% Pred)**	41.9
**CRQ Dyspnea**	4.5
**Marital Status**	Married or partnership
**Living Situation**	Lives with partner or children
**Comorbidities (n)**	6
**Sparring Partner**	Yes
**Set 2-Month Goal**	Yes
**Set 12-Month Goal**	Yes

Self-Efficacy

How confident are you in your ability to…

	3 mo.	6mo.	12 mo.
**Practice Daily**	7	7	6
**Practice Correctly**	6	7	6
**Adjust Intensity**	7	7	6
**Keep an Agenda**	7	7	6

Notes

This participant occasionally reported engaging in physical activity other than HOMEX. He experienced the positive effects of training in his daily life and was motivated to train both because of his experiences in pulmonary rehabilitation.

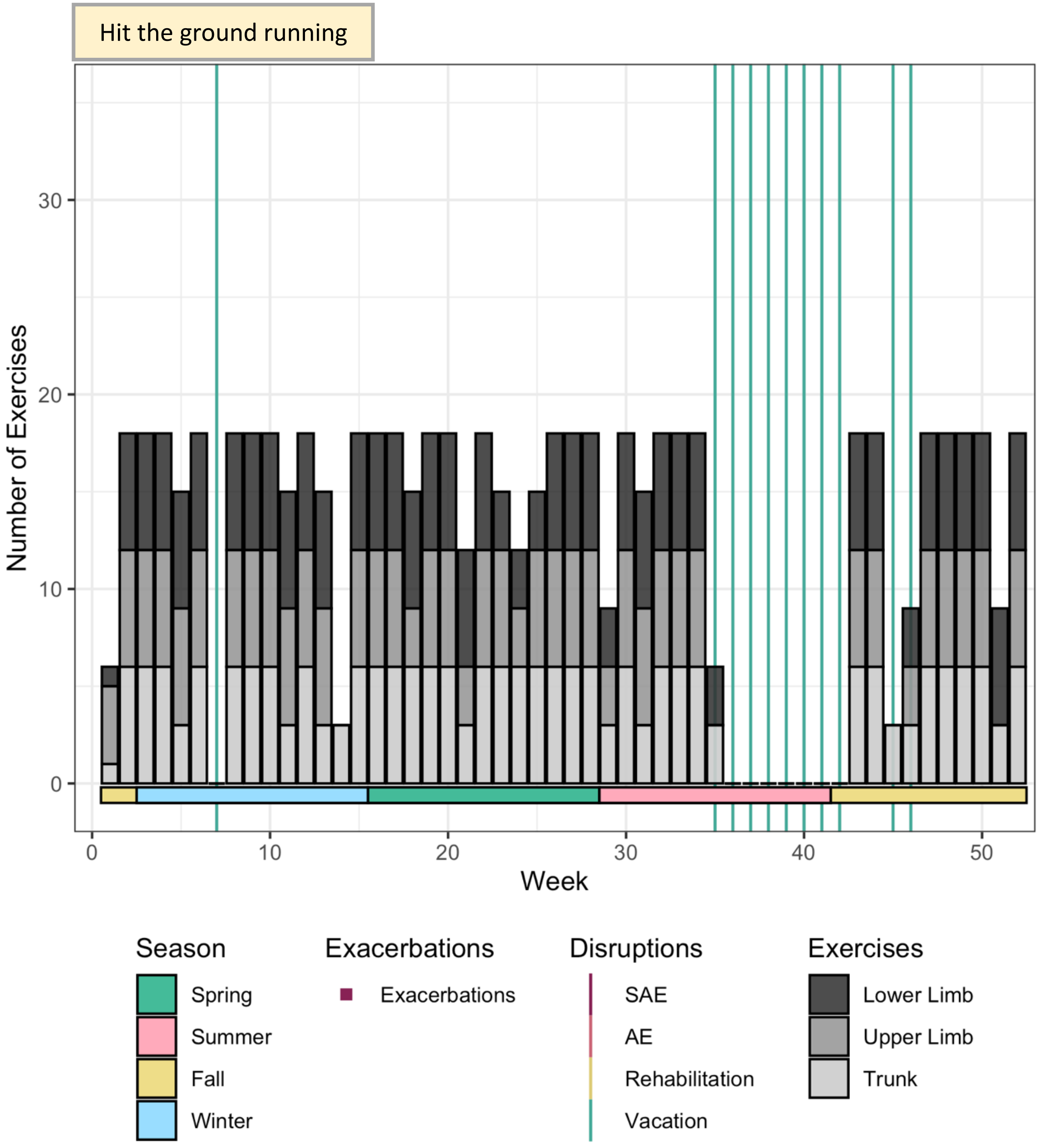

Participant 48

Adherence and Baseline Demographics

**Adherence: All Weeks (%)**	15.4
**Adherence: Healthy Weeks (%)**	19
**Bad Health (Weeks)**	10
**Age**	54
**Sex**	Male
**FEV1 (% Pred)**	27.1
**CRQ Dyspnea**	3.5
**Marital Status**	Divorced or separated
**Living Situation**	Lives alone
**Comorbidities (n)**	5
**Sparring Partner**	Yes
**Set 2-Month Goal**	Yes
**Set 12-Month Goal**	Yes

Self-Efficacy

How confident are you in your ability to…

	3 mo.	6mo.	12 mo.
**Practice Daily**	5	5	5
**Practice Correctly**	4	5	4
**Adjust Intensity**	4	5	5
**Keep an Agenda**	4	5	4

Notes

This participant began physical activity following his initial illness, coinciding with a decrease in HOMEX training. He experienced the positive effects of training at first, but then experienced additional illnesses had to reduce all training after week 30. He was motivated to train because he set a concrete goal.

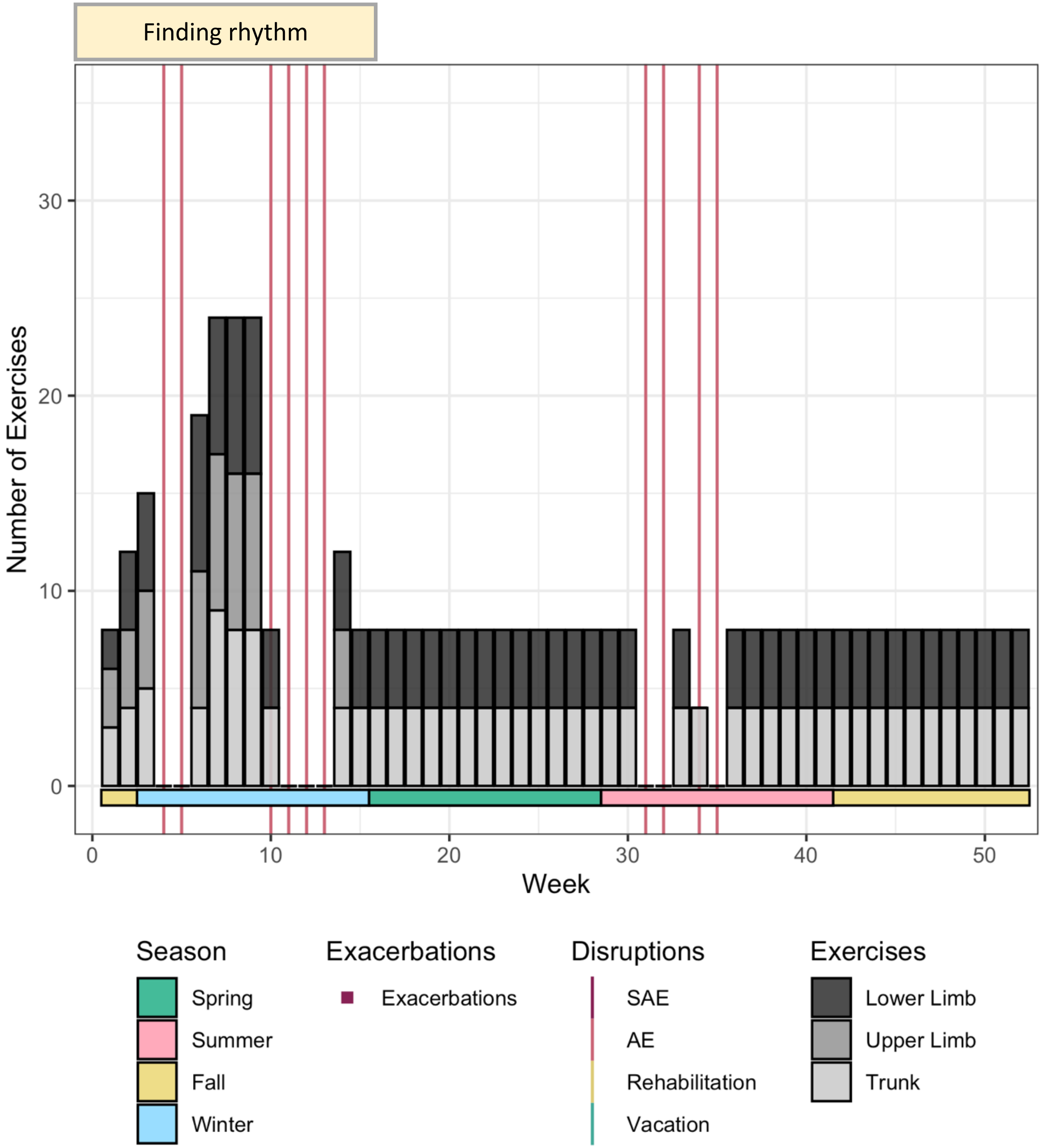

Participant 49

Adherence and Baseline Demographics

**Adherence: All Weeks (%)**	42.3
**Adherence: Healthy Weeks (%)**	50
**Bad Health (Weeks)**	14
**Age**	83
**Sex**	Male
**FEV1 (% Pred)**	41.6
**CRQ Dyspnea**	6.8
**Marital Status**	Married or partnership
**Living Situation**	Lives with partner or children
**Comorbidities (n)**	4
**Sparring Partner**	Yes
**Set 2-Month Goal**	Yes
**Set 12-Month Goal**	Yes

Self-Efficacy

How confident are you in your ability to…

	3 mo.	6mo.	12 mo.
**Practice Daily**	10	8	8
**Practice Correctly**	10	10	10
**Adjust Intensity**	10	10	10
**Keep an Agenda**	10	8	10

Notes

This participant regularly reported engaging in physical activity other than HOMEX in the first half of the program. He reduced training following a series of illnesses. He did not report experiencing the positive effects of training because he did not train consistently throughout most of the program.

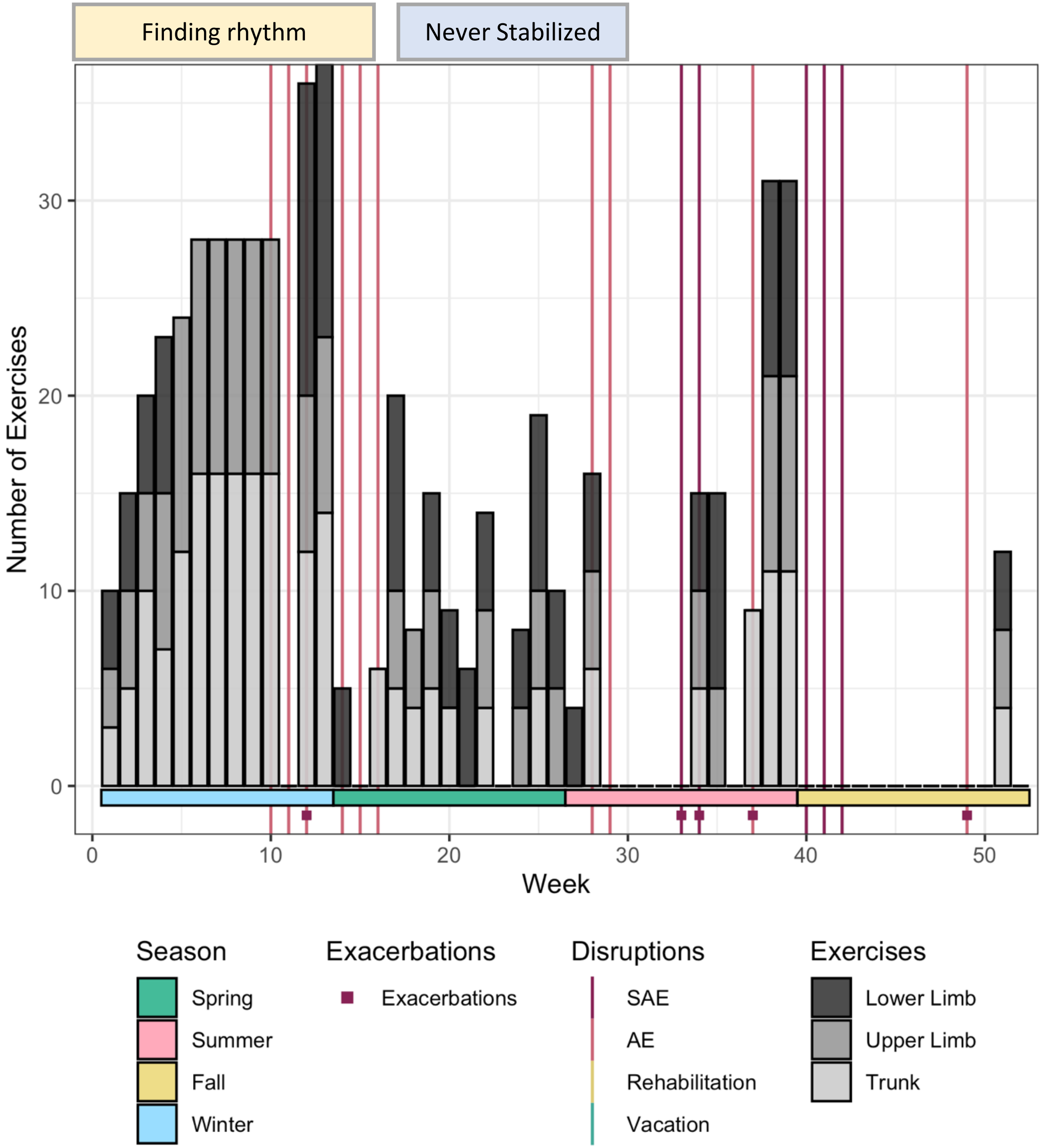

Participant 50

Adherence and Baseline Demographics

**Adherence: All Weeks (%)**	76.9
**Adherence: Healthy Weeks (%)**	90.9
**Bad Health (Weeks)**	8
**Age**	72
**Sex**	Male
**FEV1 (% Pred)**	43.1
**CRQ Dyspnea**	5.5
**Marital Status**	Married or partnership
**Living Situation**	Lives with partner or children
**Comorbidities (n)**	2
**Sparring Partner**	Yes
**Set 2-Month Goal**	Yes
**Set 12-Month Goal**	Yes

Self-Efficacy

How confident are you in your ability to…

	3 mo.	6mo.	12 mo.
**Practice Daily**	9	10	6
**Practice Correctly**	9	10	10
**Adjust Intensity**	9	8	8
**Keep an Agenda**	9	10	10

Notes

This participant did not report engaging in physical activity other than HOMEX. He stopped training due to a heart condition. He did not indicate whether he experienced positive effects but was motivated to train to increase his health and fitness.

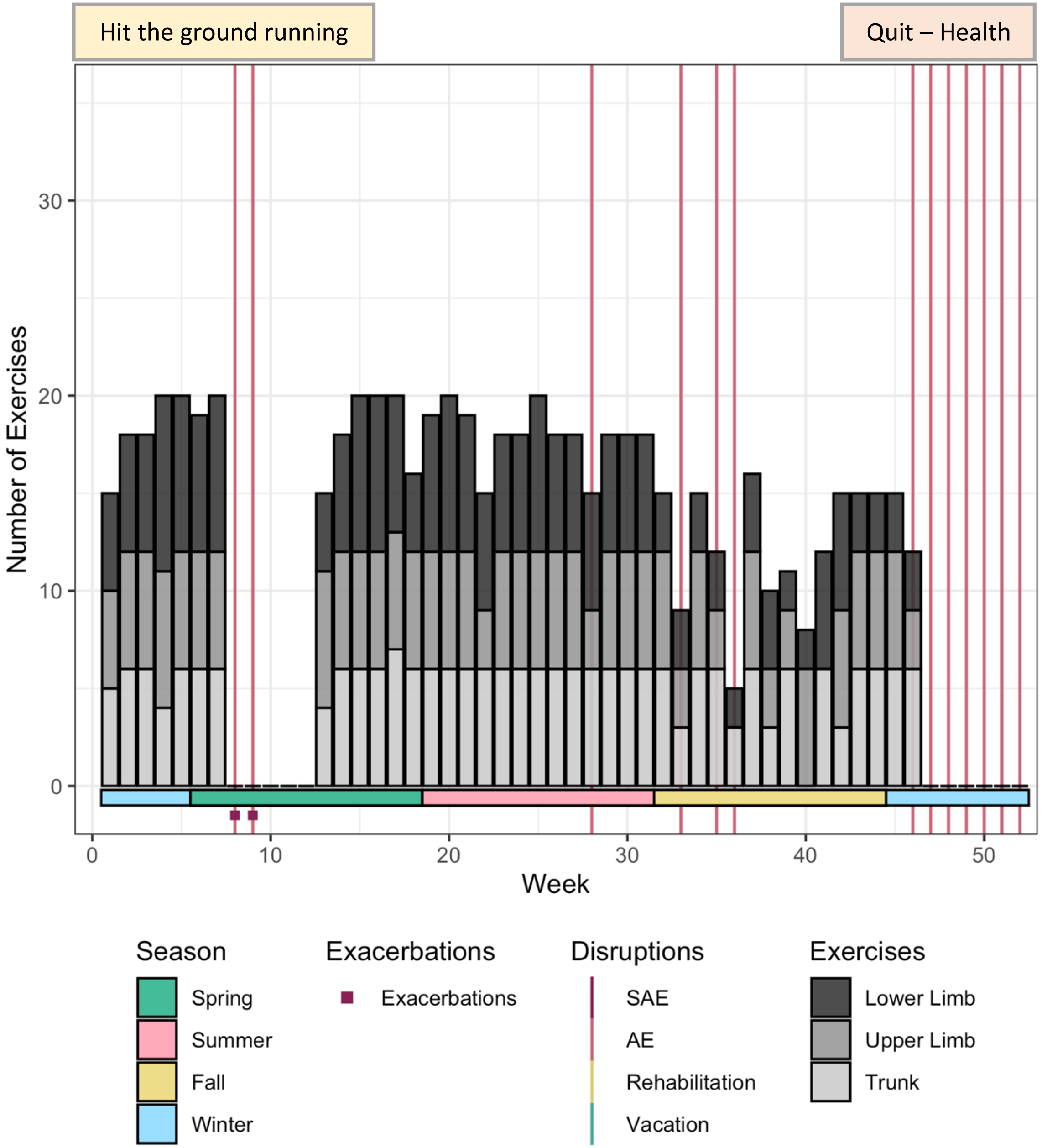

Participant 51

Adherence and Baseline Demographics

**Adherence: All Weeks (%)**	61.5
**Adherence: Healthy Weeks (%)**	78
**Bad Health (Weeks)**	11
**Age**	57
**Sex**	Female
**FEV1 (% Pred)**	64.6
**CRQ Dyspnea**	6.2
**Marital Status**	Divorced or separated
**Living Situation**	Lives with partner or children
**Comorbidities (n)**	2
**Sparring Partner**	Yes
**Set 2-Month Goal**	Yes
**Set 12-Month Goal**	Yes

Self-Efficacy

How confident are you in your ability to…

	3 mo.	6mo.	12 mo.
**Practice Daily**	9	6	8
**Practice Correctly**	9	9	10
**Adjust Intensity**	10	10	10
**Keep an Agenda**	9	8	1

Notes

This participant occasionally reported engaging in physical activity other than HOMEX. She experienced the positive effects of training in her daily life and was motivated to train by her coach.

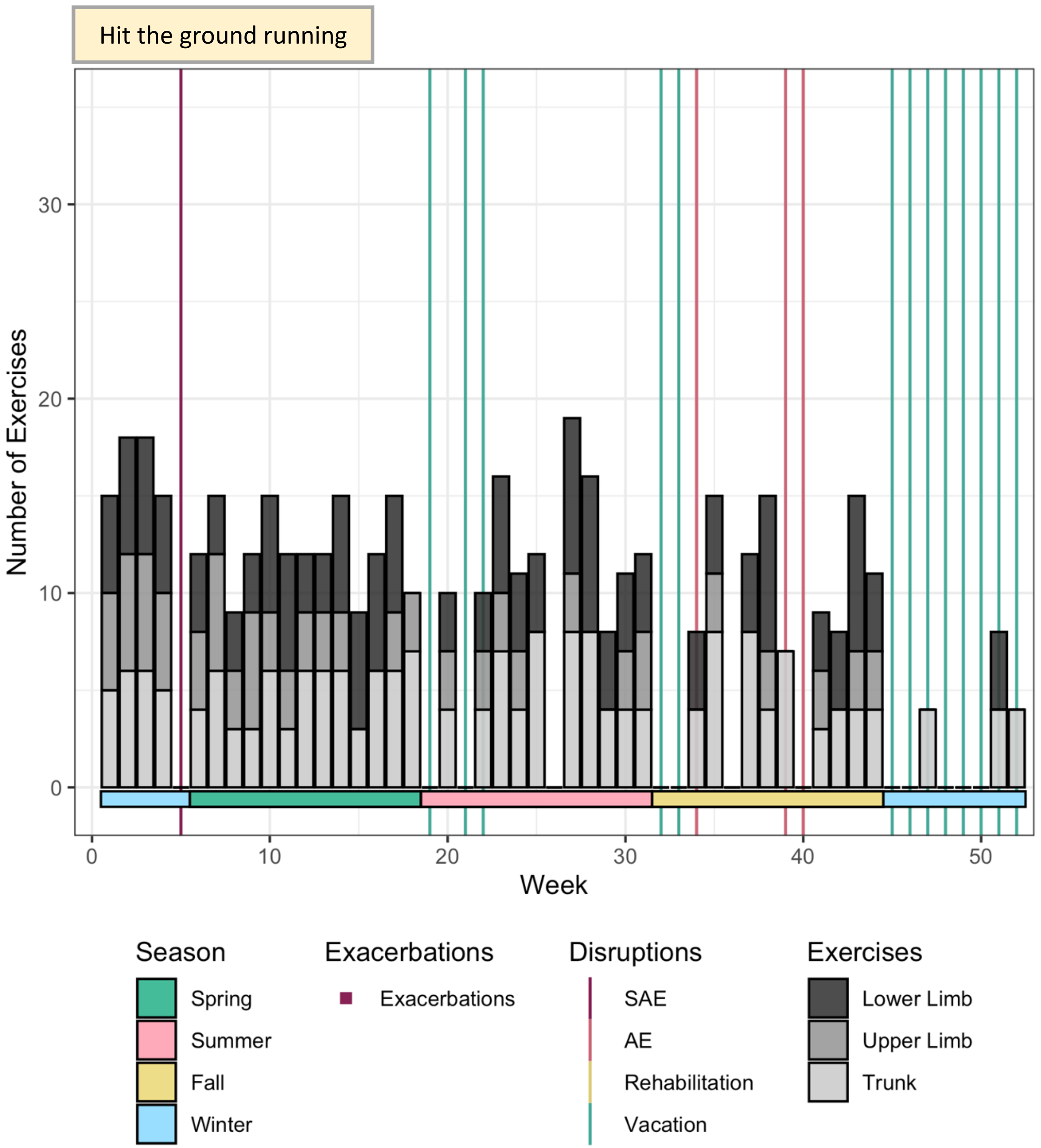

Participant 52

Adherence and Baseline Demographics

**Adherence: All Weeks (%)**	98.1
**Adherence: Healthy Weeks (%)**	97.9
**Bad Health (Weeks)**	5
**Age**	60
**Sex**	Male
**FEV1 (% Pred)**	40.9
**CRQ Dyspnea**	2.5
**Marital Status**	Divorced or separated
**Living Situation**	Lives alone
**Comorbidities (n)**	2
**Sparring Partner**	No
**Set 2-Month Goal**	Yes
**Set 12-Month Goal**	Yes

Self-Efficacy

How confident are you in your ability to…

	3 mo.	6mo.	12 mo.
**Practice Daily**	-	10	10
**Practice Correctly**	-	10	10
**Adjust Intensity**	-	10	10
**Keep an Agenda**	-	10	10

Notes

This participant did not report engaging in activities other than HOMEX throughout the year. He did not participate in the exit interviews.

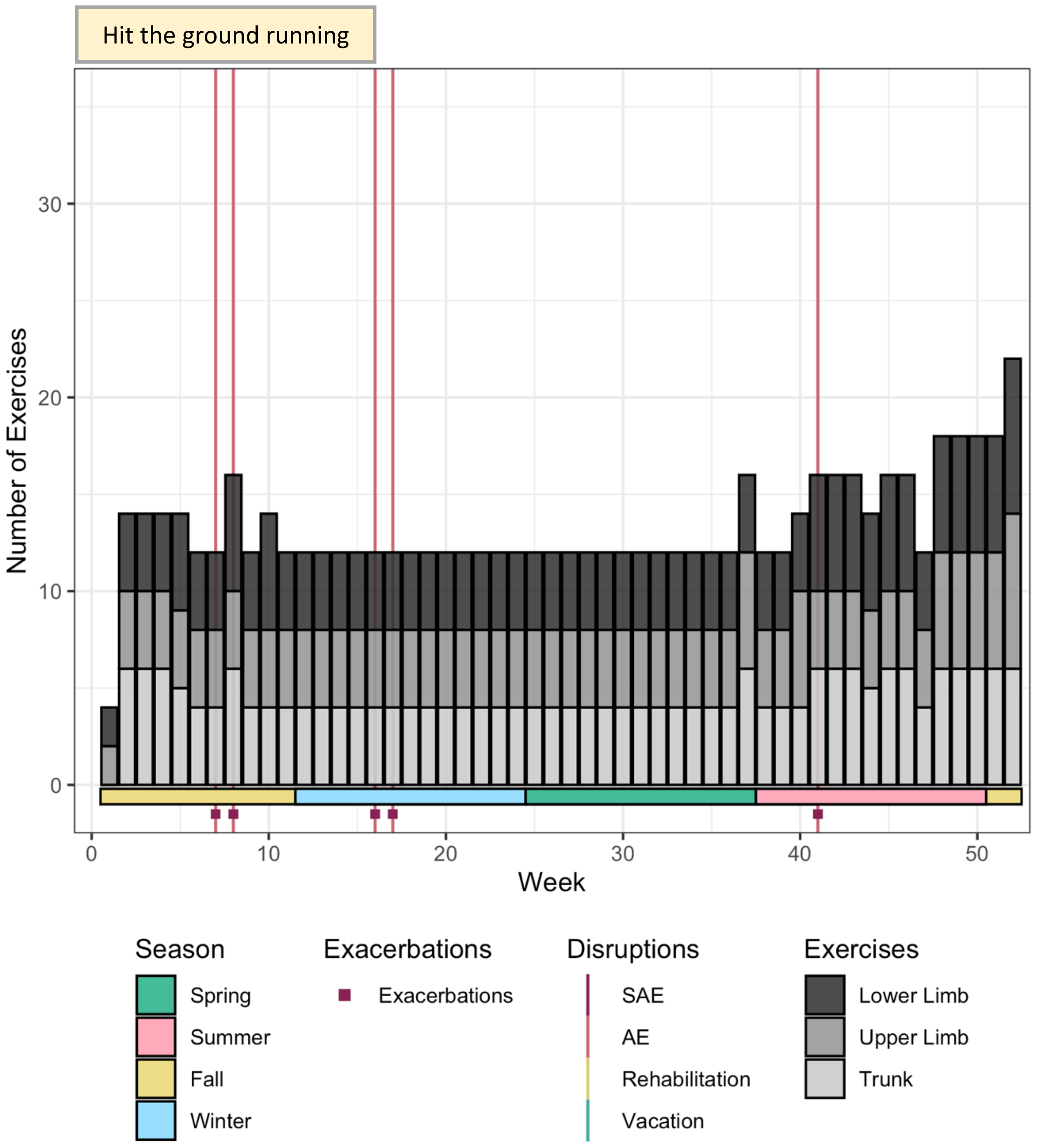

Supplement: Supplementary file 1 [file Data_Sheet_1.PDF]
